# Synthesis and In Vitro Cytotoxicity of the 4-(Halogenoanilino)-6-bromoquinazolines and Their 6-(4-Fluorophenyl) Substituted Derivatives as Potential Inhibitors of Epidermal Growth Factor Receptor Tyrosine Kinase

**DOI:** 10.3390/ph10040087

**Published:** 2017-11-20

**Authors:** Malose Jack Mphahlele, Hugues K. Paumo, Yee Siew Choong

**Affiliations:** 1 Department of Chemistry, College of Science, Engineering and Technology, University of South Africa, Private Bag X06, Florida 1710, South Africa; Tkamdeh@unisa.ac.za; 2 Institute for Research in Molecular Medicine (INFORMM), Universiti Sains Malaysia, Penang 11800, Malaysia; yeesiew@usm.my

**Keywords:** 6-bromo-4-chloroquinazolines, amination, Suzuki–Miyaura cross-coupling, cytotoxicity, EGFR-TK, molecular docking

## Abstract

Series of the 2-unsubstituted and 2-(4-chlorophenyl)–substituted 4-anilino-6-bromoquinazolines and their 6-(4-fluorophenyl)–substituted derivatives were evaluated for in vitro cytotoxicity against MCF-7 and HeLa cells. The 2-unsubstituted 4-anilino-6-bromoquinazolines lacked activity, whereas most of their 2-(4-chlorophenyl) substituted derivatives were found to exhibit significant cytotoxicity and selectivity against HeLa cells. Replacement of bromine with 4-fluorophenyl group for the 2-unsubstituted 4-anilinoquinazolines resulted in superior activity against HeLa cells compared to Gefitinib. The presence of a 4-fluorophenyl group in the 2-(4-chlorophenyl) substituted derivatives led to increased cytotoxicity against HeLa cells, except for the 3-chloroanilino derivative. The most active compounds, namely, **3g**, **3l**, and **4l**, were found to exhibit a moderate to significant inhibitory effect against epidermal growth factor receptor tyrosine kinase (EGFR-TK). The EGFR molecular docking model suggested that these compounds are nicely bound to the region of EGFR.

## 1. Introduction

**Figure 1:**
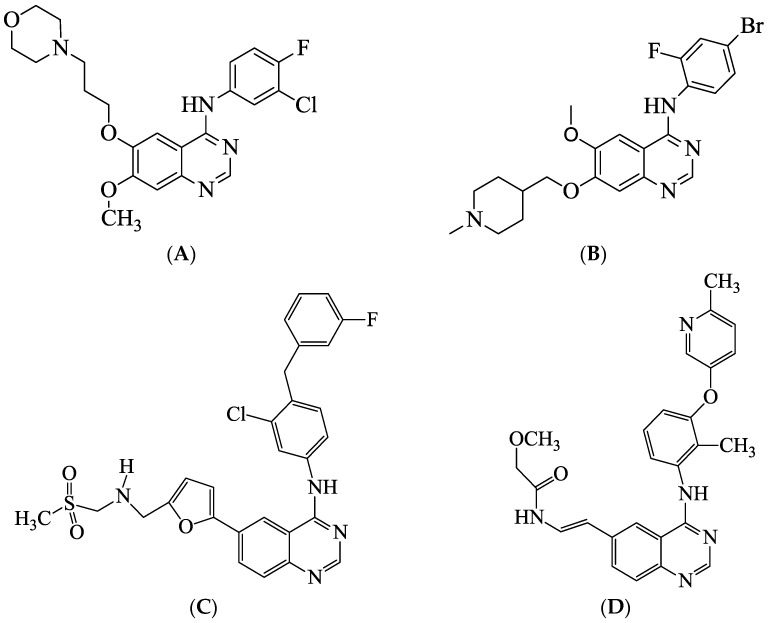
Examples of biologically relevant 4-anilinoquinazolines. Gefitinib (**A**), Vandetanib (**B**), Lapatinib (**C**), 6-vinyl substituted 4-anilinoquinazoline derivative (**D**).

The quinazoline scaffold continues to draw the attention of medicinal chemists due to its well-known anticancer properties; many substituted quinazoline derivatives have recently earned great interest in targeted therapies as antitumor drugs. Among quinazoline-based compounds, the 4-anilinoquinazolines assert themselves as selective inhibitors of the epidermal growth factor receptor tyrosine kinase (EGFR-TK) phosphorylation, which results from competitive binding at the ATP site [1,2]. The altered protein expression and activity of the receptor tyrosine kinases (TK) are implicated in the progression of various types of cancers such as breast, ovarian, colon, and prostate [3]. Gefitinib (**A**), shown in Figure 1, is an example of a trisubstituted 4-anilinoquinazoline drug used for the treatment of non-small-cell lung cancer (NSCLC) with sensitive mutations of the EGFR [4]. Likewise, Vandetanib (**B**) has been found to be a selective inhibitor that targets the vascular endothelial growth factor receptor (VEGFR) and the EGFR signal transduction pathways for the treatment of breast cancer [5]. Lapatinib (**C**), a 6-heteroaryl substituted 4-anilinoquinazoline derivative, on the other hand, is an oral dual TK inhibitor that targets both EGFR and HER2 to inhibit the proliferation of breast cancer cells [6]. The 6-vinyl substituted 4-anilinoquinazoline derivative (**D**) is a selective ERBB2 angiogenesis inhibitor under investigation for the treatment of various types of cancer [7]. Structure–activity relationship (SAR) modelling studies for the ability of 4-anilinoquinazoline to inhibit EGFR-TK activity revealed that both of the quinazoline nitrogen atoms are essential for activity (as a hydrogen bond acceptor for N1 and a water-linked hydrogen bond acceptor for N3); aniline moiety-bearing lipophilic substituents such as chloro, bromo, and trifluoromethyl groups were also important as they occupy the lipophilic pocket [8]. Although most of the medicinally important 4-anilinoquinazolines have no substitute at the C-2 position, in a previous study on the polysubstituted 2-aryl-4-(3-fluoroanilino)quinazolines from our laboratory, we observed that the presence of bromine in the 6-position and a 4-chlorophenyl group at position-2 of the quinazoline moiety favour activity against the human breast cancer (MCF-7) cell line [9]. In our quest to optimise this class of potential anti-proliferative agents, we decided to prepare the C-2 unsubstituted and the 2-(4-chlorophenyl) substituted 4-anilino-6-bromoquinazolines substituted with different halogen atoms on the aniline moiety and a 4-fluorophenyl group at the 6-position for further studies of anticancer properties.

Our approach to the target compounds involved the use of 6-bromo-4-chloroquinazoline and 2-aryl-6-bromo-4-chloroquinazolines as substrates for amination with halogenated aniline derivatives. The main aim was to evaluate the resultant 4-anilino-6-bromoquinazolines and their 6-(4-fluorophenyl) substituted derivatives for anti-proliferative effect and for their ability to inhibit EGFR tyrosine kinase phosphorylation. Their mechanism of anticancer activity is also explained theoretically through molecular (in silico) docking into the ATP binding site of EGFR to predict the hypothetical protein–ligand binding mode.

## 2. Results and Discussion

### 2.1. Synthesis of 6-(4-Fluorophenyl) Substituted 4-Anilinoquinazolines ***4a–l***

**Scheme 1:**
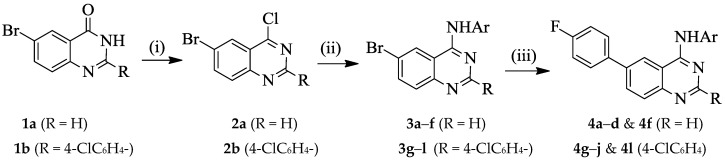
Synthesis of 4-anilino-6-bromoquinazolines and their 6-fluorophenyl derivatives. Reagents & conditions: (i) POCl_3_, Et_3_N, reflux, 2 h; (ii) NH_2_Ar, HCl, THF-iPrOH, 70 °C, 3 h; (iii) 4-FC_6_H_4_B(OH)_2_, PdCl_2_(PPh_3_)_2_, Cs_2_CO_3_, THF-iPrOH, 70 °C, 3 h.

**Scheme 2:**
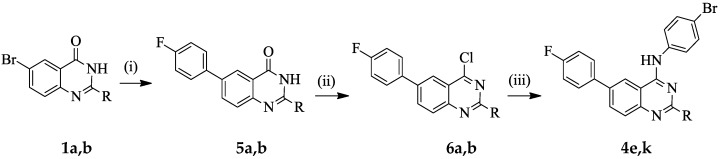
Synthesis of 4-(4-bromoanilino)-6-(4-fluorophenyl)quinazoline derivatives **4e** and **4k***.* Reagents & conditions: (i) 4-FC_6_H_4_B(OH)_2_, PdCl_2_(PPh_3_)_2_, K_2_CO_3_, dioxane (aq), reflux, 3 h; (ii) POCl_3_, Et_3_N, reflux, 5 h; (iii) 4-NH_2_C_6_H_4_Br, HCl, THF-iPrOH, 70 °C, 3 h.

It has previously been demonstrated that the presence of halogen atom(s) on the aniline ring improves the biological activity of the 4-anilinoquinazoline derivatives [4,5,8,10]. The presence of a fluorine-containing group in a molecule, on the other hand, introduces beneficial biological and pharmacological properties such as increased metabolic stability and binding ability as well as enhanced membrane permeability [11,12]. With these considerations in mind, we decided to prepare 2-unsubstituted and 2-(4-chlorophenyl) substituted 4-(halogenophenylamino)-6-bromoquinazolines and transformed them into the corresponding 6-(4-fluorophenyl)–substituted derivatives (Scheme 1 and Scheme 2). With the exception of the 4-(4-bromoanilino) substituted derivatives, **4e** and **4k**, all other 6-(4-fluorophenyl)–substituted 4-anilinoquinazolines 4 were readily prepared via initial amination of 6-bromo-4-chloroquinazoline **2a** (R = H) or 6-bromo-4-chloro-2-(4-chlorophenyl)quinazoline **2b** (R = 4-ClC_6_H_4_–) with various halogenated aniline derivatives in tetrahydrofuran-isopropanol (THF-iPrOH) mixture in the presence of HCl as a catalyst under reflux for 5 h (Scheme 1 and Table 1). The 4-anilino-6-bromoquinazoline derivatives **3a**–**l** are easily distinguished from the corresponding substrates by the presence of additional signals in the aromatic region of their proton and carbon-13 nuclear magnetic resonance (NMR) spectra (^1^H- and ^13^C-NMR) and the NH signal in the region δ_H_ 9.89–10.10 ppm. The presence of the aniline group is further confirmed by a band in the regions ν_max_ 3239–3280 (**3a**–**f**) and 3413–3456 cm^−1^ (**3g**–**l**) of their IR spectra. Moreover, the molecular ion region of their mass spectra revealed the absence of the M+ and M+2 peaks in the ratio 3:1 due to the ^35^Cl and ^37^Cl isotope observed in the spectra of the corresponding substrates, thus confirming their aniline nature. We subjected the 4-anilino-6-bromoquinazoline derivatives **3a**–**l** to Suzuki–Miyaura cross-coupling with 4-fluorophenylboronic acid as a model for C*sp*^2^–C*sp*^2^ bond formation in the presence of a dichlorobis(tricyclohexylphosphine)palladium(II) pre-catalyst and K_2_CO_3_ as a base in THF-iPrOH at 70 °C. We isolated by column chromatography on silica gel the corresponding 6-arylated 4-anilinoquinazolines **4**. Due to competitive Suzuki–Miyaura cross-coupling of the C*sp*^2^–Br bond of the 4-bromoanilino–substituted derivatives **3e** and **3k**, we opted for an alternative route for the synthesis of the corresponding 6-(4-fluorophenyl) substituted derivatives **4e** and **4k**, which is outlined in Scheme 2 and Table 2 below. The NH-4(3*H*)-oxo derivatives **1a** and **1b** were first subjected to Suzuki–Miyaura cross-coupling with 4-fluorophenylboronic acid, followed by aromatization of **5a** and **5b** under the same reaction conditions employed for the preparation of compounds **2**. Amination of the corresponding products **6a** and **6b** with 4-bromoaniline to afford **4e** and **4k** was achieved under the same reaction conditions employed for the synthesis of compounds **3**. The ^1^H- and ^13^C-NMR spectra of compounds **4a**–**l** are characterised by the presence of an increased number of proton and carbon signals in the aromatic region, thus confirming the substitution of the bromine atom. The aromatic region of their ^1^H-NMR spectra reveal the presence of two sets of intense triplets typical of the 4-fluorophenyl group. The molecular ion region of the mass spectra of compounds **4a**–**l**, on the other hand, revealed the absence of M+ and M+2 peaks in the ratio 1:1 characteristic of compounds containing the ^79^Br and ^81^Br isotopes, thus confirming their 6-carbosubstituted 4-anilinoquinazoline nature. 

### 2.2. Biological Activity Studies

A series of the analogous 6-substituted 4-aminoquinazoline derivatives have recently been evaluated as potential PI3K inhibitors and were found to exhibit significant anti-proliferative activities against the human colon cancer (HCT-116) and MCF-7 cell lines [13]. We are interested in breast and cervical cancers, which are the most common cancers in woman of all races throughout the world [14]. In this regard we decided to evaluate compounds **3a**–**l** and **4a**–**l** for potential anti-proliferative activity against two cancer cell lines, namely, the human breast adenocarcinoma (MCF-7) and cervical cancer (HeLa) cells. Since these tumours have been proven to be highly expressed cell line of EGFR, we also evaluated the most active compounds for their ability to inhibit EGFR-TK phosphorylation complemented with molecular docking into EGFR-TK.

#### 2.2.1. Cytotoxicity Studies

The 4-anilinoquinazolines **3a**–**l** and **4a**–**l** were evaluated for growth inhibitory activity against MCF-7 and HeLa cell lines using the well-established 3-(4,5-dimethylthiazole-2-yl)-2,5-diphenyltetrazoliumbromide based colorimetric cell viability (MTT) assay. The compounds were assayed at concentrations ranging from 0.1 to 100 μM with DMSO and clinical drug Gefitinib as the negative and positive control, respectively. The LC_50_ values (the lethal concentration at which 50% of cells are killed) of the 24 compounds (the average from three independent experiments) against Gefitinib as a reference drug are represented in Table 3 and Table 4 (see Supplementary Materials for the corresponding cell viability percentages and graphs for each compound). The LC_50_ values are presented in µM concentrations, taking into account the molecular weights of the compounds. The SAR of these compounds was studied with respect to the nature of substituent at the 2- or 6-position of the heterocyclic framework and the position and type of halogen atoms on the aniline ring. Some of the compounds exhibited comparable or superior anti-proliferative activities as Gefitinib against the two cancer cell lines. No activity was observed for the 2-unsubstituted 4-anilino-6-bromoquinazolines **3a**–**f** when compared to Gefinitib (Table 3). However, the presence of 4-chlorophenyl group at C-2 resulted in significant cytotoxicity against both cell lines for the corresponding 4-anilino-6-bromoquinazolines derivatives **3g**–**l**. Specifically, the 2-fluoroanilino substituted derivative **3g** and the 2,4-difluoroanilino analogue **3l** were found to exhibit higher activity against both MCF-7 and HeLa cell lines when compared to the reference drug. Moderate activity against the MCF-7 cell line (LC_50_ = 0.73 μM), but significant cytotoxicity against the HeLa cells (LC_50_ = 1.45 μM) were observed for the 3-fluoroaniline–substituted derivative **3h**. The presence of chlorine atom at position-3 of the aniline moiety in compound **3j** resulted in moderate activity for both cell lines. Diminished activity against both cell lines was observed for compounds **3i** and **3k** substituted with fluorine or bromine atom at the para-position of the aniline moiety.

Replacement of the 6-bromine atom with a 4-fluorophenyl group generally resulted in significant cytotoxicity and selectivity against the HeLa cell line for both the 2-unsubstituted **4a**–**f** and their 2-(4-chlorophenyl) substituted derivatives **4g**–**l** (Table 4). Within these series, the 4-bromoanilino substituted derivative **4e** was found to exhibit moderate (LC_50_ = 3.18 μM) cytotoxicity against the HeLa cell line, while the 3-chloroanilino substituted analogue **4j** showed reduced activity (LC_50_ = 5.24 μM) against this cell line when compared to Gefitinib (LC_50_ = 2.37 μM). Among the 2-unsubstituted derivatives **4a**–**f**, only the 3-fluoroanilino substituted derivative 4b was also found to exhibit significant cytotoxicity against the MCF-7 cell line with LC_50_ value of 0.86 μM compared to Gefitinib (LC_50_ = 0.51 μM). Likewise, within series of the 2-(4-chlorophenyl) substituted derivatives **4g**–**l**, only the 4-bromoanilino derivative **4k** and the 2,4-difluoroanilino substituted derivative **4l** were found to exhibit significant cytotoxicity against the MCF-7 cell line with LC_50_ values 0.90 μM and 0.56 μM, respectively. From these preliminary in vitro cytotoxicity results and SAR, it is observed that the presence of a 4-chlorophenyl group at the 2-position of the 4-(arylamino)quinazolines generally leads to increased cytotoxicity for both series of the 6-substituted 4-anilinoquinazoline derivatives. Increased cytotoxicity against both cell lines is generally enhanced by the presence at position-4 of a 2-fluoroanilino or 2,4-difluoroanilino group as observed for compounds **3g**, **3l** and **4l**. This is probably because a fluorophenyl group on heterocycles enhances biological activity and the lipophilicity of the molecule due to the non-polarisability of the C*sp*^2^–F bond [15].

#### 2.2.2. Inhibition of EGFR-TK

**Figure 2:**
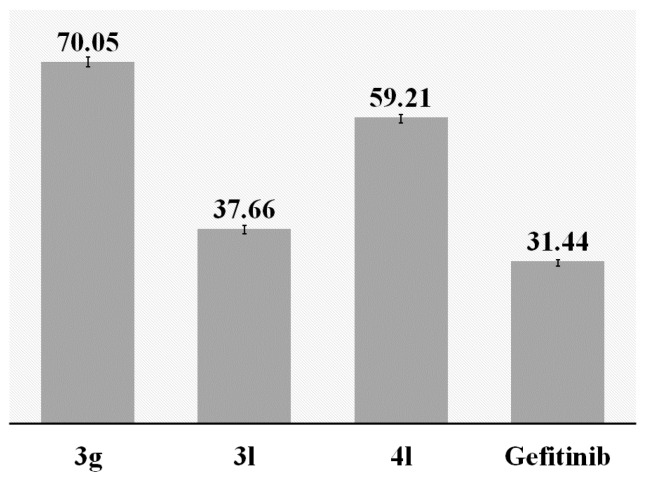
Inhibition (LC_50_ in nM) of EGFR-TK by **3g**, **3l** and **4l** against Gefitinib.

The 4-anilinoquinazolines such as Gefitinib are known to produce their anticancer activity through inhibition of EGFR-TK phosphorylation, which results from competitive binding at the ATP site [1,2]. The activity of EGFR-TK is implicated in the progression of breast, ovarian, lung, colon, prostate, and squamous cell head and neck cancers [16]. In order to test whether the 4-anilinoquinazolines prepared in this investigation inhibit the ligand binding-induced receptor phosphorylation, we performed kinase activity of EGFR in the presence of compounds **3g**, **3l** and **4l**. The inhibitory activities of compounds **3g**, **3l** and **4l** to EGFR were tested by ELISA method against Gefitinib as a reference standard. The observed results indicate that these compounds exhibit moderate to significant inhibitory effect against EGFR-TK (Figure 2). A combination of the 2-(4-chlorophenyl) and 4-(2,4-difluoroanilino) groups on the 6-bromoquinazoline framework of derivative **3l** resulted in significant inhibitory activity against EGFR-TK with LC_50_ value of 37.66 nM, which is slightly above that of the clinical drug, Gefitinib (LC_50_ = 31.44 nM). This is interesting because substitution at the C-2 position of the quinazoline ring, even with a small methyl group, was previously found to diminish activity against EGFR-TK [17]. Although a bulky group at the 6-position of the quinazoline ring can be tolerated [18], the presence of a 4-fluorophenyl group at this position in **4l** resulted in reduced EGFR-TK inhibitory (LC_50_ = 59.21 nM) compared to the corresponding 6-bromo substituted precursor **3l**. Previous SAR of the 4-anilinoquinazolines suggested that substantial (up to 10-fold) increase in potency could be achieved by the introduction of small lipophilic groups at the 3-position of the aniline ring [19,20]. We observed significantly reduced EGFR-TK inhibitory effect (LC_50_ = 70.05 nM) for **3g** substituted with a 4-(2-fluoroanilino) group when compared to the 4-(2,4-difluoroanilino)–substituted derivative **3l**.

The difference in activity related to the halogen substitution pattern on the aniline ring may be associated with the flexibility of this moiety, where a distinct spatial arrangement of the molecule could lead to an improved interaction with the active site of the receptor. In order to prove this assumption and further guide SAR studies, we performed molecular docking of compounds **3g**, **3l**, and **4l** into the ATP binding site of EGFR.

#### 2.2.3. Molecular Docking Studies against Gefitinib

**Figure 3:**
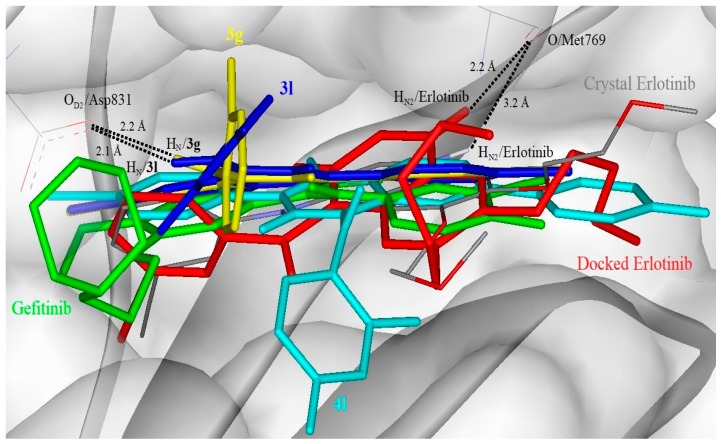
Docked conformation of Erlotinib (as control, red stick), Gefitinib (green stick), compound **3g** (yellow stick), **3l** (blue stick), and **4l** (cyan stick) in the binding pocket of epidermal growth factor receptor (EGFR) kinase domain (surface and ribbon representation). Black dotted lines are the direct hydrogen bonding formed between docked compound and EGFR. Crystal erlotinib is in a coloured atom-type stick presentation.

Docking simulation was conducted to study the binding affinity of the given compounds toward EGFR-TK. The control docked on Erlotinib (an inhibitor co-crystallized with EGFR-TK in 1M17) resulted root mean square deviation (RMSD) of 1.6 Å from the crystal structure showed the reproducibility of docking simulation (Figure 3). Compound **4l** exhibits the highest binding affinity among the docked compounds but comparable with Gefitinib, evidenced from binding free energy calculation of −10.46 and −10.74 kcal/mol, respectively (Table 5). On the other hand, Erlotinib and compounds **3g** and **3l** have a relatively lower binding free energy of −8.71, −9.14, and −8.83 kcal/mol, respectively. Hydrogen bonding was observed between Erlotinib, Gefitinib, compound **3g** and **3l**. All the hydrogen bonds were within the distance of 2.2 Å, thus showed good binding affinity with EGFR binding pocket (Figure 3). The docked conformation of Gefitinib showed the position of the morpholine region interacts with EGRK-TK Phe699 benzene ring at antiparallel orientation. The 2,4-difluorophenyl region of compounds **3l** and **4l** and the 2-fluorophenyl region of compound **3g** also showed T-shape ring stacking interactions with Phe799 benzene ring. Compound **3g** and **3l** have similar binding poses. Compound **4l**, which is bulkier in size, could have more interactions with the binding pocket residues of EGFR, thus a better binding affinity compared with compounds **3g** and **3l**. From the binding mode, it could be assumed that the synthesised substances might act on the ATP binding site of EGFR like Erlotinib or Gefitinib do.

## 3. Experimental Section

### 3.1. General

Melting points were recorded on a Thermocouple digital melting point apparatus (Mettler Toledo LLC, Columbus, OH, USA). IR spectra were recorded as powders using a Bruker VERTEX 70 FT-IR Spectrometer (Bruker Optics, Billerica, MA, USA) with a diamond ATR (attenuated total reflectance) accessory by using the thin-film method. For column chromatography, Merck kieselgel 60 (0.063–0.200 mm) (Merck KGaA, Frankfurt, Germany) was used as stationary phase. NMR spectra were obtained as CDCl_3_ solutions using Agilent 500 MHz NMR (Agilent Technologies, Oxford, UK) spectrometer and the chemical shifts are quoted relative to the TMS peak. Low- and high-resolution mass spectra were recorded at an ionisation potential of 70 eV using Waters Synapt G2 Quadrupole Time-of-flight mass spectrometer (Waters Corp., Milford, MA, USA) at the University of Stellenbosch Central Analytical Facility.

### 3.2. Typical Procedure for the Preparation of Compounds ***1a*** and ***1b***

A stirred mixture of 2-amino-5-bromobenzamide (1.0 equiv.), aldehyde (1.5 equiv.) and iodine (2.0 equiv.) in ethanol (6.0 mL/mmol of benzamide) in a round-bottomed flask equipped with a stirrer bar and a condenser was refluxed for 8 h and then allowed to cool to room temperature. An ice-cold saturated aqueous solution of sodium thiosulphate was added to the mixture and the resultant precipitate was filtered and washed with cold water. The product was recrystallized from acetonitrile. Compounds **1a** and **1b** were prepared in this fashion.

*6-Bromoquinazolin-4(3H)-one* (**1a**). A mixture of 2-amino-5-bromobenzamide (1.00 g, 4.65 mmol), paraformaldehyde (0.21 g, 6.97 mmol) and iodine (2.36 g, 9.30 mmol) in ethanol (30 mL) afforded **1a** as a white solid (0.95 g, 91%), mp. 278–280 °C (lit. [21] 273–275 °C); ν_max_ (ATR) 493, 563, 622, 824, 924, 1237, 1271, 1316, 1387, 1462, 1603, 1688, 2809, 3445 cm^−1^; δ_H_ (500 MHz, DMSO-*d*_6_) 7.59 (d, *J* = 8.5 Hz, 1H), 7.93 (dd, *J* = 2.5 and 8.5 Hz, 1H), 8.12 (s, 1H), 8.16 (d, *J* = 2.0 Hz, 1H), 12.40 (br s, 1H).

*6-Bromo-2-(4-chlorophenyl)quinazolin-4(3H)-one* (**1b**). A mixture of 2-amino-5-bromobenzamide (1.00 g, 4.65 mmol), 4-chlorobenzaldehyde (0.97 g, 6.97 mmol) and iodine (2.36 g, 9.30 mmol) in ethanol (30 mL) afforded **1b** as a white solid (1.45 g, 93%), mp. 342–344 °C; ν_max_ (ATR) 538, 559, 649, 728, 831, 940, 1091, 1276, 1413, 1460, 1556, 1601, 1672, 3442 cm^−1^; δ_H_ (500 MHz, DMSO-*d*_6_) 7.60 (d, *J* = 8.5 Hz, 2H), 7.66 (d, *J* = 8.5 Hz, 1H), 7.95 (dd, *J* = 2.5 and 8.5 Hz, 1H), 8.16 (d, *J* = 8.5 Hz, 2H), 8.19 (d, *J* = 2.0 Hz, 1H), 12.76 (brs, 1H); δ_C_ (125 MHz, DMSO-*d*_6_) 104.7, 112.5, 112.9, 119.9, 128.4, 129.2, 129.9, 130.2, 138.0, 139.1, 155.3, 159.3; *m*/*z* 335 (100, M + H); HRMS (ES): found 334.9578. C_14_H_9_^79^Br^35^ClN_2_O^+^ requires 334.9587. 

### 3.3. Typical Procedure for the Chlorination–Dehydration of ***1a*** and ***1b*** to Afford ***2a*** and ***2b***, Respectively

*Synthesis of 6-Bromo-4-chloroquinazoline* (**2a**). Triethylamine (4 mL) was added dropwise at room temperature to a stirred mixture of **1a** (0.50 g, 2.22 mmol) and phosphoryl chloride (10 mL) in a round-bottomed flask. The reaction mixture was stirred under reflux for 5 h and then allowed to cool to room temperature. Ice-cold water was added to the mixture and the product was extracted with chloroform. The combined organic layers were washed with an aqueous solution of NaHCO_3_, dried over Na_2_SO_4_, filtered, and evaporated under reduced pressure. The crude product was recrystallized from ethanol to afford **2a** as a white solid (0.46 g, 85%), mp. 164–166 °C (lit. [20] 161–163 °C); ν_max_ (ATR) 499, 539, 850, 1021, 1169, 1271, 1352, 1389, 1468, 1474, 1560, 1630, 2932 cm^−1^; δ_H_ (500 MHz, DMSO-*d*_6_) 7.95 (d, *J* = 8.5 Hz, 1H), 8.06 (dd, *J* = 2.5 and 8.5 Hz, 1H), 8.44 (d, *J* = 2.0 Hz, 1H), 9.03 (s, 1H).

*Synthesis of 6-Bromo-4-chloro-3-(4-chlorophenyl)quinazoline* (**2b**). A stirred mixture of **1b** (0.50 g, 1.49 mmol), trimethylamine (4 mL), and phosphoryl chloride (10 mL) afforded **2b** as a yellow solid (0.48 g, 91%), mp. 244–246 °C; ν_max_ (ATR) 505, 531, 732, 829, 991, 1090, 1294, 1318, 1402, 1415, 1469, 1493, 1539, 1556, 1592, 1633 cm^−1^; δ_H_ (500 MHz, DMSO-*d*_6_) 7.48 (d, *J* = 8.5 Hz, 2H), 7.93 (d, *J* = 8.0 Hz, 1H), 7.99 (dd, *J* = 2.5 and 8.5 Hz, 1H), 8.40 (d, *J* = 2.0 Hz, 1H), 8.51 (d, *J* = 8.5 Hz, 2H); δ_C_ (125 MHz, DMSO-*d*_6_) 104.8, 112.5, 112.9, 128.5, 129.2, 129.9, 130.2, 138.0, 139.2, 149.1, 152.0 159.3; *m*/*z* (100, M + H); HRMS (ES): found 334.9571. C_14_H_9_^79^Br^35^Cl_2_^+^ requires 334.9587.

### 3.4. Typical Procedure for the Amination of ***2a*** and ***2b*** to Afford Compounds ***3a***–***f*** and ***3g***–***l***, Respectively

*6-Bromo-N-(2-fluorophenyl)quinazolin-4-amine* (**3a**). A mixture of **2a** (0.50 g, 2.05 mmol), 2-fluoroaniline (0.25 g, 2.25 mmol) and concentrated HCl (0.01 g, 0.27 mmol) in 3:1 THF-isopropanol (*v*/*v*, 10 mL) in a round-bottomed flask was stirred under reflux for 5 h. The reaction mixture was then allowed to cool to room temperature and quenched with ice-cold water. The product was extracted with ethyl acetate and the combined organic layers were washed thoroughly with an aqueous solution of NaHCO_3_, dried over anhydrous MgSO_4_, filtered, and evaporated under reduced pressure to afford **3a** as a white solid (0.52 g, 80%), mp. 174–176 °C (acetonitrile); ν_max_ (ATR) 493, 614, 757, 832, 926, 1188, 1253, 1404, 1454, 1563, 1619, 2849, 2916, 3259 cm^−1^; δ_H_ (500 MHz, DMSO-*d*_6_) 7.23‒7.32 (m, 3H), 7.52 (t, *J* = 8.0 Hz, 1H), 7.72 (d, *J* = 8.5 Hz, 1H), 7.97 (dd, *J* = 2.0 and 9.0 Hz, 1H), 8.49 (s, 1H), 8.76 (d, *J* = 2.0 Hz, 1H), 9.91 (s, 1H); δ_C_ (125 MHz, DMSO-*d*_6_) 116.5 (d, ^2^*J*_CF_ = 20.0 Hz), 119.2, 124.9 (d, ^4^*J*_CF_ = 2.8 Hz), 126.0, 126.3, 126.5 (d, ^3^*J*_CF_ = 12.3 Hz), 126.5, 128.0 (d, ^3^*J*_CF_ = 6.6 Hz), 128.8, 130.5, 136.6, 148.9, 155.5, 157.2 (d, ^1^*J*_CF_ = 242.7 Hz); *m*/*z* 318 (100, M+H); HRMS (ES): found 318.0044. C_14_H_10_BrFN_3_^+^ requires 318.0042. 

*6-Bromo-N-(3-fluorophenyl)quinazolin-4-amine* (**3b**). A mixture of **2a** (0.50 g, 2.05 mmol), 3-fluoroaniline (0.25 g, 2.25 mmol) and concentrated HCl (0.01 g, 0.27 mmol) in THF-isopropanol (10 mL) afforded **3b** as a white solid (0.49 g, 76%), mp. 214–216 °C (acetonitrile); ν_max_ (ATR) 494, 684, 778, 832, 843, 958, 1145, 1403, 1485, 1531, 1562, 1614, 3074, 3263 cm^−1^; δ_H_ (500 MHz, DMSO-*d*_6_) 6.94 (td, *J* = 2.0 and 8.5 Hz, 1H), 7.41 (q, *J* = 8.0 Hz, 1H), 7.66 (d, *J* = 8.0 Hz, 1H), 7.74 (d, *J* = 8.0 Hz, 1H), 7.92 (dt, *J* = 2.0 and 11.5 Hz, 1H), 7.99 (dd, *J* = 2.5 and 8.5 Hz, 1H), 8.67 (s, 1H), 8.86 (d, *J* = 2.0 Hz, 1H), 9.94 (s, 1H); δ_C_ (125 MHz, DMSO-*d*_6_) 109.1 (d, ^2^*J*_CF_ = 25.5 Hz), 110.5 (d, ^2^*J*_CF_ = 20.8 Hz), 116.9, 118.0 (d, ^4^*J*_CF_ = 2.8 Hz), 119.4, 125.8, 130.4 (d, ^3^*J*_CF_ = 9.5 Hz), 130.6, 136.6, 141.2 (d, ^3^*J*_CF_ = 11.3 Hz), 149.0, 155.1, 157.1, 162.4 (d, ^1^*J*_CF_ = 239.8 Hz); *m*/*z* 318 (100, M + H); HRMS (ES): found 318.0043. C_14_H_10_BrFN_3_^+^ requires 318.0042.

*6-Bromo-N-(4-fluorophenyl)quinazolin-4-amine* (**3c**). A mixture of **2a** (0.50 g, 2.05 mmol), 4-fluoroaniline (0.25 g, 2.25 mmol) and concentrated HCl (0.01 g, 0.27 mmol) in THF-isopropanol (10 mL) afforded **3c** as a white solid (0.55 g, 85%), mp. 217–218 °C (acetonitrile); ν_max_ (ATR) 497, 513, 830, 922, 1156, 1213, 1318, 1420, 1489, 1508, 1564, 1619, 2925, 3059 cm^−1^; δ_H_ (500 MHz, DMSO-*d*_6_) 7.23 (t, *J* = 8.5 Hz, 2H), 7.70 (d, *J* = 9.0 Hz, 1H), 7.82 (t, *J* = 8.5 Hz, 2H), 7.96 (dd, *J* = 2.5 and 9.0 Hz, 1H), 8.57 (s, 1H), 8.81 (d, *J* = 2.0 Hz, 1H), 9.87 (s, 1H); δ_C_ (125 MHz, DMSO-*d*_6_) 115.5 (d, ^2^*J*_CF_ = 22.7 Hz), 116.8, 119.2, 124.8 (d, ^3^*J*_CF_ = 7.6 Hz), 125.8, 130.5, 135.6 (d, ^4^*J*_CF_ = 2.8 Hz), 136.5, 149.0, 155.3, 157.3, 159.0 (d, ^1^*J*_CF_ = 239.8 Hz); *m*/*z* 318 (100, M + H); HRMS (ES): found 318.0027. C_14_H_10_BrFN_3_^+^ requires 318.0042. 

*6-Bromo-N-(3-chlorophenyl)quinazolin-4-amine* (**3d**). A mixture of **2a** (0.50 g, 2.05 mmol), 3-chloroaniline (0.29 g, 2.25 mmol) and concentrated HCl (0.01 g, 0.27 mmol) in THF-isopropanol (10 mL) afforded **3d** as a white solid (0.54 g, 79%), mp. 220–222 °C (acetonitrile); ν_max_ (ATR) 494, 683, 778, 832, 864, 958, 1145, 1354, 1403, 1485, 1531, 1562, 1614, 2935, 3074 cm^−1^; δ_H_ (500 MHz, DMSO-*d*_6_) 7.17 (d, *J* = 8.0 Hz, 1H), 7.41 (t, *J* = 8.0 Hz, 1H), 7.73 (d, *J* = 9.0 Hz, 1H), 7.82 (d, *J* = 8.5 Hz, 1H), 7.98 (dd, *J* = 2.0 and 8.5 Hz, 1H), 8.08 (s, 1H), 8.67 (s, 1H), 8.85 (d, *J* = 2.5 Hz, 1H), 9.91 (s, 1H); δ_C_ (125 MHz, DMSO-*d*_6_) 116.9, 119.4, 120.7, 121.8, 123.8, 125.8, 130.5, 130.6, 133.2, 136.6, 141.0, 149.0, 155.1, 157.1; *m*/*z* 334 (100, M+H); HRMS (ES): found 333.9747. C_14_H_10_BrClN_3_^+^ requires 333.9747.

*6-Bromo-N-(4-bromophenyl)quinazolin-4-amine* (**3e**). A mixture of **2a** (0.50 g, 2.05 mmol), 4-bromoaniline (0.39 g, 2.25 mmol) and concentrated HCl (0.01 g, 0.27 mmol) in THF-isopropanol (10 mL) afforded **3e** as a white solid (0.60 g, 77%), mp. 220–222 °C (acetonitrile); ν_max_ (ATR) 492, 621, 810, 820, 831, 924, 1007, 1066, 1351, 1406, 1487, 1521, 1560, 1618, 3105, 3280 cm^−1^; δ_H_ (500 MHz, DMSO-*d*_6_) 7.55 (d, *J* = 8.5 Hz, 2H), 7.71 (d, *J* = 9.0 Hz, 1H), 7.85 (d, *J* = 8.5 Hz, 2H), 7.96 (dd, *J* = 2.0 and 8.5 Hz, 1H), 8.62 (s, 1H), 8.83 (d, *J* = 2.0 Hz, 1H), 9.90 (s, 1H); δ_C_ (125 MHz, DMSO-*d*_6_) 116.0, 116.9, 119.3, 124.5, 125.9, 130.5, 131.7, 136.5, 138.8, 149.0, 155.1, 157.0; m/z 378 (100, M+H); HRMS (ES): found 377.9241. C_14_H_10_^79^Br_2_N_3_^+^ requires 377.9241.

*6-Bromo-N-(2,4-difluorophenyl)quinazolin-4-amine* (**3f**). A mixture of **2a** (0.50 g, 2.05 mmol), 2.4-difluoroaniline (0.29 g, 2.25 mmol) and concentrated HCl (0.01 g, 0.27 mmol) in THF-isopropanol (10 mL) afforded **3f** as a white solid (0.55 g, 79%), mp. 198–200 °C (acetonitrile); ν_max_ (ATR) 498, 536, 779, 835, 967, 1142, 1256, 1319, 1354, 1401, 1493, 1508, 1571, 3039 cm^−1^; δ_H_ (500 MHz, DMSO-*d*_6_) 7.14 (td, *J* = 2.0 and 9.0 Hz, 1H), 7.36 (td, *J* = 2.5 and 9.0 Hz, 1H), 7.53 (t, *J* = 9.0 Hz, 1H), 7.72 (d, *J* = 8.5 Hz, 1H), 7.97 (dd, *J* = 2.0 and 9.0 Hz, 1H), 8.48 (s, 1H), 8.73 (d, *J* = 2.0 Hz, 1H), 9.89 (s, 1H); δ_C_ (125 MHz, DMSO-*d*_6_) 105.0 (t, ^2^*J*_CF_ = 26.5 Hz), 111.8 (dd, *J*_CF_ = 3.7 and 21.7 Hz), 111.9 (d, ^4^*J*_CF_ = 3.8 Hz), 116.5, 119.2, 125.9, 129.9 (d, ^3^*J*_CF_ = 9.6 Hz), 130.5, 136.6, 148.9, 155.5, 158.3, 160.5 (d, ^1^*J*_CF_ = 243.6 Hz), 160.6 (d, ^1^*J*_CF_ = 243.6 Hz); *m*/*z* 336 (100, M + H); HRMS (ES): found 335.9952. C_14_H_9_BrF_2_N_3_^+^ requires 335.9948.

*6-Bromo-2-(4-chlorophenyl)-N-(2-fluorophenyl)quinazolin-4-amine* (**3g**). A mixture of **2b** (0.50 g, 1.41 mmol), 2-fluoroaniline (0.17 g, 1.55 mmol) and concentrated HCl (0.01 g, 0.27 mmol) in THF-isopropanol (10 mL) afforded **3g** as a white solid (0.49 g, 82%), mp. 248–250 °C (acetonitrile); ν_max_ (ATR) 534, 733, 782, 829, 1012, 1089, 1171, 1316, 1399, 1446, 1489, 1522, 1554, 1598, 1622, 3451 cm^−1^; δ_H_ (500 MHz, DMSO-*d*_6_) 7.30‒7.41 (m, 3H), 7.50 (d, *J* = 8.5 Hz, 2H), 7.64 (td, *J* = 2.5 and 9.0 Hz, 1H), 7.79 (d, *J* = 8.5 Hz, 1H), 8.00 (dd, *J* = 2.0 and 8.5 Hz, 1H), 8.23 (d, *J* = 8.5 Hz, 2H), 8.80 (d, *J* = 2.0 Hz, 1H), 10.05 (s, 1H); δ_C_ (125 MHz, DMSO-*d*_6_) 115.4, 116.4 (d, ^2^*J*_CF_ = 19.9 Hz), 119.0, 124.8 (d, ^4^*J*_CF_ = 2.8 Hz), 126.1, 126.3 (d, ^3^*J*_CF_ = 12.3 Hz), 127.9 (d, ^3^*J*_CF_ = 8.5 Hz), 128.3, 128.9, 129.9, 130.7, 135.8, 136.8, 137.1, 149.7, 157.0 (d, ^1^*J*_CF_ = 245.6 Hz), 158.2, 158.9; *m*/*z* 428 (100, M + H); HRMS (ES): found 427.9978. C_20_H_13_^35^ClF^79^BrN_3_^+^ requires 427.9965. 

*6-Bromo-2-(4-chlorophenyl)-N-(3-fluorophenyl)quinazolin-4-amine* (**3h**). A mixture of **2b** (0.50 g, 1.41 mmol), 3-fluoroaniline (0.17 g, 1.55 mmol) and concentrated HCl (0.01 g, 0.27 mmol) in THF-isopropanol (10 mL) afforded **3h** as a white solid (0.53 g, 88%), mp. 242–244 °C (acetonitrile); ν_max_ (ATR) 423, 487, 535, 670, 758, 824, 864, 1010, 1087, 1351, 1397, 1417, 1488, 1522, 1555, 3431 cm^−1^; δ_H_ (500 MHz, DMSO-*d*_6_) 7.00 (td, *J* = 2.0 and 8.0 Hz, 1H), 7.49 (q, *J* = 8.0 Hz, 1H), 7.59 (d, *J* = 8.5 Hz, 2H), 7.74 (dd, *J* = 2.0 and 8.0 Hz, 1H), 7.84 (d, *J* = 8.5 Hz, 1H), 7.90 (dt, *J* = 2.5 and 11.5 Hz, 1H), 8.00 (dd, *J* = 2.5 and 8.5 Hz, 1H), 8.38 (d, *J* = 8.5 Hz, 2H), 8.86 (d, *J* = 2.0 Hz, 1H), 10.07 (s, 1H); δ_C_ (125 MHz, DMSO-*d*_6_) 109.2 (d, ^2^*J*_CF_ = 26.6 Hz), 110.7 (d, ^2^*J*_CF_ = 20.8 Hz), 115.7, 118.1 (d, ^4^*J*_CF_ = 2.8 Hz), 119.2, 125.9, 129.0, 130.0, 130.5 (d, ^3^*J*_CF_ = 9.3 Hz), 130.8, 135.9, 136.9, 137.2, 141.2 (d, ^3^*J*_CF_ = 11.3 Hz), 149.7, 157.4, 158.8, 162.4 (d, ^1^*J*_CF_ = 239.0 Hz); *m*/*z* 428 (100, M + H); HRMS (ES): found 427.9966. C_20_H_13_^35^ClF^79^BrN_3_^+^ requires 427.9965. 

*6-Bromo-2-(4-chlorophenyl)-N-(4-fluorophenyl)quinazolin-4-amine* (**3i**). A mixture of **2b** (0.50 g, 1.41 mmol), 4-fluoroaniline (0.17 g, 1.55 mmol) and concentrated HCl (0.01 g, 0.27 mmol) in THF-isopropanol (10 mL) afforded **3i** as a white solid (0.51 g, 84%), mp. 289–291 °C (acetonitrile); ν_max_ (ATR) 424, 534, 669, 736, 827, 1011, 1087, 1199, 1314, 1352, 1398, 1416, 1522, 1556, 3431 cm^−1^; δ_H_ (500 MHz, DMSO-*d*_6_) 7.23 (t, *J* = 8.5 Hz, 2H), 7.55 (d, *J* = 8.5 Hz, 2H), 7.77 (d, *J* = 9.0 Hz, 1H), 7.88 (t, *J* = 8.5 Hz, 2H), 7.97 (dd, *J* = 2.5 and 9.0 Hz, 1H), 8.35 (d, *J* = 9.0 Hz, 2H), 8.82 (d, *J* = 2.0 Hz, 1H), 10.00 (s, 1H); δ_C_ (125 MHz, DMSO-*d*_6_) 115.6 (d, ^2^*J*_CF_ = 21.7 Hz), 115.7, 119.0, 124.8 (d, ^3^*J*_CF_ = 7.5 Hz), 125.9, 129.0, 130.1, 130.7, 135.6 (d, ^4^*J*_CF_ = 2.8 Hz), 135.8, 136.7, 137.2, 149.7, 157.5, 158.9, 159.0 (d, ^1^*J*_CF_ = 239.0 Hz); *m*/*z* 428 (100, M + H); HRMS (ES): found 427.9951. C_20_H_13_^35^ClF^79^BrN_3_^+^ requires 427.9965.

*6-Bromo-2-(4-chlorophenyl)-N-(3-chlorophenyl)quinazolin-4-amine* (**3j**). A mixture of **2b** (0.50 g, 1.41 mmol), 3-chloroaniline (0.19 g, 1.55 mmol) and concentrated HCl (0.01 g, 0.27 mmol) in THF-isopropanol (10 mL) afforded **3j** as a white solid (0.55 g, 87%), mp. 261–263 °C (acetonitrile); ν_max_ (ATR) 484, 535, 673, 735, 765, 829, 996, 1086, 1353, 1397, 1416, 1428, 1515, 1564, 1595, 3432 cm^−1^; δ_H_ (500 MHz, DMSO-*d*_6_) 7.20 (d, *J* = 8.5 Hz, 1H), 7.46 (t, *J* = 8.0 Hz, 1H), 7.55 (d, *J* = 8.5 Hz, 2H), 7.77 (d, *J* = 8.5 Hz, 1H), 7.88 (dd, *J* = 2.5 and 8.5 Hz, 1H), 7.97 (dd, *J* = 2.0 and 8.5 Hz, 1H), 8.13 (s, 1H), 8.82 (d, *J* = 2.0 Hz, 1H), 10.00 (s, 1H); δ_C_ (125 MHz, DMSO-*d*_6_) 115.7, 119.3, 120.7, 122.0, 123.8, 125.9, 129.0, 130.0, 130.6, 130.7, 133.2, 135.9, 136.8, 137.1, 140.9, 149.7, 157.3, 158.7; *m*/*z* 444 (100, M + H); HRMS (ES): found 443.9684. C_20_H_13_^35^Cl_2_^79^BrN_3_^+^ requires 443.9670.

*6-Bromo-N-(4-bromophenyl)-2-(4-chlorophenyl)quinazolin-4-amine* (**3k**). A mixture of **2b** (0.50 g, 1.41 mmol), 4-bromoaniline (0.27 g, 1.55 mmol) and concentrated HCl (0.01 g, 0.27 mmol) in THF-isopropanol (10 mL) afforded **3k** as a white solid (0.57 g, 83%), mp. 263–265 °C (acetonitrile); ν_max_ (ATR) 488, 541, 802, 814, 834, 875, 1011, 1083, 1351, 1400, 1489, 1520, 1554, 1564, 1596, 3434 cm^−1^; δ_H_ (500 MHz, DMSO-*d*_6_) 7.55 (d, *J* = 8.5 Hz, 2H), 7.63 (d, *J* = 8.5 Hz, 2H), 7.76 (d, *J* = 9.0 Hz, 1H), 7.88 (d, *J* = 8.5 Hz, 2H), 7.97 (dd, *J* = 2.0 and 8.5 Hz, 1H), 8.37 (d, *J* = 8.5 Hz, 2H), 8.82 (d, *J* = 2.0 Hz, 1H), 10.01 (s, 1H); δ_C_ (125 MHz, DMSO-*d*_6_) 115.7, 116.1, 119.1, 124.5, 125.9, 129.0, 130.1, 130.7, 131.8, 135.8, 136.8, 137.1, 138.8, 149.7, 157.4, 158.9; *m*/*z* 488 (100, M + H); HRMS (ES): found 487.9161. C_20_H_13_Cl^79^Br_2_N_3_^+^ requires 487.9165.

*6-Bromo-2-(4-chlorophenyl)-N-(2,4-difluorophenyl)quinazolin-4-amine* (**3l**). A mixture of **2b** (0.50 g, 1.41 mmol), 2.4-difluoroaniline (0.2 g, 1.55 mmol) and concentrated HCl (0.01 g, 0.27 mmol) in THF-isopropanol (10 mL) afforded **3l** as a white solid (0.58 g, 93%), mp. 278–279 °C (acetonitrile); ν_max_ (ATR) 425, 533, 735, 795, 830, 1010, 1088, 1202, 1257, 1351, 1418, 1498, 1528, 1558, 3456 cm^−1^; δ_H_ (500 MHz, DMSO-*d*_6_) 7.00 (td, *J* = 2.5 and 8.0 Hz, 1H), 7.44 (dd, *J* = 2.0 and 8.5 Hz, 1H), 7.51 (d, *J* = 8.5 Hz, 2H), 7.66 (q, *J* = 8.0 Hz, 1H), 7.79 (d, *J* = 8.5 Hz, 1H), 8.00 (dd, *J* = 2.0 and 8.5 Hz, 1H), 8.22 (d, *J* = 8.5 Hz, 2H), 8.78 (d, *J* = 2.5 Hz, 1H), 10.07 (s, 1H); δ_C_ (125 MHz, DMSO-*d*_6_) 104.9 (t, ^2^*J*_CF_ = 26.6 Hz), 111.8 (dd, *J*_CF_ = 2.8 and 21.7 Hz), 115.4, 119.0, 122.9 (d, ^4^*J*_CF_ = 3.8 Hz), 126.1, 128.9, 129.6 (d, ^3^*J*_CF_ = 9.5 Hz), 129.9, 130.7, 135.8, 136.9, 137.1, 149.6, 157.3 (d, ^1^*J*_CF_ = 248.4 Hz), 158.4, 158.9, 160.5 (d, ^1^*J*_CF_ = 243.7 Hz); *m*/*z* 446 (100, M + H); HRMS (ES): found 445.9854. C_20_H_12_^35^ClF_2_^79^BrN_3_^+^ requires 445.9871.

### 3.5. Typical Procedure for the Preparation of Compounds ***4a***–***d***, ***f*** and ***4g***–***j***, ***l***

*[6-(4-Fluorophenyl)quinazolin-4-yl]-2-fluorophenylamine* (**4a**). A stirred mixture of **3a** (0.30 g, 0.94 mmol), PdCl_2_(PPh_3_)_2_ (0.033 g, 0.047 mmol) and K_2_CO_3_ (0.19 g, 1.41 mmol) in 3:1 THF-isopropanol (*v*/*v*, 10 mL) in a two-necked round-bottomed flask was purged with argon gas for 30 min. 4-Fluorophenylboronic acid (0.15 g, 1.13 mmol) was added to the mixture via a syringe. The reaction mixture was heated at 100 °C for 3 h and then quenched with ice-cold water. The product was extracted into chloroform and the combined organic layers were washed with water, dried over Na_2_SO_4_, filtered and evaporated under reduced pressure. The residue was purified by column chromatography on silica gel to afford **4a** as a white solid (0.20 g, 64%), mp. 192–194 °C; R_f_ (1:1, toluene/ethyl acetate) 0.54; ν_max_ (ATR) 495, 592, 666, 746, 826, 1101, 1164, 1238, 1325, 1402, 1419, 1500, 1520, 1576, 1600, 3195 cm^−1^; δ_H_ (500 MHz, DMSO-*d*_6_) 7.24‒7.34 (m, 3H), 7.38 (t, *J* = 8.5 Hz, 2H), 7.55 (t, *J* = 8.5 Hz, 1H), 7.84 (d, *J* = 8.5 Hz, 1H), 7.91 (t, *J* = 8.0 Hz, 2H), 8.19 (dd, *J* = 2.0 and 8.5 Hz, 1H), 8.47 (s, 1H), 8.78 (d, *J* = 2.0 Hz, 1H), 9.99 (s, 1H); δ_C_ (125 MHz, DMSO-*d*_6_) 115.5, 116.3 (d, ^2^*J*_CF_ = 20.8 Hz), 116.5 (d, ^2^*J*_CF_ = 19.0 Hz), 120.9, 124.9 (d, ^4^*J*_CF_ = 2.8 Hz), 126.5 (d, ^3^*J*_CF_ = 12.3 Hz), 127.9 (d, ^3^*J*_CF_ = 7.5 Hz), 128.8 (d, ^4^*J*_CF_ = 3.7 Hz), 128.9, 129.4 (d, ^3^*J*_CF_ = 7.6 Hz), 132.1, 135.9 (d, ^4^*J*_CF_ = 2.8 Hz), 137.3, 149.4, 155.2, 157.3 (d, ^1^*J*_CF_ = 245.6 Hz), 159.2, 162.6 (d, ^1^*J*_CF_ = 243.6 Hz); *m*/*z* 334 (100, M + H); HRMS (ES): found 334.1166. C_20_H_14_F_2_N_3_^+^ requires 334.1156.

*[6-(4-Fluorophenyl)quinazolin-4-yl]-3-fluorophenylamine* (**4b**). A mixture of **3b** (0.30 g, 0.94 mmol), PdCl_2_(PPh_3_)_2_ (0.033 g, 0.047 mmol), 4-fluorophenylboronic acid (0.15 g, 1.13 mmol) and K_2_CO_3_ (0.19 g, 1.41 mmol) in THF-isopropanol (10 mL) afforded **4b** as a white solid (0.22 g, 72%), mp. 225–227 °C; R_f_ (1:1, toluene/ethyl acetate) 0.70; ν_max_ (ATR) 506, 587, 687, 780, 827, 859, 962, 1144, 1240, 1400, 1416, 1488, 1532, 1573, 1601, 3254 cm^−1^; δ_H_ (500 MHz, DMSO-*d*_6_) 6.96 (td, *J* = 2.0 and 8.5 Hz, 1H), 7.41 (t, *J* = 8.5 Hz, 2H), 7.44 (q, *J* = 8.5 Hz, 1H), 7.67 (d, *J* = 1.5 and 8.5 Hz, 1H), 7.86 (d, *J* = 8.5 Hz, 1H), 7.90–7.96 (m, 3H), 818 (dd, *J* = 2.0 and 8.5 Hz, 1H), 8.65 (s, 1H), 8.80 (d, *J* = 2.0 Hz, 1H), 10.01 (s, 1H); δ_C_ (125 MHz, DMSO-*d*_6_) 109.4 (d, ^2^*J*_CF_ = 25.5 Hz), 110.5 (d, ^2^*J*_CF_ = 20.9 Hz), 115.7, 116.3 (d, ^2^*J*_CF_ = 20.9 Hz), 118.2 (d, ^4^*J*_CF_ = 2.8 Hz), 120.8, 128.9, 129.7 (d, ^3^*J*_CF_ = 9.5 Hz), 130.4 (d, ^3^*J*_CF_ = 9.5 Hz), 132.4, 136.0 (d, ^4^*J*_CF_ = 3.8 Hz), 137.7, 141.3 (d, ^3^*J*_CF_ = 10.4 Hz), 149.4, 154.7, 158.1, 162.4 (d, ^1^*J*_CF_ = 239.8 Hz), 162.6 (d, ^1^*J*_CF_ = 243.7 Hz); *m*/*z* 334 (100, M + H); HRMS (ES): found 334.1158. C_20_H_14_F_2_N_3_^+^ requires 334.1156. 

*[6-(4-Fluorophenyl)quinazolin-4-yl]-4-fluorophenylamine* (**4c**). A mixture of **3c** (0.30 g, 0.94 mmol), PdCl_2_(PPh_3_)_2_ (0.033 g, 0.047 mmol), 4-fluorophenylboronic acid (0.15 g, 1.13 mmol) and K_2_CO_3_ (0.19 g, 1.41 mmol) in THF-isopropanol (10 mL) afforded **4c** as a white solid (0.25 g, 80%), mp. 215–217 °C; R_f_ (1:1, toluene/ethyl acetate) 0.64; ν_max_ (ATR) 466, 502, 535, 660, 824, 1156, 1210, 1222, 1357, 1421, 1498, 1534, 1574, 1613, 1630, 3278 cm^−1^; δ_H_ (500 MHz, DMSO-*d*_6_) 7.24 (t, *J* = 8.5 Hz, 2H), 7.38 (t, *J* = 8.5 Hz, 2H), 7.80–7.85 (m, 3H), 7.90 (t, *J* = 8.5 Hz, 2H), 8.15 (dd, *J* = 2.0 and 8.5 Hz, 1H), 8.55 (s, 1H), 8.78 (d, *J* = 2.0 Hz, 1H), 9.94 (s, 1H); δ_C_ (125 MHz, DMSO-*d*_6_) 115.5 (d, ^2^*J*_CF_ = 21.8 Hz), 115.6, 116.3 (d, ^2^*J*_CF_ = 20.9 Hz), 120.7, 125.2 (d, ^3^*J*_CF_ = 7.5 Hz), 128.8, 129.5 (d, ^3^*J*_CF_ = 8.5 Hz), 132.2, 135.6 (d, ^4^*J*_CF_ = 2.9 Hz), 136.0 (d, ^4^*J*_CF_ = 3.7 Hz), 137.4, 149.3, 154.9, 158.4, 159.1 (d, ^1^*J*_CF_ = 239.9 Hz), 162.6 (d, ^1^*J*_CF_ = 243.7 Hz); *m*/*z* 334 (100, M + H); HRMS (ES): found 334.1166. C_20_H_14_F_2_N_3_^+^ requires 334.1156.

*[6-(4-Fluorophenyl)quinazolin-4-yl]-3-chlorophenylamine* (**4d**). A mixture of **3d** (0.30 g, 0.89 mmol), PdCl_2_(PPh_3_)_2_ (0.033 g, 0.044 mmol), 4-fluorophenylboronic acid (0.15 g, 1.07 mmol) and K_2_CO_3_ (0.18 g, 1.33 mmol) in THF-isopropanol (10 mL) afforded **4d** as a white solid (0.19 g, 61%), mp. 207–209 °C; R_f_ (1:1, toluene/ethyl acetate) 0.71; ν_max_ (ATR) 510, 587, 683, 777, 827, 933, 1236, 1356, 1395, 1417, 1478, 1498, 1529, 1562, 1597, 2849, 2918, 3245 cm^−1^; δ_H_ (500 MHz, DMSO-*d*_6_) 7.18 (dd, *J* = 2.0 and 8.5 Hz, 1H), 7.36‒7.45 (m, 3H), 7.85 (d, *J* = 8.5 Hz, 1H), 7.86 (d, *J* = 8.5 Hz, 1H), 7.91 (t, *J* = 8.5 Hz, 2H), 8.08 (s, 1H), 8.18 (dd, *J* = 2.0 and 8.5 Hz, 1H), 8.65 (s, 1H), 8.79 (d, *J* = 2.5 Hz, 1H), 9.98 (s, 1H); δ_C_ (125 MHz, DMSO-*d*_6_) 115.4, 116.3 (d, ^2^*J*_CF_ = 20.7 Hz), 116.4, 116.5, 120.8, 125.0, 128.1, 128.2, 128.6, 128.8, 129.5 (d, ^3^*J*_CF_ = 8.5 Hz), 135.8 (d, ^4^*J*_CF_ = 2.8 Hz), 137.5, 149.1, 155.1, 156.3, 159.3, 162.6 (d, ^1^*J*_CF_ = 244.6 Hz); *m*/*z* 350 (100, M + H); HRMS (ES): found 350.0853. C_20_H_14_^35^ClFN_3_^+^ requires 350.0860.

*[6-(4-Fluorophenyl)quinazolin-4-yl]-2, 4-difluorophenylamine* (**4f**). A mixture of **3f** (0.30 g, 0.89 mmol), PdCl_2_(PPh_3_)_2_ (0.031 g, 0.044 mmol), 4-fluorophenylboronic acid (0.15 g, 1.07 mmol) and K_2_CO_3_ (0.18 g, 1.33 mmol) in THF-isopropanol (10 mL) afforded a white solid (0.24 g, 77%), mp. 228–231 °C; R_f_ (1:1, toluene/ethyl acetate) 0.60; ν_max_ (ATR) 470, 496, 513, 665, 784, 824, 965, 1141, 1241, 1325, 1401, 1499, 1578, 2852, 2922, 3159 cm^−1^; δ_H_ (500 MHz, DMSO-*d*_6_) 7.17 (td, *J* = 2.0 and 8.5 Hz, 1H), 7.35–7.43 (m, 3H), 7.56 (q, *J* = 8.0 Hz, 1H), 7.84 (d, *J* = 8.5 Hz, 1H), 7.91 (t, *J* = 8.0 Hz, 2H), 8.19 (dd, *J* = 2.0 and 8.5 Hz, 1H), 8.46 (s, 1H), 8.76 (d, *J* = 2.0 Hz, 1H), 9.96 (s, 1H); δ_C_ (125 MHz, DMSO-*d*_6_) 105.0 (t, ^2^*J*_CF_ = 26.5 Hz), 112.0 (d, ^2^*J*_CF_ = 21.8 Hz), 115.2, 116.4 (d, ^2^*J*_CF_ = 20.8 Hz), 120.7, 125.8, 128.5, 129.4 (d, ^3^*J*_CF_ = 7.6 Hz), 130.0 (d, ^4^*J*_CF_ = 2.8 Hz), 130.1, 132.5, 135.7, 137.7, 148.7 (d, ^4^*J*_CF_ = 2.8 Hz), 155.0, 157.5 (d, ^1^*J*_CF_ = 248.5 Hz), 159.4, 162.6 (d, ^1^*J*_CF_ = 243.6 Hz); *m*/*z* 352 (100, M + H); HRMS (ES): found 352.1074. C_20_H_13_F_3_N_3_^+^ requires 352.1062.

*[2-(4-Chlorophenyl)-6-(4-fluorophenyl)quinazolin-4-yl]-2-fluorophenylamine* (**4g**). A mixture of **3g** (0.30 g, 0.70 mmol), PdCl_2_(PPh_3_)_2_ (0.024 g, 0.035 mmol), 4-fluorophenylboronic acid (0.12 g, 0.84 mmol) and K_2_CO_3_ (0.14 g, 1.05 mmol) in THF-isopropanol (10 mL) afforded **4g** as a white solid (0.21 g, 68%), mp. 195–197 °C; R_f_ (toluene) 0.53; ν_max_ (ATR) 503, 516, 741, 815, 1013, 1086, 1231, 1420, 1454, 1494, 1514, 1538, 1559, 1597, 3045, 3446 cm^−1^; δ_H_ (500 MHz, DMSO-*d*_6_) 7.31‒7.42 (m, 5H), 7.49 (d, *J* = 8.5 Hz, 2H), 7.67 (td, *J* = 2.5 and 8.5 Hz, 1H), 7.90‒7.96 (m, 3H), 8.20 (dd, *J* = 2.5 and 8.5 Hz, 1H), 8.25 (d, *J* = 8.5 Hz, 2H), 8.82 (d, *J* = 2.0 Hz, 1H), 10.11 (s, 1H); δ_C_ (125 MHz, DMSO-*d*_6_) 114.4, 116.3 (d, ^2^*J*_CF_ = 20.9 Hz), 116.4 (d, ^2^*J*_CF_ = 19.9 Hz), 120.9, 124.9 (d, ^4^*J*_CF_ = 2.8 Hz), 126.5 (d, ^3^*J*_CF_ = 11.4 Hz), 127.8 (d, ^3^*J*_CF_ = 8.5 Hz), 128.4, 128.9, 129.1, 129.4 (d, ^3^*J*_CF_ = 8.5 Hz), 129.9, 132.4, 135.6, 135.9 (d, ^4^*J*_CF_ = 2.8 Hz), 137.2, 137.4, 150.1, 157.1 (d, ^1^*J*_CF_ = 245.6 Hz), 158.5, 159.2, 162.6 (d, ^1^*J*_CF_ = 243.7 Hz); *m*/*z* 444 (100, M + H); HRMS (ES): found 444.1068. C_26_H_17_^35^ClF_2_N_3_^+^ requires 444.1079.

*[2-(4-Chlorophenyl)-6-(4-fluorophenyl)quinazolin-4-yl]-3-fluorophenylamine* (**4h**). A mixture of **3h** (0.30 g, 0.70 mmol), PdCl_2_(PPh_3_)_2_ (0.024 g, 0.035 mmol), 4-fluorophenylboronic acid (0.12 g, 0.84 mmol) and K_2_CO_3_ (0.14 g, 1.05 mmol) in THF-isopropanol (10 mL) afforded **4h** as a white solid (0.22 g, 71%), mp. 226–228 °C; R_f_ (toluene) 0.38; ν_max_ (ATR) 431, 522, 670, 762, 820, 969, 1084, 1138, 1159, 1232, 1428, 1488, 1560, 1604, 2857, 2926, 3446 cm^−1^; δ_H_ (500 MHz, DMSO-*d*_6_) 7.00 (td, *J* = 2.0 and 8.5 Hz, 1H), 7.40 (t, *J* = 8.5 Hz, 2H), 7.49 (q, *J* = 8.0 Hz, 1H), 7.58 (d, *J* = 8.5 Hz, 2H), 7.75 (dd, *J* = 2.0 and 8.5 Hz, 1H), 7.88–7.97 (m, 4H), 8.20 (dd, *J* = 2.0 and 8.5 Hz, 1H), 8.42 (d, *J* = 8.5 Hz, 2H), 8.83 (d, *J* = 2.0 Hz, 1H), 10.15 (s, 1H); δ_C_ (125 MHz, DMSO-*d*_6_) 109.4 (d, ^2^*J*_CF_ = 25.6 Hz), 110.6 (d, ^2^*J*_CF_ = 20.9 Hz), 114.6, 116.3 (d, ^2^*J*_CF_ = 21.9 Hz), 118.3 (d, ^4^*J*_CF_ = 2.8 Hz), 120.9, 129.1, 129.2, 129.4, 129.6 (d, ^3^*J*_CF_ = 7.6 Hz), 129.9, 130.5 (d, ^3^*J*_CF_ = 9.5 Hz), 132.6, 135.7, 136.0 (d, ^4^*J*_CF_ = 2.8 Hz), 137.4, 141.4 (d, ^3^*J*_CF_ = 10.5 Hz), 150.2, 158.3, 158.4, 162.5 (d, ^1^*J*_CF_ = 239.9 Hz), 162.6 (d, ^1^*J*_CF_ = 243.6 Hz); *m*/*z* 444 (100, M + H); HRMS (ES): found 444.1081. C_26_H_17_^35^ClF_2_N_3_^+^ requires 444.1079.

*[2-(4-Chlorophenyl)-6-(4-fluorophenyl)quinazolin-4-yl]-4-fluorophenylamine* (**4i**). A mixture of **3i** (0.30 g, 0.70 mmol), PdCl_2_(PPh_3_)_2_ (0.024 g, 0.035 mmol), 4-fluorophenylboronic acid (0.12 g, 0.84 mmol) and K_2_CO_3_ (0.14 g, 1.05 mmol) in THF-isopropanol (10 mL) afforded **4i** as a white solid (0.23 g, 76%), mp. 221–222 °C; R_f_ (toluene) 0.31; ν_max_ (ATR) 435, 487, 506, 527, 822, 945, 1009, 1082, 1153, 1205, 1420, 1503, 1533, 1560, 3451 cm^−1^; δ_H_ (500 MHz, DMSO-*d*_6_) 7.31 (t, *J* = 8.5 Hz, 2H), 7.39 (t, *J* = 8.5 Hz, 2H), 7.55 (d, *J* = 8.5 Hz, 2H), 7.86–7.96 (m, 5H), 8.17 (dd, *J* = 2.0 and 8.5 Hz, 1H), 8.38 (d, *J* = 8.5 Hz, 2H), 8.80 (d, *J* = 2.0 Hz, 1H), 10.06 (s, 1H); δ_C_ (125 MHz, DMSO-*d*_6_) 114.5, 115.6 (d, ^2^*J*_CF_ = 21.7 Hz), 116.3 (d, ^2^*J*_CF_ = 21.7 Hz), 120.8, 124.9 (d, ^3^*J*_CF_ = 8.0 Hz), 128.9, 129.1, 129.4 (d, ^3^*J*_CF_ = 8.0 Hz), 130.0, 132.3, 135.5, 135.7 (d, ^4^*J*_CF_ = 2.8 Hz), 136.0 (d, ^4^*J*_CF_ = 3.7 Hz), 137.2, 137.5, 150.0, 158.4, 158.5, 158.9 (d, ^1^*J*_CF_ = 239.4 Hz), 162.6 (d, ^1^*J*_CF_ = 243.9 Hz); *m*/*z* 444 (100, M + H); HRMS (ES): found 444.1078. C_26_H_17_^35^ClF_2_N_3_^+^ requires 444.1079.

*[2-(4-Chlorophenyl)-6-(4-fluorophenyl)quinazolin-4-yl]-3-chlorophenylamine* (**4j**). A mixture of **3j** (0.30 g, 0.67 mmol), PdCl_2_(PPh_3_)_2_ (0.023 g, 0.034 mmol), 4-fluorophenylboronic acid (0.11 g, 0.80 mmol) and K_2_CO_3_ (0.14 g, 1.00 mmol) in THF-isopropanol (10 mL) afforded **4j** as a white solid (0.19 g, 61%), mp. 244–246 °C; R_f_ (toluene) 0.40; ν_max_ (ATR) 530, 673, 765, 818, 828, 1086, 1156, 1206, 1351, 1403, 1425, 1474, 1494, 1508, 1526, 1556, 1598, 3450 cm^−1^; δ_H_ (500 MHz, DMSO-*d*_6_) 7.23 (dd, *J* = 2.5 and 8.5 Hz, 1H), 7.39 (t, *J* = 8.5 Hz, 2H), 7.49 (t, *J* = 8.5 Hz, 1H), 7.57 (d, *J* = 8.5 Hz, 2H), 7.90‒7.95 (m, 4H), 8.13 (s, 1H), 8.18 (dd, *J* = 2.0 and 8.5 Hz, 1H), 8.41 (d, *J* = 8.5 Hz, 2H), 8.80 (d, *J* = 2.5 Hz, 1H), 10.09 (s, 1H); δ_C_ (125 MHz, DMSO-*d*_6_) 114.6, 116.3 (d, ^2^*J*_CF_ = 21.8 Hz), 120.8, 120.9, 122.2, 123.8, 129.0, 129.2, 129.5 (d, ^3^*J*_CF_ = 8.5 Hz), 129.9, 130.6, 133.6, 133.2, 135.7, 135.9 (d, ^4^*J*_CF_ = 2.8 Hz), 137.4, 137.5, 141.1, 150.2, 158.2, 158.3, 162.6 (d, ^1^*J*_CF_ = 243.7 Hz); *m*/*z* 460 (100, M + H); HRMS (ES): found 460.0780. C_26_H_17_^35^Cl_2_FN_3_^+^ requires 460.0784.

*[2-(4-Chlorophenyl)-6-(4-fluorophenyl)quinazolin-4-yl]-2,4-difluorophenylamine* (**4l**). A mixture of **3l** (0.30 g, 0.67 mmol), PdCl_2_(PPh_3_)_2_ (0.023 g, 0.034 mmol), 4-fluorophenylboronic acid (0.11 g, 0.80 mmol) and K_2_CO_3_ (0.14 g, 1.00 mmol) in THF-isopropanol (10 mL) afforded **4l** as a white solid (0.21 g, 69%), mp. 210–212 °C; R_f_ (toluene) 0.48; ν_max_ (ATR) 425, 515, 737, 817, 838, 964, 1012, 1089, 1161, 1234, 1415, 1502, 1530, 1558, 1585, 3450 cm^−1^; δ_H_ (500 MHz, DMSO-*d*_6_) 7.22 (td, *J* = 2.5 and 8.0 Hz, 1H), 7.36–7.43 (m, 3H), 7.45–7.52 (m, 3H), 7.68 (q, *J* = 8.0 Hz, 1H), 7.91–7.96 (m, 3H), 8.21 (dd, *J* = 2.0 and 8.5 Hz, 1H), 8.25 (d, *J* = 8.5 Hz, 2H), 8.80 (d, *J* = 2.0 Hz, 1H), 10.09 (s, 1H); δ_C_ (125 MHz, DMSO-*d*_6_) 105.0 (t, ^2^*J*_CF_ = 25.2 Hz), 112.0 (d, ^2^*J*_CF_ = 21.7 Hz), 114.3, 116.3 (d, ^2^*J*_CF_ = 21.7 Hz), 120.9, 123.0, 123.3, 128.9, 129.4 (d, ^3^*J*_CF_ = 8.0 Hz), 129.5, 129.7 (d, ^4^*J*_CF_ = 2.8 Hz), 129.8, 132.4, 135.6, 135.8 (d, ^4^*J*_CF_ = 3.5 Hz), 137.2, 137.3, 150.0, 157.2 (d, ^1^*J*_CF_ = 248.5 Hz), 158.4, 158.5, 162.6 (d, ^1^*J*_CF_ = 243.9 Hz); *m*/*z* 462 (100, M + H); HRMS (ES): found 462.0982. C_26_H_16_^35^ClF_3_N_3_^+^ requires 462.0985.

### 3.6. Reaction Sequence for the Preparation of ***4e*** and ***4k*** from ***1a*** and ***1b***, Respectively

#### 3.6.1. Typical Procedure for the Preparation of **5a** and **5b**

*6-(4-Fluorophenyl)quinazolin-4(3H)-one* (**5a**). A mixture of 6-bromoquinazolin-4(3*H*)-one **1a** (0.50 g, 2.22 mmol) PdCl_2_(PPh_3_)_2_ (0.078 g, 0.11 mmol) and K_2_CO_3_ (0.46 g, 3.33 mmol) in dioxane-water (20 mL) in a two-necked round-bottomed flask was purged with argon gas for 30 minutes. 4-Fluorophenylboronic acid (0.37 g, 2.66 mmol) in dioxane (10 mL) was added via a syringe. The reaction mixture was stirred at 70 °C for 3 h until the starting material was completely consumed (TLC monitoring). After cooling the mixture, the product was extracted with chloroform and the combined organic layers were washed with water, dried over Na_2_SO_4_, filtered, and evaporated under reduced pressure. The residue was purified by recrystallization from acetonitrile to afford **5a** as a white solid (0.43 g, 81%), mp. 228–230 °C; ν_max_ (ATR) 496, 698, 746, 821, 923, 1160, 1239, 1275, 1333, 1384, 1477, 1603, 1687, 2673, 3046 cm^−1^; δ_H_ (500 MHz, DMSO-*d*_6_) 7.31 (t, *J* = 9.0 Hz, 2H), 7.73 (d, *J* = 8.5 Hz, 1H), 7.79 (t, *J* = 8.5 Hz, 2H), 8.09 (dd, *J* = 2.0 and 8.5 Hz, 1H), 8.10 (s, 1H), 8.28 (d, *J* = 2.0 Hz, 1H), 12.30 (br.s, 1H); δ_C_ (125 MHz, DMSO-*d*_6_) 116.4 (d, ^2^*J*_CF_ = 21.7 Hz), 123.2, 123.6, 127.8, 129.4 (d, ^3^*J*_CF_ = 7.9 Hz), 133.4 (d, ^4^*J*_CF_ = 2.8 Hz), 134.0, 135.5, 135.6, 138.0, 146.4, 162.6 (d, ^1^*J*_CF_ = 240.5 Hz); *m*/*z* 241 (100, M + H); HRMS (ES): found 241.0780. C_14_H_10_FN_2_O^+^ requires 241.0777.

*2-(4-Chlorophenyl)-6-(4-fluorophenyl)quinazolin-4(3H)-one* (**5b**). A mixture of 6-bromo-2-(4-chlorophenyl)quinazolin-4(3*H*)-one **1b** (0.5 g, 1.49 mmol) PdCl_2_(PPh_3_)_2_ (0.052 g, 0.074 mmol), 4-fluorophenylboronic acid (0.25 g, 1.78 mmol) and K_2_CO_3_ (0.31 g, 2.23 mmol) in dioxane-water (30 mL) afforded **5b** as a white solid (0.46 g, 88%), mp. 151–153 °C; ν_max_ (ATR) 501, 539, 693, 719, 752, 825, 1118, 1180, 1190, 1308, 1436, 1483, 1603, 1674, 2926, 3056 cm^−1^; δ_H_ (500 MHz, DMSO-*d*_6_) 7.32 (t, *J* = 8.0 Hz, 2H), 7.61 (d, *J* = 8.5 Hz, 2H), 7.79–7.85 (m, 3H), 8.13 (dd, *J* = 2.0 and 8.5 Hz, 1H), 8.20 (d, *J* = 8.5 Hz, 2H), 8.33 (d, *J* = 2.5 Hz, 1H), 12.67 (br.s, 1H); δ_C_ (125 MHz, DMSO-*d*_6_) 116.4 (d, ^2^*J*_CF_ = 21.8 Hz), 122.6, 128.3, 128.5, 128.9, 129.1, 129.2 (d, ^3^*J*_CF_ = 8.6 Hz), 130.1, 131.8, 132.4 (d, ^4^*J*_CF_ = 2.8 Hz), 136.4, 144.8, 146.3, 148.9, 161.2, 162.5 (d, ^1^*J*_CF_ = 243.5 Hz); *m*/*z* 351 (100, M + H); HRMS (ES): found 351.0702. C_20_H_13_^35^ClFN_2_O requires 351.0700.

#### 3.6.2. Typical Procedure for the Preparation of **6a** and **6b**

*4-Chloro-6-(4-fluorophenyl)quinazoline* (**6a**). Triethylamine (4 mL) was added dropwise at room temperature to a stirred mixture of **5a** (0.40 g, 1.66 mmol) and phosphoryl chloride (10 mL) in a round-bottomed flask. The reaction mixture was stirred under reflux for 5 h and then allowed to cool to room temperature. Ice-cold water was added to the mixture and the product was extracted with chloroform. The combined organic layers were washed with an aqueous solution of NaHCO_3_, dried over Na_2_SO_4_, filtered, and evaporated under reduced pressure. The crude product was recrystallized from ethanol to afford **6a** as a white solid (0.33 g, 77%), mp. 154–156 °C (EtOH); ν_max_ (ATR) 478, 513, 523, 697, 825, 991, 1096, 1146, 1225, 1335, 1484, 1511, 1545, 1565, 1600 cm^−1^; δ_H_ (500 MHz, DMSO-*d*_6_) 7.16 (t, *J* = 8.5 Hz, 2H), 7.47 (d, *J* = 8.5 Hz, 1H), 7.80 (t, *J* = 8.5 Hz, 2H), 8.12 (dd, *J* = 2.0 and 8.5 Hz, 1H), 8.33 (s, 1H), 8.37 (d, *J* = 2.0 Hz, 1H); δ_C_ (125 MHz, DMSO-*d*_6_) 116.3 (d, ^2^*J*_CF_ = 21.8 Hz), 122.8, 123.1, 128.8, 129.2 (d, ^3^*J*_CF_ = 8.3 Hz), 132.3, 134.1 (d, ^4^*J*_CF_ = 3.2 Hz), 136.7, 149.8, 157.7, 159.0, 162.4 (d, ^1^*J*_CF_ = 240.2 Hz); *m*/*z* 258 (100, M + H); HRMS (ES): found 257.8652. C_14_H_8_^35^ClFN_2_ requires 257.8652.

*4-Chloro-2-(4-chlorophenyl)-6-(4-fluorophenyl)quinazoline* (**6b**). A mixture of **5b** (0.40 g, 1.14 mmol), trimethylamine (4 mL) and phosphoryl chloride (10 mL) afforded **6b** as a white solid (0.35 g, 83%), mp. 216–218 °C (EtOH); ν_max_ (ATR) 514, 553, 700, 735, 778, 821, 988, 1010, 1088, 1162, 1237, 1360, 1401, 1431, 1492, 1514, 1554, 1604 cm^−1^; δ_H_ (500 MHz, DMSO-*d*_6_) 7.31 (t, *J* = 8.5 Hz, 2H), 7.61 (d, *J* = 8.5 Hz, 2H), 7.79–7.84 (m, 3H), 8.13 (dd, *J* = 2.0 and 8.5 Hz, 1H), 8.19 (d, *J* = 8.5 Hz, 2H), 8.31 (d, *J* = 2.0 Hz, 1H); δ_C_ (125 MHz, DMSO-*d*_6_) 116.2 (d, ^2^*J*_CF_ = 20.4 Hz), 121.3, 123.4, 125.6, 128.7, 128.9, 129.1 (d, ^3^*J*_CF_ = 8.3 Hz), 130.2, 131.9, 134.6 (d, ^4^*J*_CF_ = 3.2 Hz), 138.4, 139.8, 148.6, 157.8, 158.2, 162.6 (d, ^1^*J*_CF_ = 245.3 Hz); *m*/*z* 368 (100, M + H); HRMS (ES): found 368.0435. C_20_H_12_^35^Cl_2_FN_2_ requires 368.0273.

#### 3.6.3. Typical Procedure for the Preparation of **4e** and **4k**

*[6-(4-Fluorophenyl)quinazolin-4-yl]-4-bromophenylamine* (**4e**). A mixture of **6a** (0.30 g, 1.16 mmol), 4-bromaniline (0.22 g, 1.28 mmol) and concentrated HCl (0.01 g, 0.27 mmol) in 3:1 THF-isopropanol (v/v, 10 mL) in a round-bottomed flask was stirred under reflux for 5 h. The mixture was then allowed to cool to room temperature and quenched with ice-cold water. The product was extracted with ethyl acetate and the combined organic layers were washed with an aqueous solution of NaHCO_3_, dried over anhydrous MgSO_4_, filtered, and evaporated under reduced pressure. The crude product was purified by column chromatography on silica gel to afford **4e** as a white solid (0.37 g, 81%), mp. 227–229 °C; R_f_ (1:1, toluene/ethyl acetate) 0.69; ν_max_ (ATR) 477, 507, 662, 823, 925, 1006, 1160, 1232, 1358, 1416, 1487, 1498, 1523, 1560, 1603, 3280 cm^−1^; δ_H_ (500 MHz, DMSO-*d*_6_) 7.38 (t, *J* = 8.5 Hz, 2H), 7.58 (d, *J* = 8.5 Hz, 2H), 7.84 (d, *J* = 8.5 Hz, 1H), 7.85 (d, *J* = 8.5 Hz, 2H), 7.90 (t, *J* = 8.5 Hz, 2H), 8.16 (dd, *J* = 2.0 and 8.5 Hz, 1H), 8.59 (s, 1H), 8.78 (d, *J* = 8.0 Hz, 1H), 9.96 (s, 1H); δ_C_ (125 MHz, DMSO-*d*_6_) 115.8, 115.8, 116.3 (d, ^2^*J*_CF_ = 21.7 Hz), 120.8, 124.7, 128.9, 129.6 (d, ^3^*J*_CF_ = 8.0 Hz), 131.7, 132.3, 136.0 (d, ^4^*J*_CF_ = 2.8 Hz), 137.5, 138.9, 149.4, 154.8, 158.1, 162.6 (d, ^1^*J*_CF_ = 242.8 Hz); *m*/*z* 394 (100, M + H); HRMS (ES): found 394.0356. C_20_H_14_^79^BrFN_3_^+^ requires 394.0356.

*[2-(4-Chlorophenyl)-6-(4-fluorophenyl)quinazolin-4-yl]-4-bromophenylamine* (**4k**). A mixture of **6b** (0.30 g, 0.81 mmol), 4-bromoaniline (0.15 g, 0.89 mmol) and concentrated HCl (0.01 g, 0.27 mmol) in 3:1 THF-isopropanol (v/v, 10 mL) afforded **4k** as a white solid (0.32 g, 79%), mp. 257–260 °C; R_f_ (toluene) 0.36; ν_max_ (ATR) 499, 521, 666, 740, 817, 1012, 1090, 1161, 1230, 1354, 1398, 1417, 1489, 1511, 1525, 1557, 1595, 3428 cm^-1^; δ_H_ (500 MHz, DMSO-*d*_6_) 7.39 (t, *J* = 8.5 Hz, 2H), 7.58 (d, *J* = 8.5 Hz, 2H), 7.65 (d, *J* = 8.5 Hz, 2H), 7.87–7.96 (m, 5H), 8.20 (dd, *J* = 2.5 and 8.5 Hz, 1H), 8.40 (d, *J* = 9.0 Hz, 2H), 8.82 (d, *J* = 2.0 Hz, 1H), 10.19 (s, 1H); δ_C_ (125 MHz, DMSO-*d*_6_) 114.5, 116.2, 116.3 (d, ^2^*J*_CF_ = 20.7 Hz), 120.9, 124.9, 129.1, 129.2, 129.3, 129.5 (d, ^3^*J*_CF_ = 8.5 Hz), 130.1, 131.8, 131.9, 132.7, 135.8, 135.9 (d, ^4^*J*_CF_ = 2.8 Hz), 137.5, 138.8, 158.3, 158.4, 162.7 (d, ^1^*J*_CF_ = 243.7 Hz); *m*/*z* 504 (100, M + H); HRMS (ES): found 504.0278. C_26_H_17_^79^Br^35^ClFN_3_^+^ requires 504.0282.

### 3.7. Materials and Methods for the In Vitro Cytotoxicity Assay

The 4-anilinoquinazolines **3a**–**l** and **4a**–**l** were evaluated for growth inhibitory activity against the MCF-7 and HeLa cell lines against Gefitnib as a reference standard using the MTT assay developed by Mosmann [22] with slight modification as described in our previous investigation [9]. The percentage of cell viability was calculated using the following formula:

(1) $$\%\mathrm{Cell}\;\mathrm{viability}=\frac{\mathrm{Mean}\;\mathrm{Absorbance}\;\mathrm{of}\;\mathrm{sample}\times 100}{\mathrm{Mean}\;\mathrm{Absorbance}\;\mathrm{of}\;\mathrm{control}}$$

The LC_50_ values (the lethal concentration at which 50% of the cells are killed) were calculated as the concentration of the test sample that resulted in 50% reduction of absorbance compared to untreated cells. The intensity of the MTT formazan produced by living metabolically active cells is directly proportional to the number of live cells present.

### 3.8. Inhibition of EGFR-TK

The inhibitory activities of compounds **3g**, **3l**, **4l** and Gefitinib towards EGFR-TK were tested using enzyme-linked immunosorbent assay (ELISA) technique with purified Epidermal Growth Factor Receptor (Sigma-Aldrich, Bradford, UK). The procedure was carried out according to the manufacturer’s protocol. Ninety-six-well plates were pre-coated with 100 µL of a 4:1 poly(Glu,Tyr) solution (5 mg/mL) at 37 °C overnight. The enzyme reaction was conducted in freshly prepared kinase reaction buffer (25 mmol/L HEPES, pH 7.4, 5 mmol/L MgCl_2_, 2 mmol/L MnCl_2_, 100 µmol/L Na_3_VO_4_ and 1 mmol/L dithiothreitol). Ten microlitres of the test samples at six different concentrations (0.005–0.2 µM) and 20 µL of dilute solution of purified recombinant EGFR tyrosine kinase proteins were added to each reaction well. The kinase reaction was initiated by the addition of 50 µL of a solution of adenosine-5′-triphosphate, disodium salt (5 mmol/L). DMSO and EGFR (without any test sample) were also included in this experiment as negative and positive controls, respectively. The plates were then incubated at 37 °C for 1 h and washed three times with phosphate-buffered saline (PBS). Phosphorylated proteins were probed with monoclonal anti-phosphotyrosine antibody produced in mouse (Sigma-Aldrich, St. Louis, MO, USA). After another 1 h incubation at 37 °C, the plates were again washed three times with PBS, followed by the addition 100 µL of freshly prepared peroxidase substrate solution. After incubation in the dark for extra 10 min, the reaction was terminated by adding 100 µL of 2 M H_2_SO_4_ solution. The plates were analysed using the Thermo Varioskan Flash Spectral Scanning Multimode Reader (Thermo Fisher Scientific, Waltham, MA, USA) at 492 nm. The assay was performed in triplicate. The EGFR-TK inhibition percentage was determined as follows:

(2) $$\%\;\mathrm{Inhibition}=\frac{\left({\mathrm{OD}}_{\mathrm{samples}}-{\mathrm{OD}}_{\mathrm{positive}}\right)}{\left({\mathrm{OD}}_{\mathrm{negative}}-{\mathrm{OD}}_{\mathrm{positive}}\right)}$$

where OD is the optical density. 

The half-maximal inhibitory concentration value (LC_50_) was obtained from the curves of percentage inhibition.

### 3.9. Molecular Docking Studies

#### 3.9.1. Protein Structure

The starting structure of EFGR-TK was obtained from RCSB PDB (PDB id: 1M17) [23] and all water molecules and heteroatoms were removed. The polar hydrogen atoms, Kollman–Amber united atom charges and solvation parameters were then added using AutoDockTools [24]. 

#### 3.9.2. Ligand Structure

The starting coordinates of the ligand for control docking (erlotinib) was obtained from the heteroatoms in the EFGR-TK crystal (PDB id: 1M17) and the coordinates for Gefitinib and compounds **3g**, **3l** and **4l** were generated using ChemDraw Professional 15.0 (PerkinElmer Informatics, Waltham, MA, USA). Gefitinib and compounds **3g**, **3l** and **4l** were minimised with Chem3D module in ChemOffice Professional 15.0 (PerkinElmer Informatics). All polar hydrogen atoms for the ligands were left intact. The Gasteiger chargers and torsional angles of the ligands were assigned with AutoDockTools [24]. Standard protocol from AutoDock4.2 [24] for hydrated docking was applied where water molecules were added randomly around the ligands. 

#### 3.9.3. Molecular Docking Simulation

Grid maps were centred at the ligand binding site in EGFR-TK crystal (PDB id: 1M17) with 90 × 90 × 90 points with 0.375 Å spacing. Lamarckian genetic algorithm of 1,000,000 energy evaluations per run and a maximum number of 27,000 generation was applied. The number of individuals in population was 350 and the crossover rate was 0.8. The docked conformations were clustered with root mean square of 2.0 Å from 200 docking runs.

## 4. Conclusions

In summary, a series of novel 4-(halogenophenylamino)-6-bromoquinazolines and their 6-(4-fluorophenyl)–substituted derivatives was designed, synthesised, and evaluated for cytotoxicity in vitro. Most of the synthesised compounds exhibited inhibitory activity against MCF-7 and HeLa cells. SAR based on these in vitro cytotoxicity results revealed that the presence of a 4-fluorophenyl- or 2,4-difluorophenylaniline is more desirable for cytotoxicity against both cell lines. A combination of 2-(4-chlorophenyl) and 6-(4-fluorophenyl) substituents favours activity against HeLa cells more so than the MCF-5 cell line. Compound **3l** not only demonstrated strong anti-proliferation activities against the two cancer cell lines, but also showed significant inhibitory activity (LC_50_ = 37.66 nM) towards EGFR, comparable to that of the medicinally important EGFR inhibitor Gefitinib (LC_50_ = 31.44 nM). Molecular docking (in silico) studies suggested that the title compounds bind nicely to the region of EGFR, with comparable binding affinity with Gefitinib. The observed increased in vitro cytotoxicity and inhibitory activity against EGFR-TK due to the presence of a 2-(4-halogenophenyl) substituent encourages further elaboration of this position to lead to derivatives with anticancer properties.

## Figures and Tables

**Table 1:** Percentage yields of **3a**–**l**, **4a**–**d** & **4f** and **4g**–**j** & **4l**.

Substrate	R	Ar	%Yield of 3	%Yield of 4
**2a**	H	2-FC_6_H_4_-	80 (**3a**)	64 (**4a**)
**2a**	H	3-FC_6_H_4_-	76 (**3b**)	72 (**4b**)
**2a**	H	4-FC_6_H_4_-	85 (**3c**)	80 (**4c**)
**2a**	H	3-ClC_6_H_4_-	79 (**3d**)	61 (**4d**)
**2a**	H	4-BrC_6_H_4_-	77 (**3e**)	-(**4e**) ^a^
**2a**	H	2,4-diFC_6_H_3_-	79 (**3f**)	77 (**4f**)
**2b**	4-ClC_6_H_4_-	2-FC_6_H_4_-	82 (**3g**)	68 (**4g**)
**2b**	4-ClC_6_H_4_-	3-FC_6_H_4_-	88 (**3h**)	71 (**4h**)
**2b**	4-ClC_6_H_4_-	4-FC_6_H_4_-	84 (**3i**)	75 (**4i**)
**2b** **2b** **2b**	4-ClC_6_H_4_- 4-ClC_6_H_4_- 4-ClC_6_H_4_-	3-ClC_6_H_4_- 4-BrC_6_H_4_- 2,4-diFC_6_H_3_-	87 (**3j**) 83 (**3k**) 93 (**3l**)	59 (**4j**) -(**4k**) ^a^ 69 (**4l**)

^a^ Compounds **4e** and **4k** were prepared following an independent route, as described in Scheme 2.

**Table 2:** Percentage yields of **5a**,**b**; **6a**,**b** and **4e**,**f**.

Substrate	R	5a,b	6a,b	4e,f
**1a**	H	81 (**5a**)	77 (**6a**)	81 (**4a**)
**1b**	4-ClC_6_H_4_-	88 (**5b**)	88 (**6b**)	59 (**4k**)

**Table 3:**
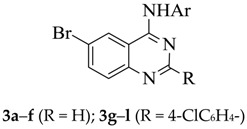
Cytotoxic effects of **3a**–**l** against MCF-7 and HeLa cell lines. The results are presented as LC_50_ (μM) ± standard deviation (SD) from three individual experiments.

Compound	R	Ar	LC_50_ (μM) ± SD	
			MCF-7	HeLa
**3a**	H	2-FC_6_H_4_-	1.60 ± 0.07	7.78 ±0.11
**3b**	H	3-FC_6_H_4_-	2.67 ± 0.88	3.95 ± 0.44
**3c**	H	4-FC_6_H_4_-	3.52 ± 0.78	3.24 ± 0.18
**3d**	H	3-ClC_6_H_4_-	4.26 ± 1.45	6.99 ± 0.14
**3e**	H	4-BrC_6_H_4_-	8.85 ± 1.22	11.05 ± 2.38
**3f**	H	2,4-diFC_6_H_3_-	1.54 ± 0.59	5.68 ± 1.04
**3g**	4-ClC_6_H_4_-	2-FC_6_H_4_-	0.14 ± 0.02	0.57 ± 0.11
**3h**	4-ClC_6_H_4_-	3-FC_6_H_4_-	0.73 ± 0.66	1.45 ± 0.39
**3i**	4-ClC_6_H_4_-	4-FC_6_H_4_-	1.44 ± 0.41	4.97 ± 0.03
**3j**	4-ClC_6_H_4_-	3-ClC_6_H_4_-	0.99 ± 0.18	2.77 ± 0.15
**3k**	4-ClC_6_H_4_-	4-BrC_6_H_4_-	2.03 ± 0.03	3.76 ± 0.62
**3l**	4-ClC_6_H_4_-	2,4-diFC_6_H_3_-	0.14 ± 0.04	0.18 ± 0.02
**Gefitinib**			0.51 ± 0.11	2.37± 1.21

**Table 4:**
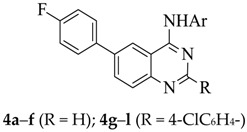
Cytotoxic effects of **4a**–**l** against MCF-7 and HeLa cell lines.

Compound	R	Ar	LC_50_ (μM) ± SD	
			MCF-7	HeLa
**4a**	H	2-FC_6_H_4_-	1.60 ± 0.19	1.60 ± 0.26
**4b**	H	3-FC_6_H_4_-	0.86 ± 0.08	1.24 ± 0.40
**4c**	H	4-FC_6_H_4_-	2.55 ± 0.81	0.55 ± 0.47
**4d**	H	3-ClC_6_H_4_-	7.64 ± 1.88	2.15 ± 0.47
**4e**	H	4-BrC_6_H_4_-	5.65 ± 1.96	3.18 ± 0.69
**4f**	H	2,4-diFC_6_H_3_-	2.09 ± 1.14	2.20 ± 1.78
**4g**	4-ClC_6_H_4_-	2-FC_6_H_4_-	1.32 ± 0.68	0.73 ± 0.34
**4h**	4-ClC_6_H_4_-	3-FC_6_H_4_-	2.41 ± 1.11	0.87 ± 0.17
**4i**	4-ClC_6_H_4_-	4-FC_6_H_4_-	2.70 ± 1.47	1.39 ± 0.32
**4j**	4-ClC_6_H_4_-	3-ClC_6_H_4_-	3.09 ± 0.83	5.23 ± 2.32
**4k**	4-ClC_6_H_4_-	4-BrC_6_H_4_-	0.90 ± 0.64	0.45 ± 0.29
**4l**	4-ClC_6_H_4_-	2,4-diFC_6_H_3_-	0.56 ± 0.21	0.47 ± 0.21
**Gefitinib**			0.51 ± 0.11	2.37± 1.21

**Table 5:** Calculated binding free energy and estimated inhibition constant (K*_i_*) of Erlotinib (as control), Gefitinib, and compounds **3g**, **3l**, and **4l** with EGFR-TK.

Ligand	Hb Distance (Å)	Hb Interacting Atoms	FEB (kcal/mol)	Ic, K*_i_* (nM)
Control (Erlotinib)	2.2	Met769 O–Erl H_N2_	−8.71	414.12
Gefitinib	1.9	Thr766 O_G1_–Gef H_N_	−10.74	13.34
**3g**	2.1	Asp831 O_D2_–3g H_N_	−9.14	200.91
**3l**	2.1	Asp831 O_D2_–3l H_N_	−8.83	337.36
**4l**	-	-	−10.46	21.60

Hb: Hydrogen bond; FEB: Free energy of binding; Ic: Estimated inhibition constant.

## Supplementary Materials

The following are available online at www.mdpi.com/1424-8247/10/4/87/s1.
