# Supplementary material for: Synthesis and In Vitro Cytotoxicity of the 4-(Halogenoanilino)-6-bromoquinazolines and Their 6-(4-Fluorophenyl) Substituted Derivatives as Potential Inhibitors of Epidermal Growth Factor Receptor Tyrosine Kinase
Source: Pharmaceuticals (Basel). 2017 Nov 20;10(4):87. doi: 10.3390/ph10040087 (PMC5748644; doi:10.3390/ph10040087)
Supplement: Supplementary file 1 [file pharmaceuticals-10-00087-s001.pdf]

Supplementary Materials: Synthesis and In Vitro Cytotoxicity of the 4-(Halogenoanilino)-6-bromoquinazolines and Their 6-(4-Fluorophenyl) Substituted Derivatives as Potential Inhibitors of Epidermal Growth Factor Receptor Tyrosine Kinase

Malose J. Mphahlele, Hugues K.Paumo and Yee Siew Choong

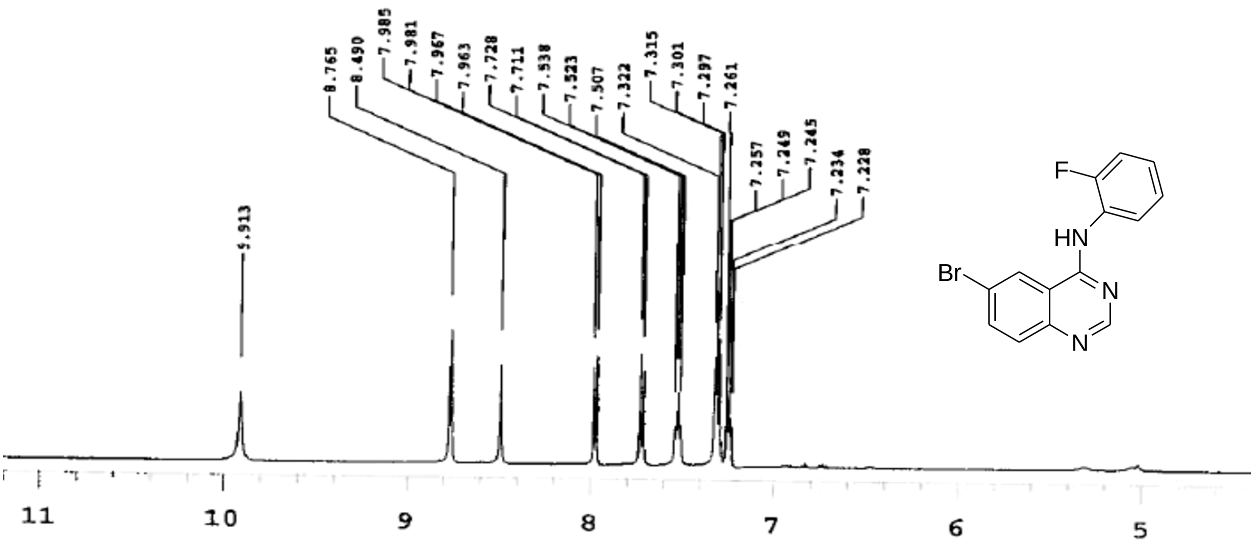

Figure 1. ^{1}H NMR spectrum of 3a in DMSO-d_6 at 500 MHz.

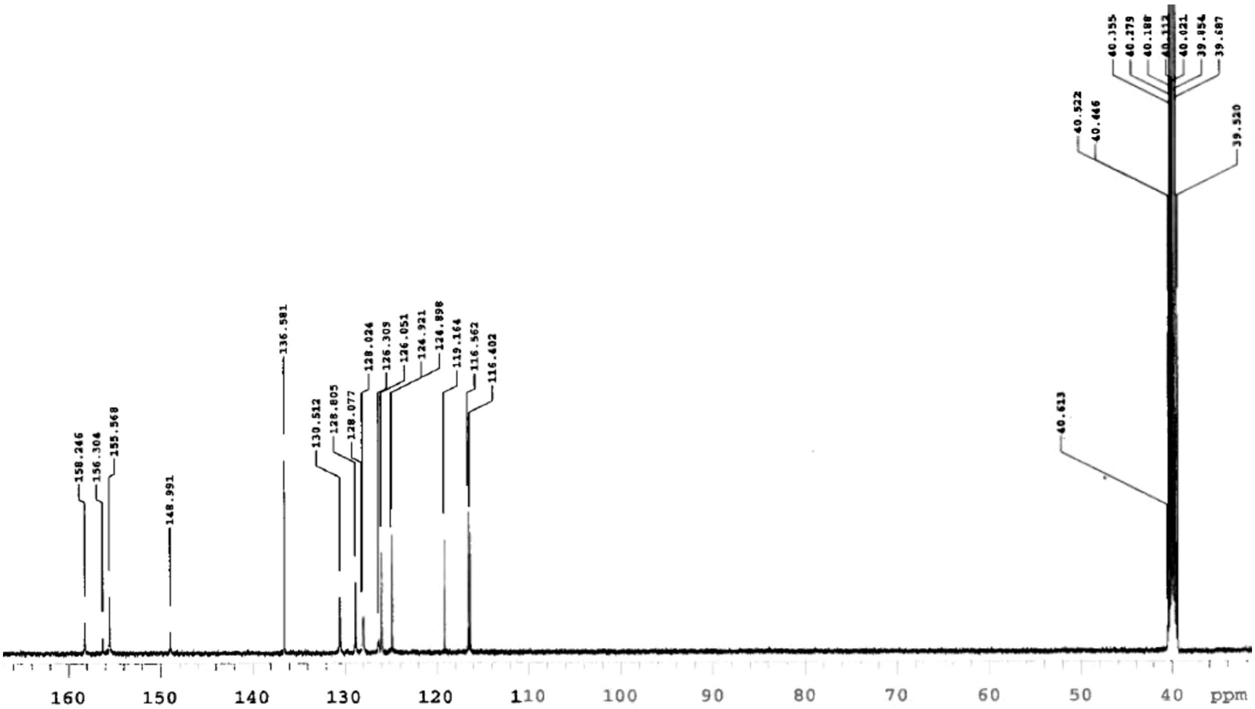

Figure 2. ^{13}C NMR spectrum of 3a in DMSO-d_6 at 125 MHz.

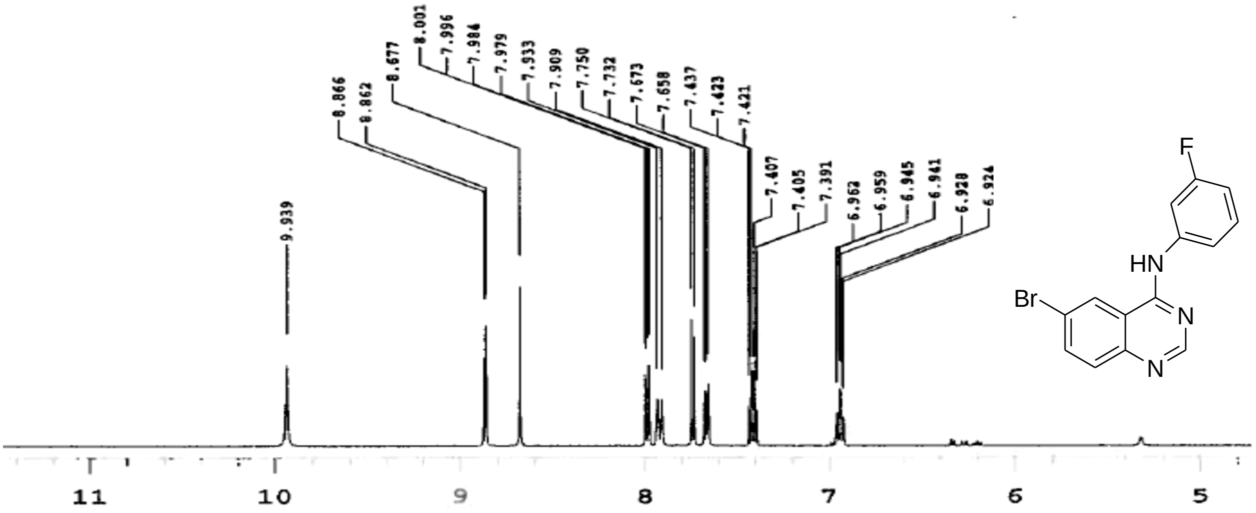

Figure 3. ^{1}H NMR spectrum of 3b in DMSO-d_6 at 500 MHz.

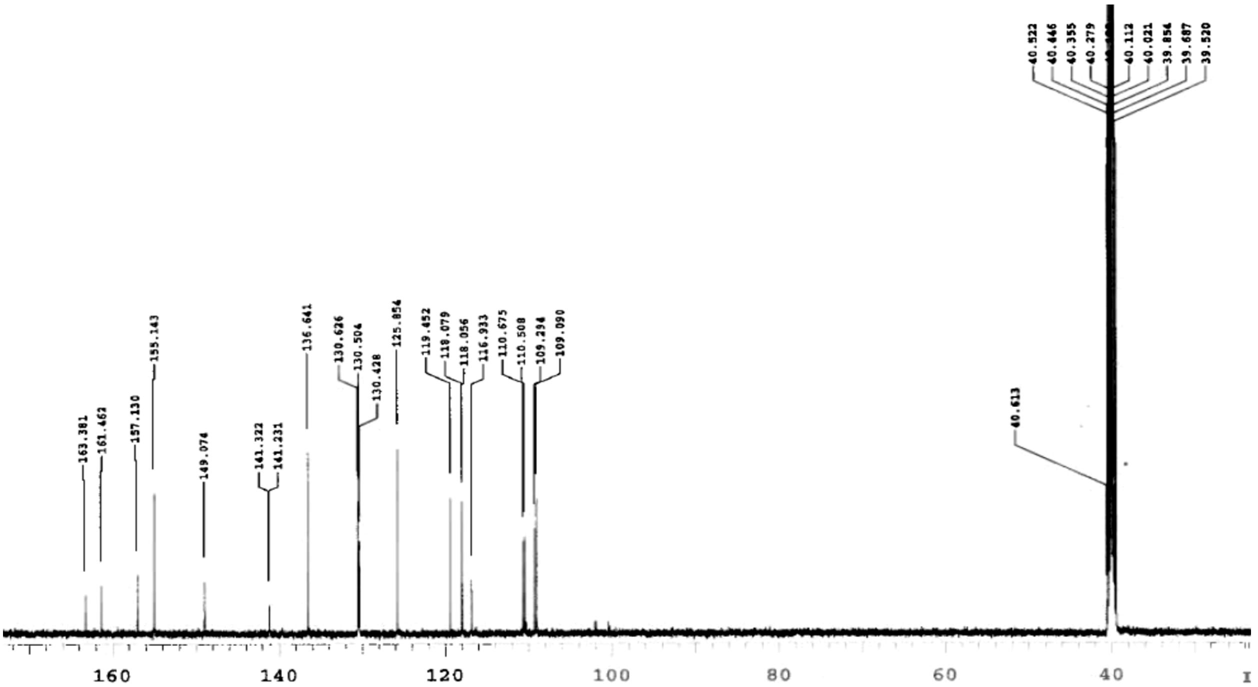

Figure 4. ^{13}C NMR spectrum of 3b in DMSO-d_6 at 125 MHz.

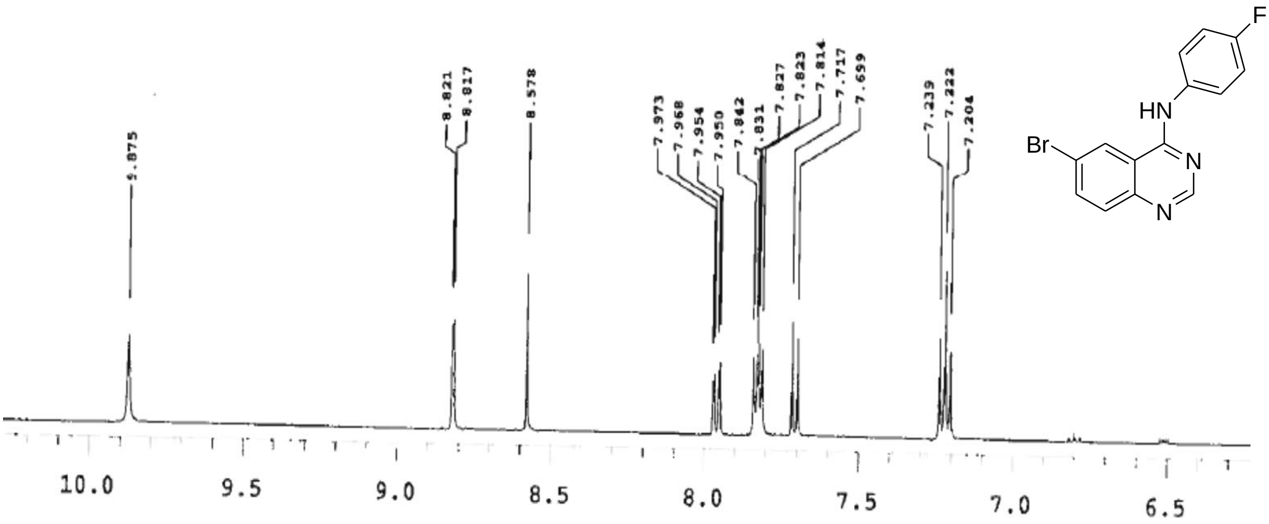

Figure 5. ^{1}H NMR spectrum of 3c in DMSO-d_6 at 500 MHz.

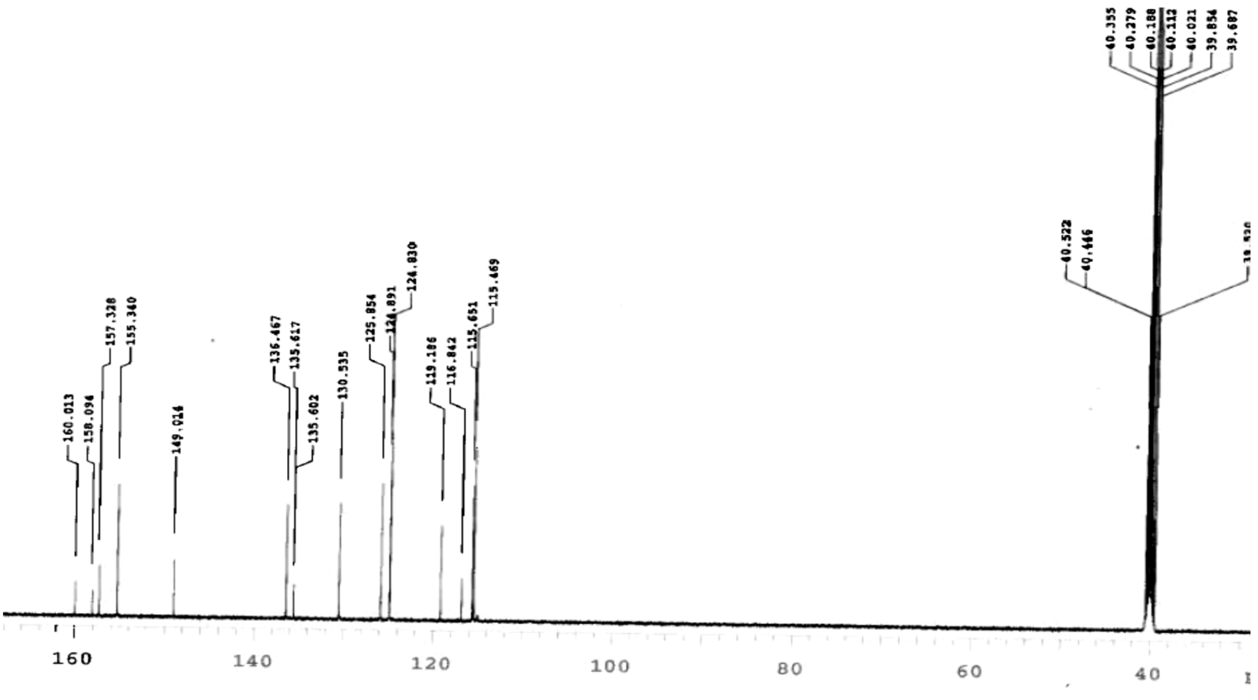

Figure 6. ^{13}C NMR spectrum of 3c in DMSO-d_6 at 125 MHz.

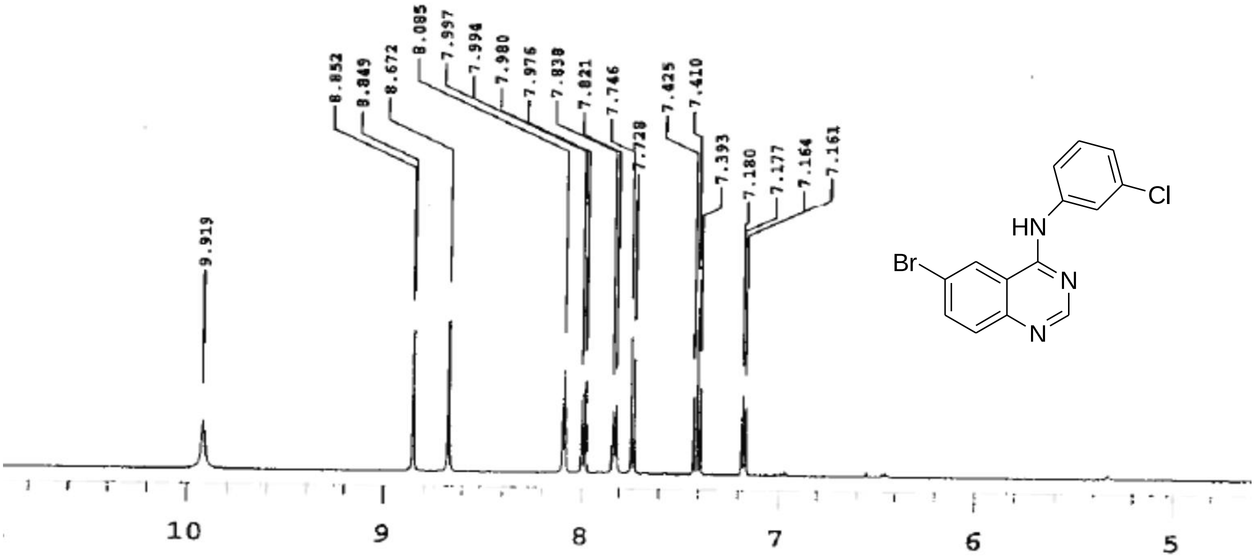

Figure 7. ^{1}H NMR spectrum of 3d in DMSO-d_6 at 500 MHz.

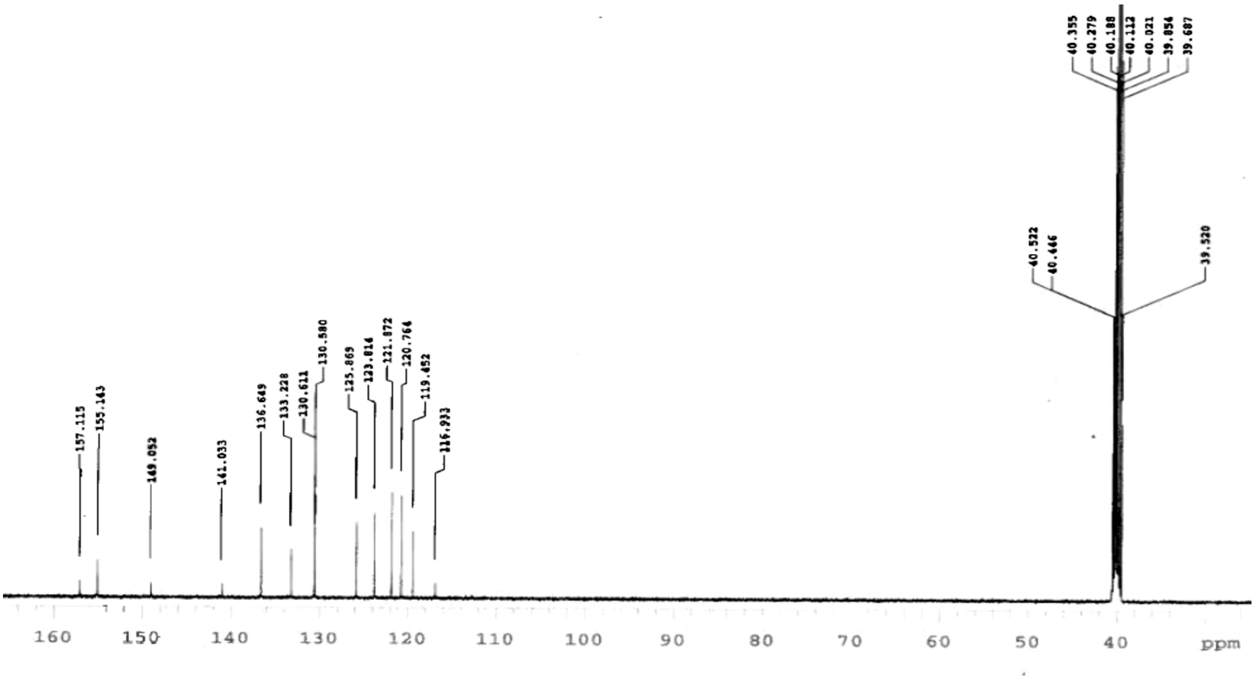

Figure 8. ^{13}C NMR spectrum of 3d in DMSO-d_6 at 125 MHz.

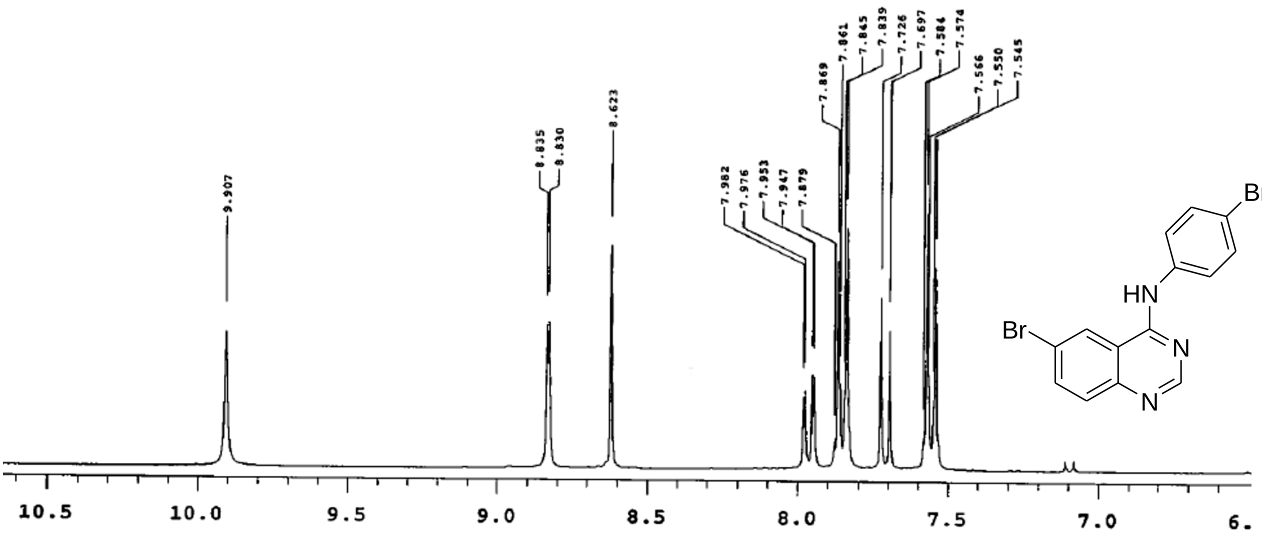

Figure 9. ^{1}H NMR spectrum of 3e in DMSO-d_6 at 500 MHz.

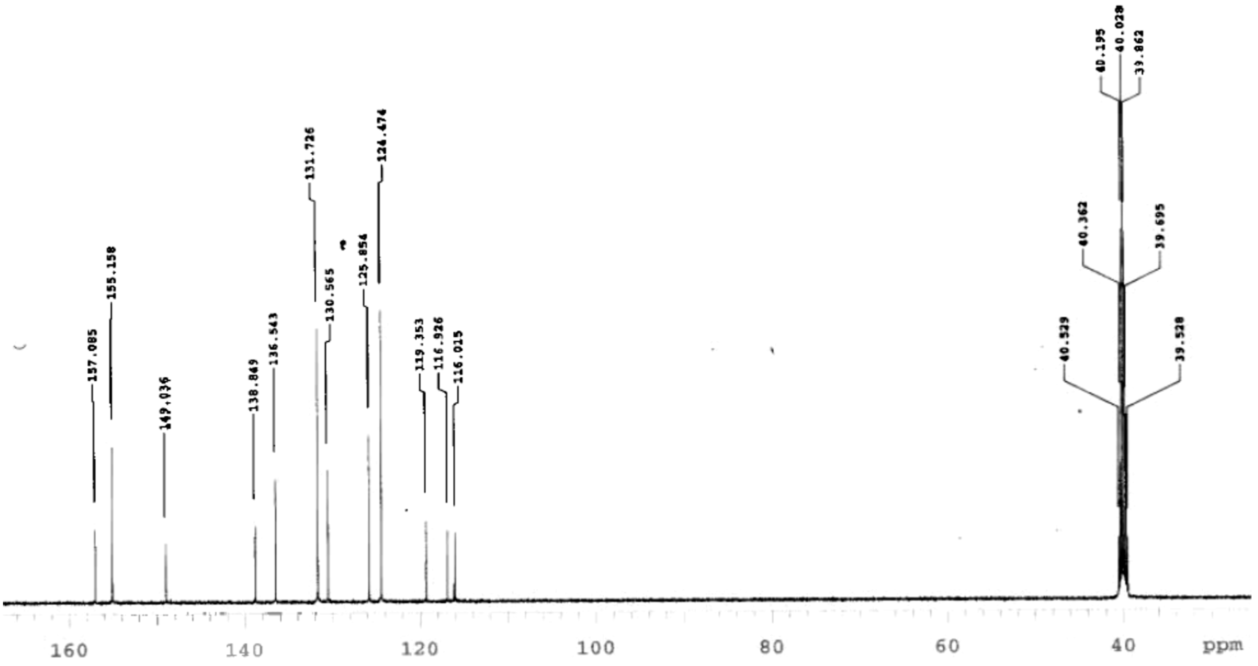

Figure 10. ^{13}C NMR spectrum of 3e in DMSO-d_6 at 125 MHz.

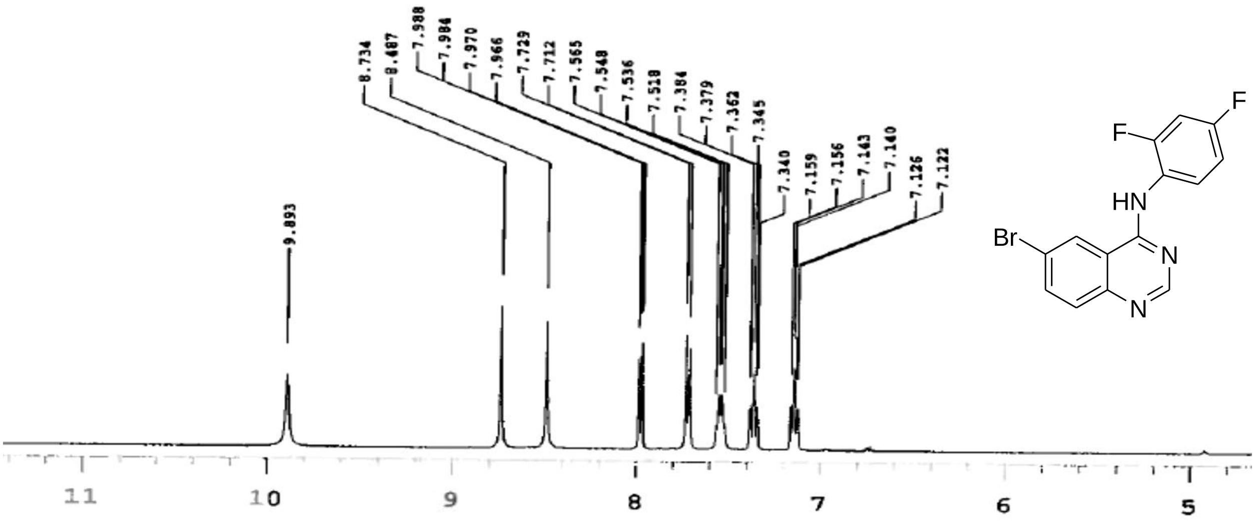

Figure 11. ^{1}H NMR spectrum of 3f in DMSO-d_6 at 500 MHz.

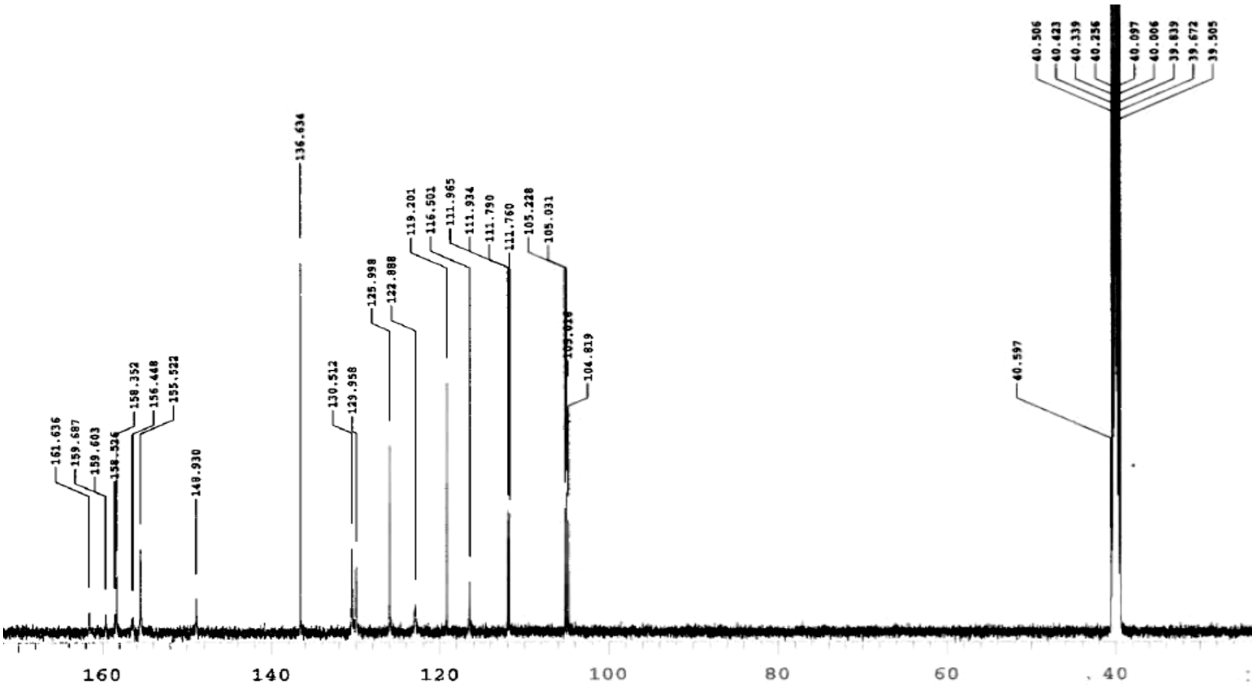

Figure 12. ^{13}C NMR spectrum of 3f in DMSO-d_6 at 125 MHz.

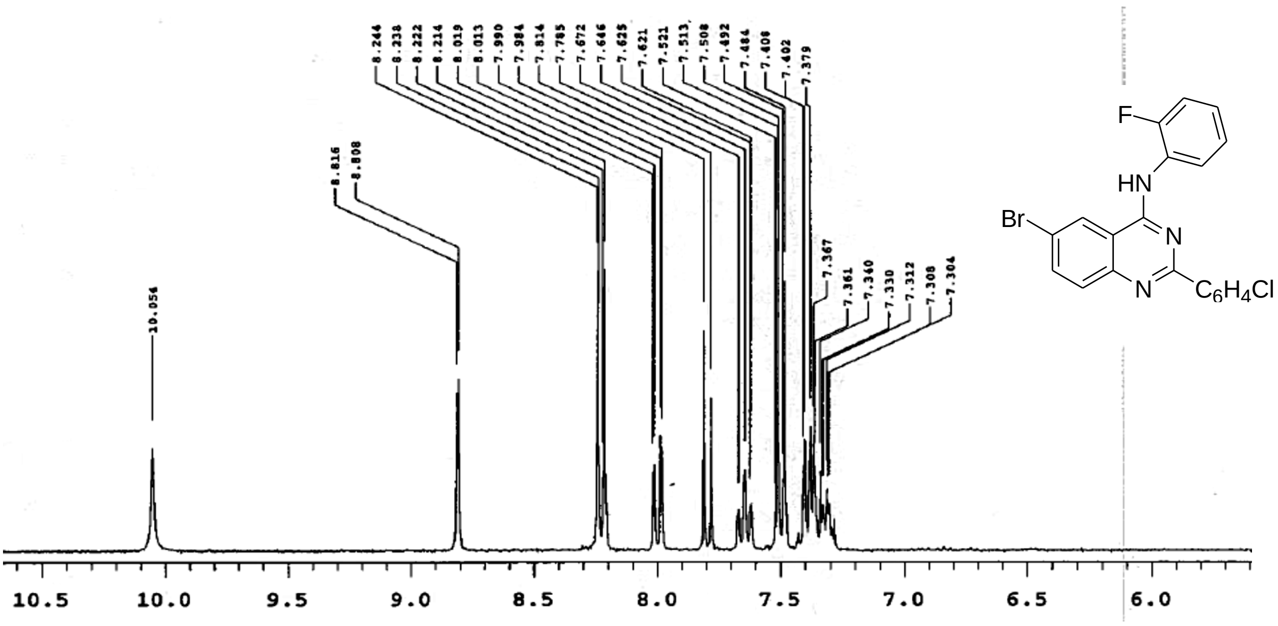

Figure 13. ^1H NMR spectrum of 3g in DMSO-d_6 at 500 MHz.

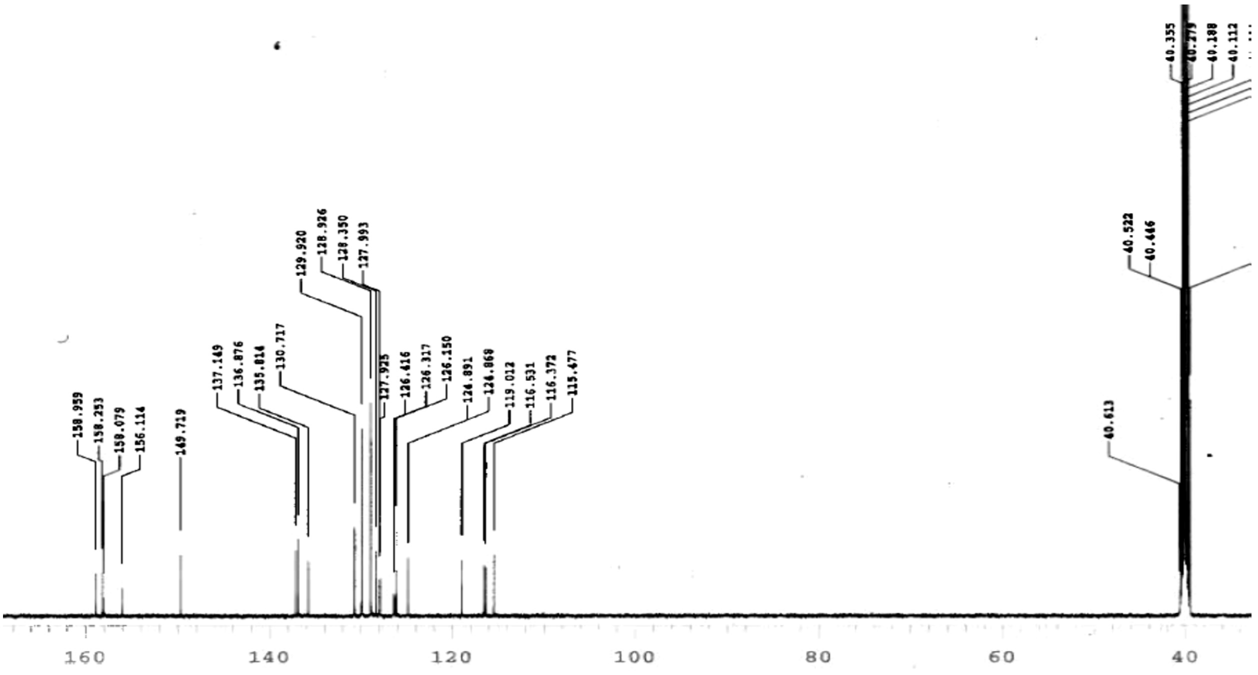

Figure 14. ^{13}C NMR spectrum of 3g in DMSO-d_6 at 125 MHz.

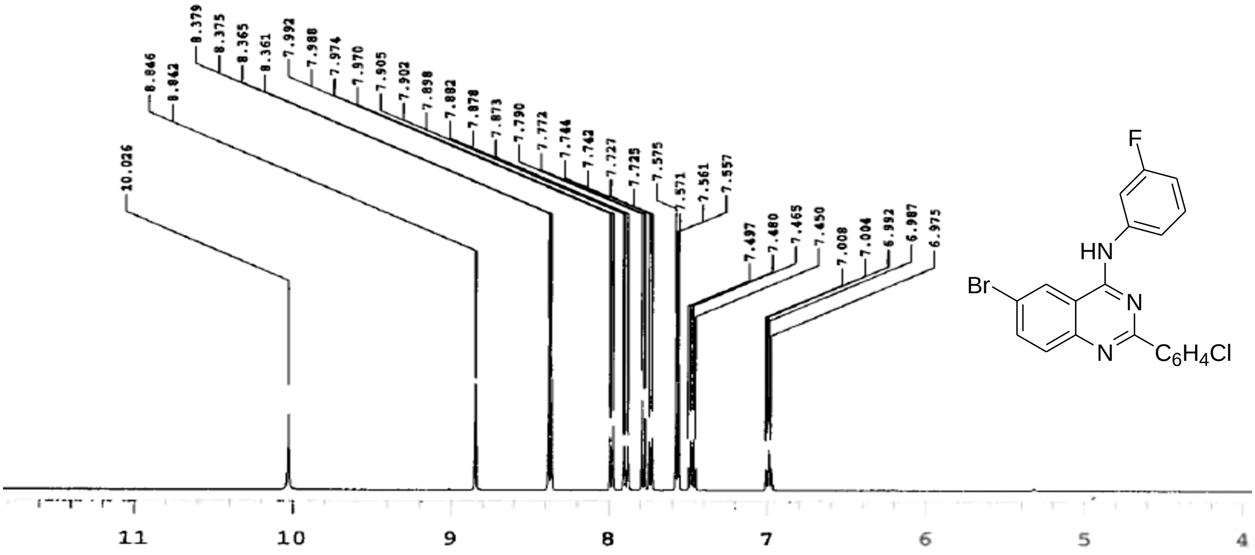

Figure 15. ^{1}H NMR spectrum of 3h in DMSO-d_6 at 500 MHz.

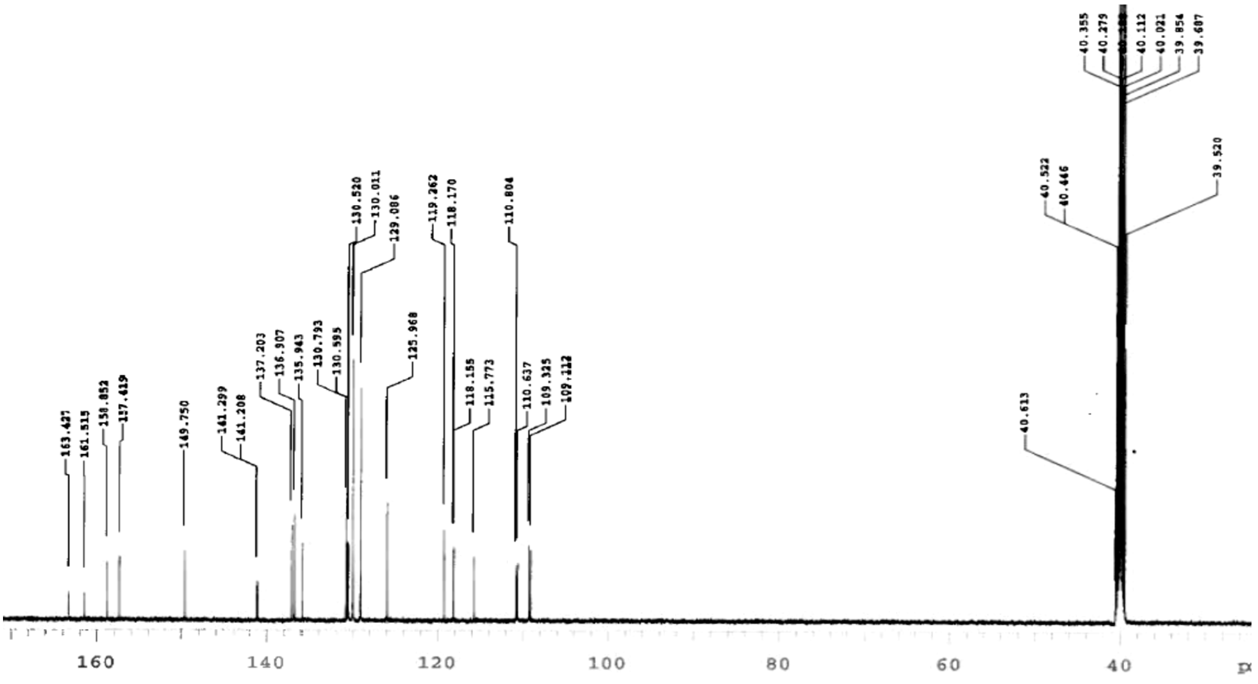

Figure 16. ^{13}C NMR spectrum of 3h in DMSO-d_6 at 125 MHz.

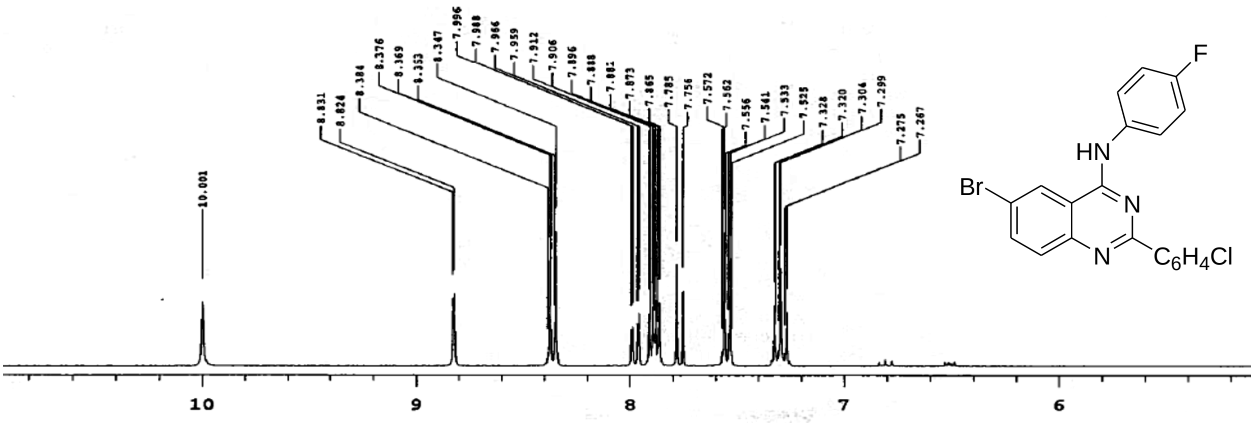

Figure 17. ^{1}H NMR spectrum of 3i in DMSO-d_6 at 500 MHz.

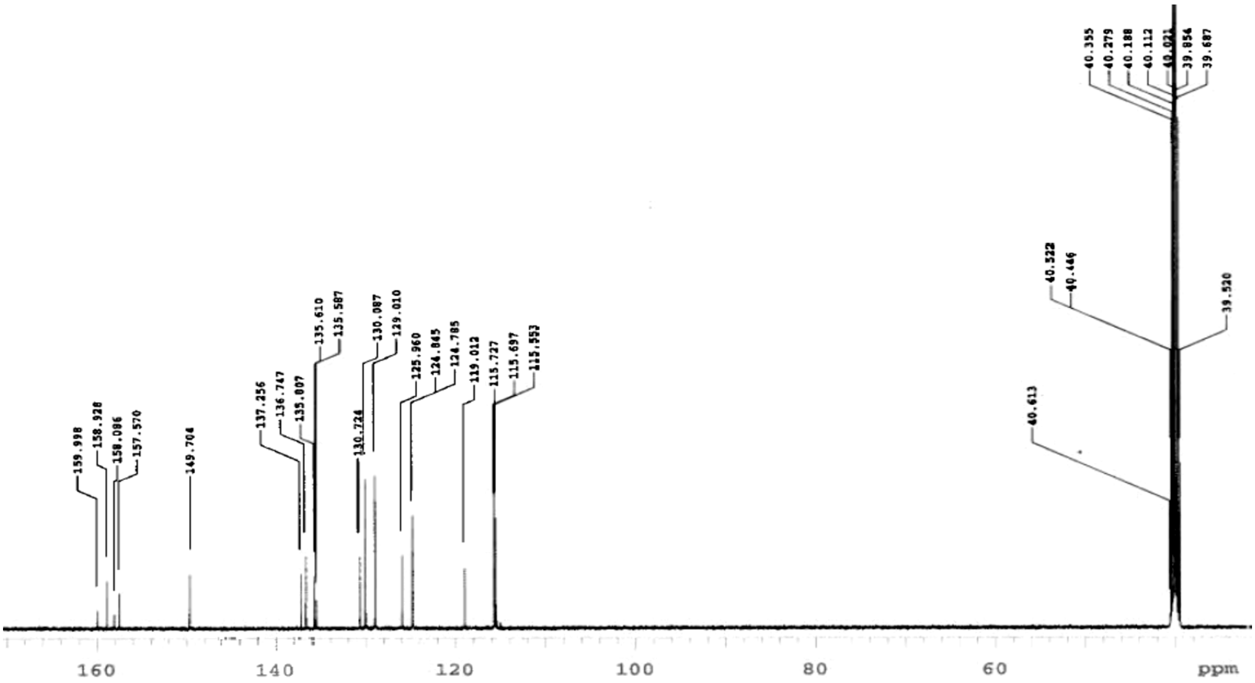

Figure 18. ^{13}C NMR spectrum of 3i in DMSO-d_6 at 125 MHz.

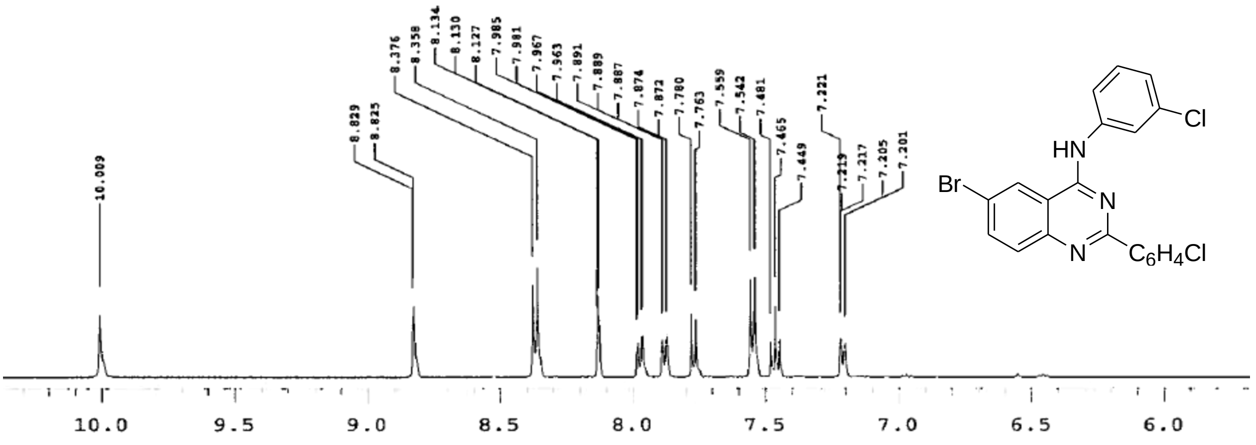

Figure 19. ^{1}H NMR spectrum of 3j in DMSO-d_6 at 500 MHz.

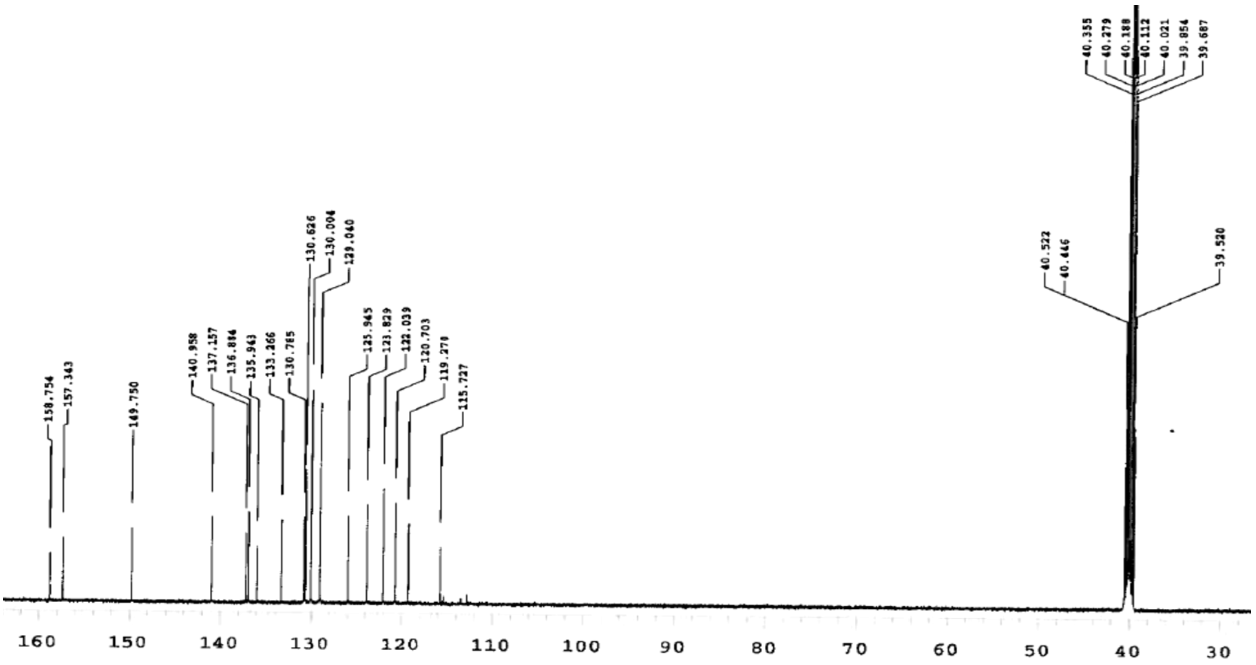

Figure 20. ^{13}C NMR spectrum of 3j in DMSO-d_6 at 125 MHz.

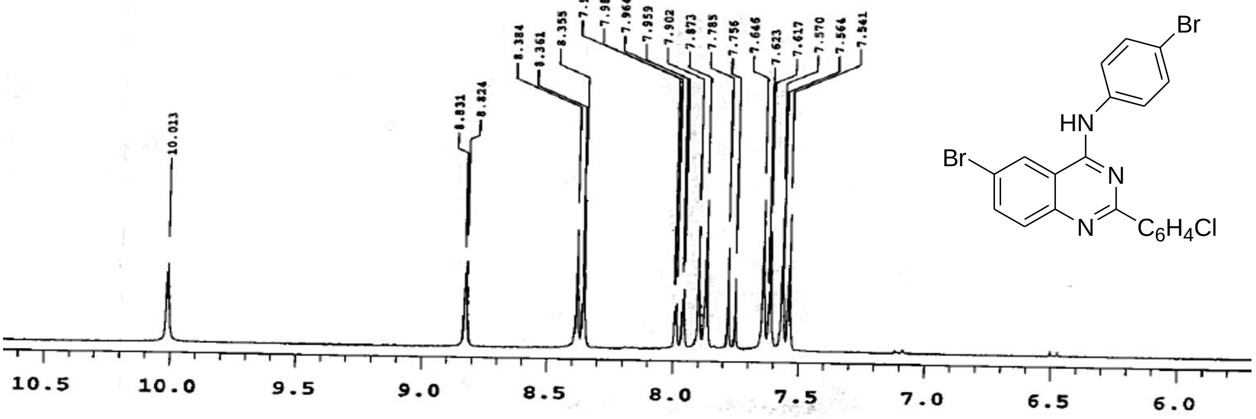

Figure 21. ^{1}H NMR spectrum of 3k in DMSO-d_6 at 500 MHz.

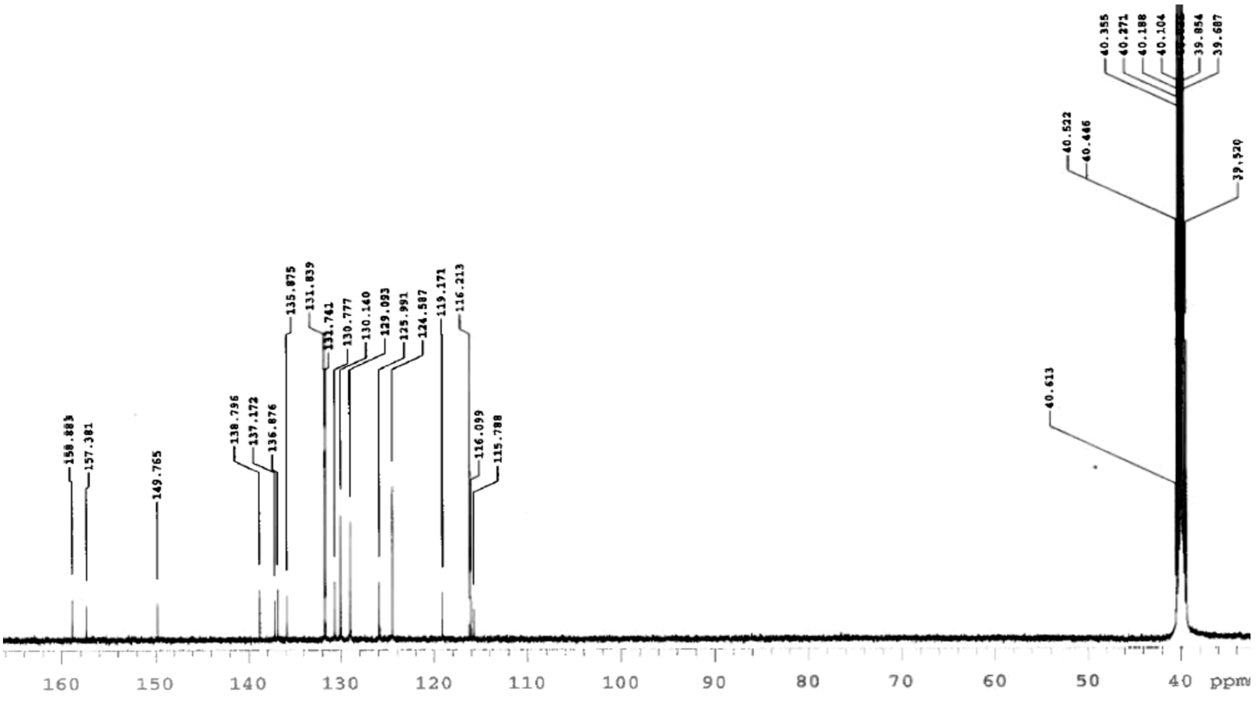

Figure 22. ^{13}C NMR spectrum of 3k in DMSO-d_6 at 125 MHz.

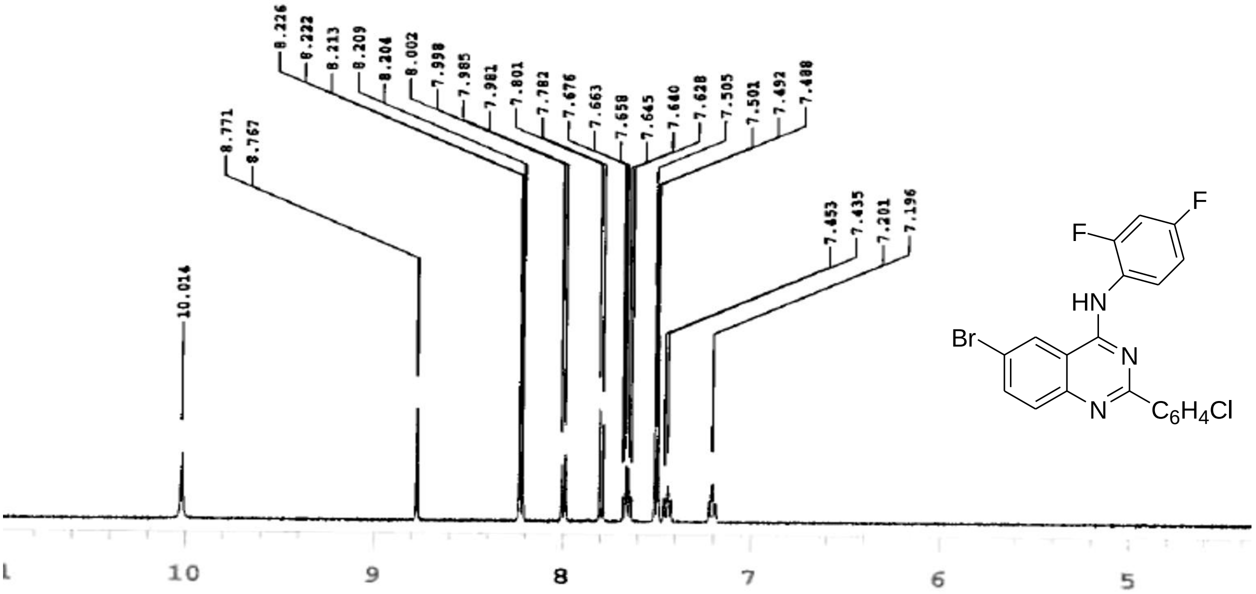

Figure 23. ^{1}H NMR spectrum of 3l in DMSO-d_6 at 500 MHz.

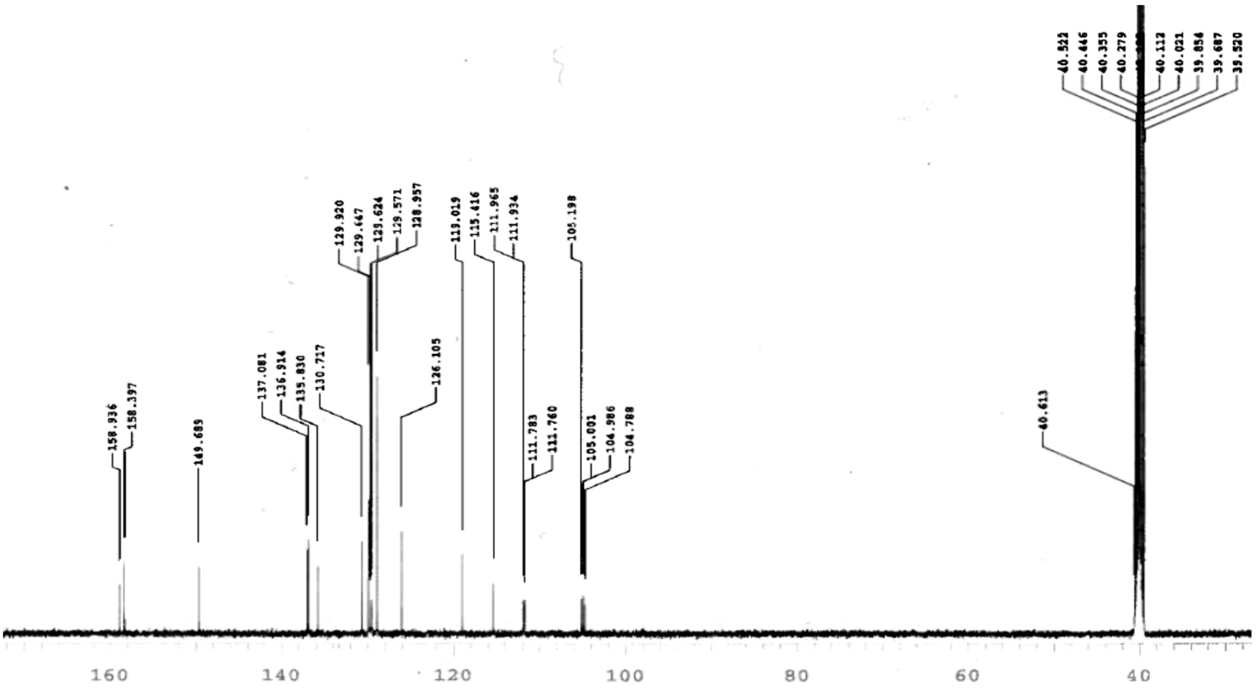

Figure 24. ^{13}C NMR spectrum of 3l in DMSO-d_6 at 125 MHz.

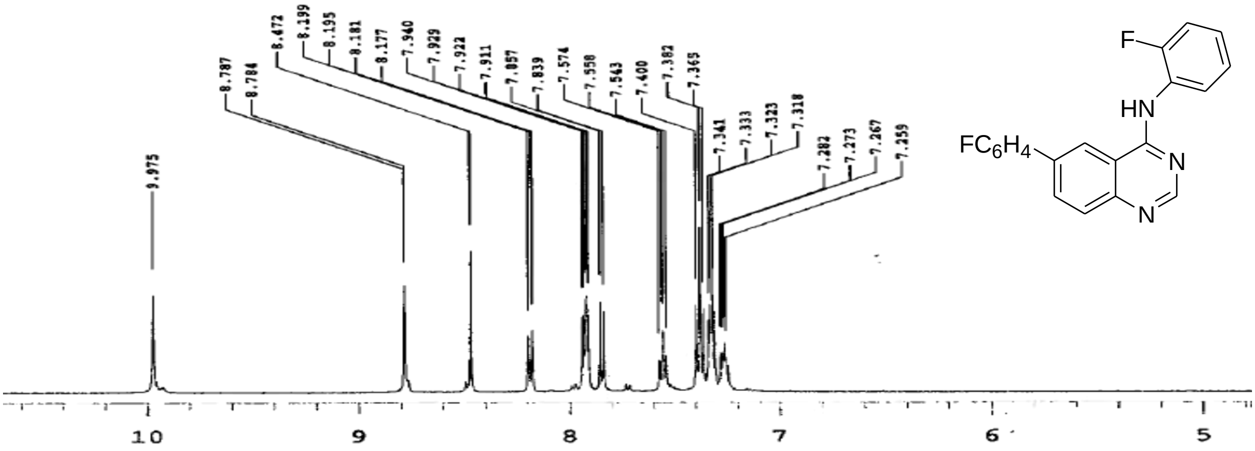

Figure 25. ^{1}H NMR spectrum of 4a in DMSO-d_6 at 500 MHz.

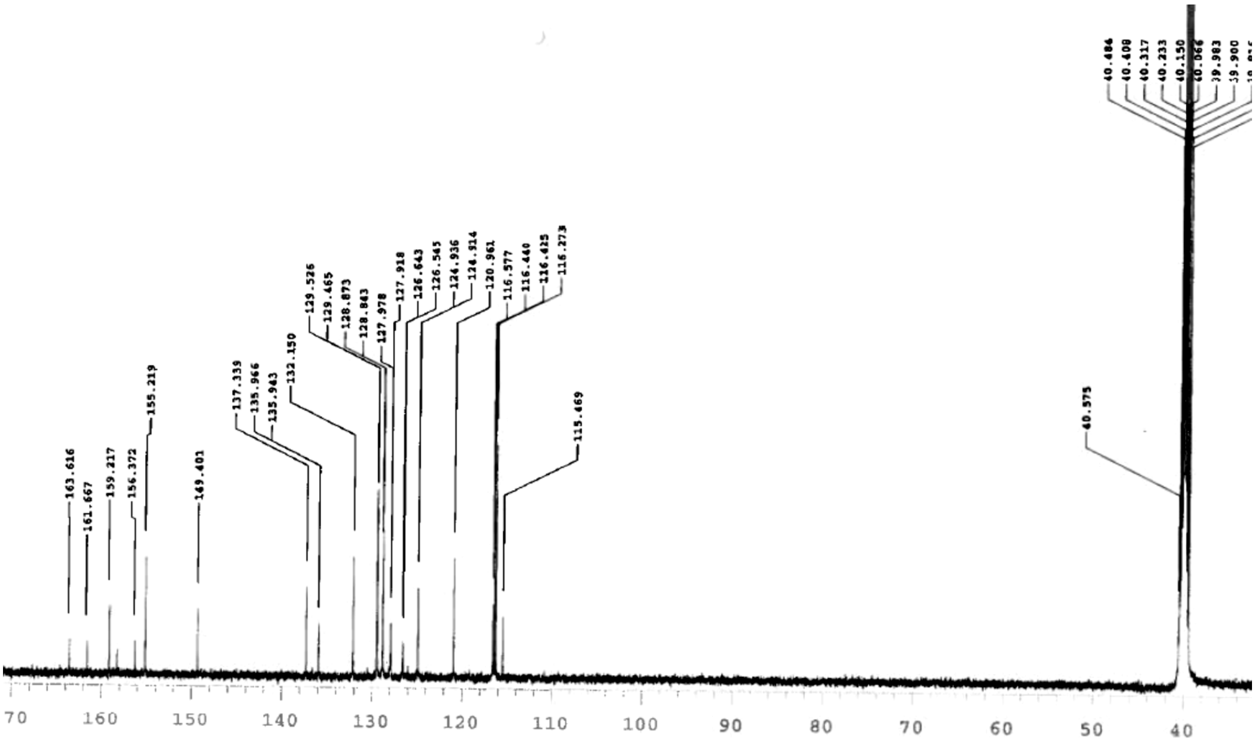

Figure 26. ^{13}C NMR spectrum of 4a in DMSO-d_6 at 125 MHz.

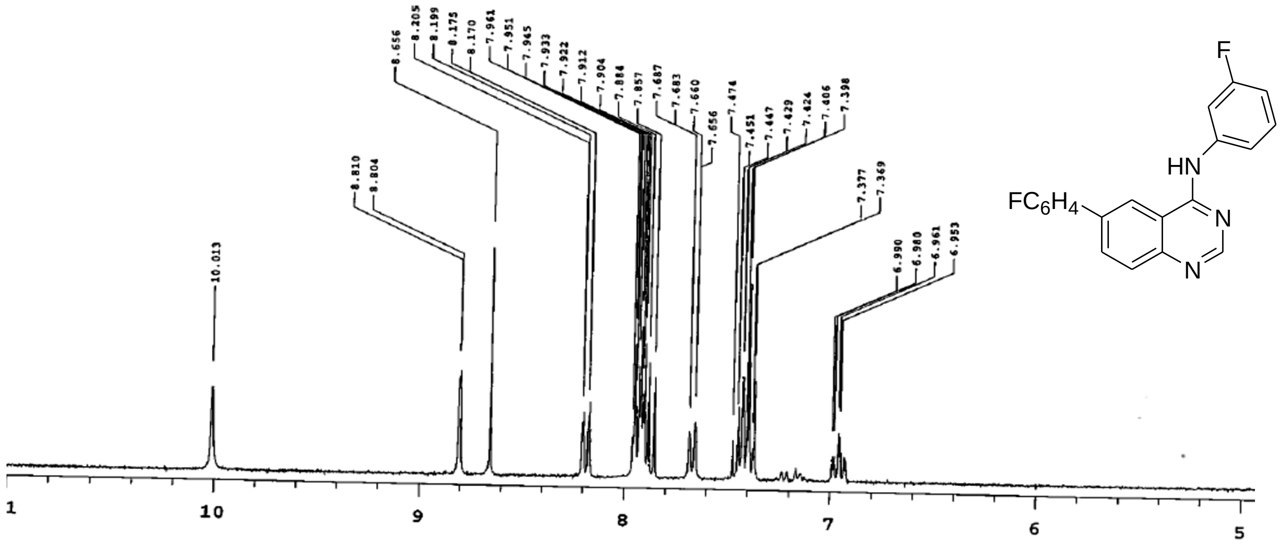

Figure 27. ^{1}H NMR spectrum of 4b in DMSO-d_6 at 500 MHz.

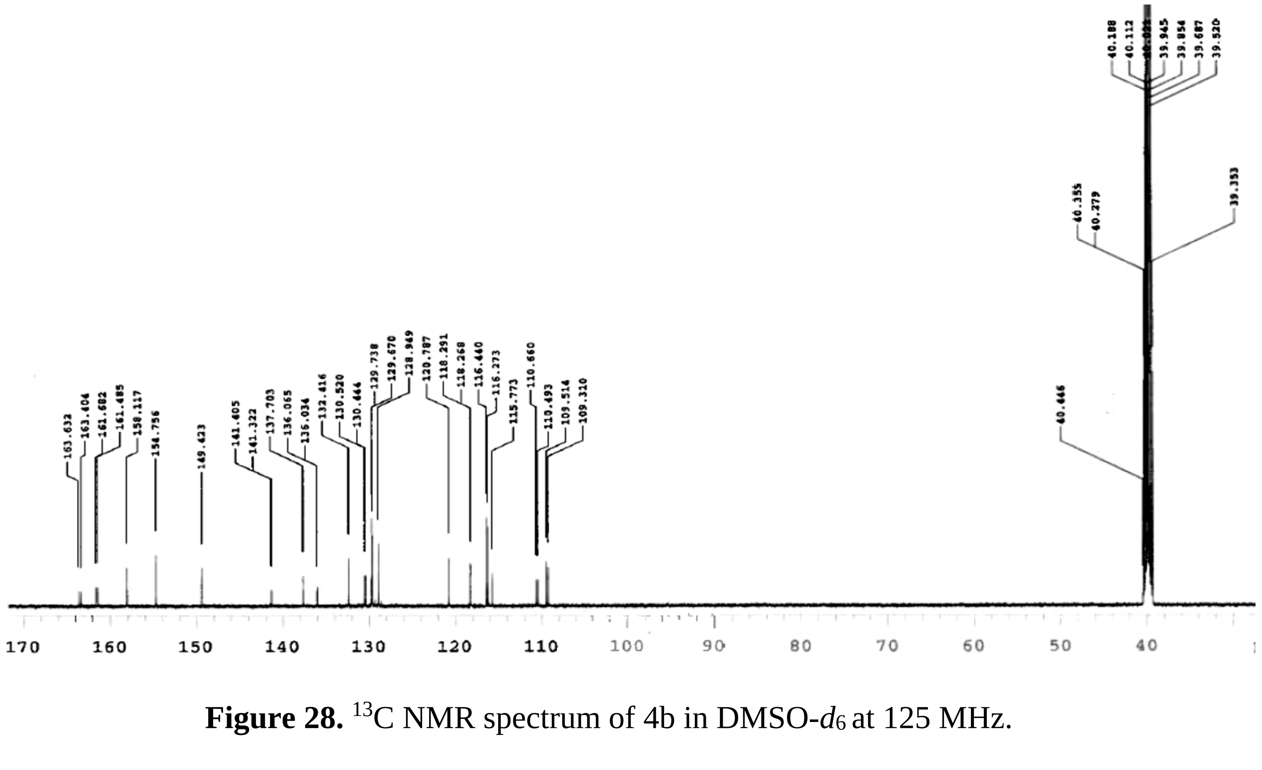

Figure 28. ^{13}C NMR spectrum of 4b in DMSO-d_6 at 125 MHz.

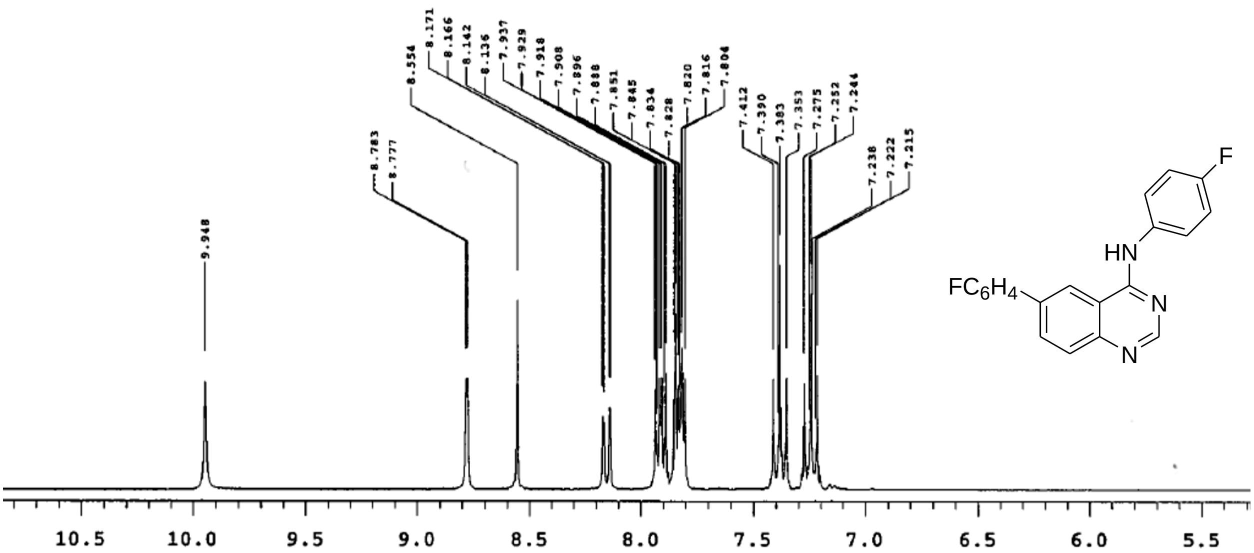

Figure 29. ^{1}H NMR spectrum of 4c in DMSO-d_6 at 500 MHz.

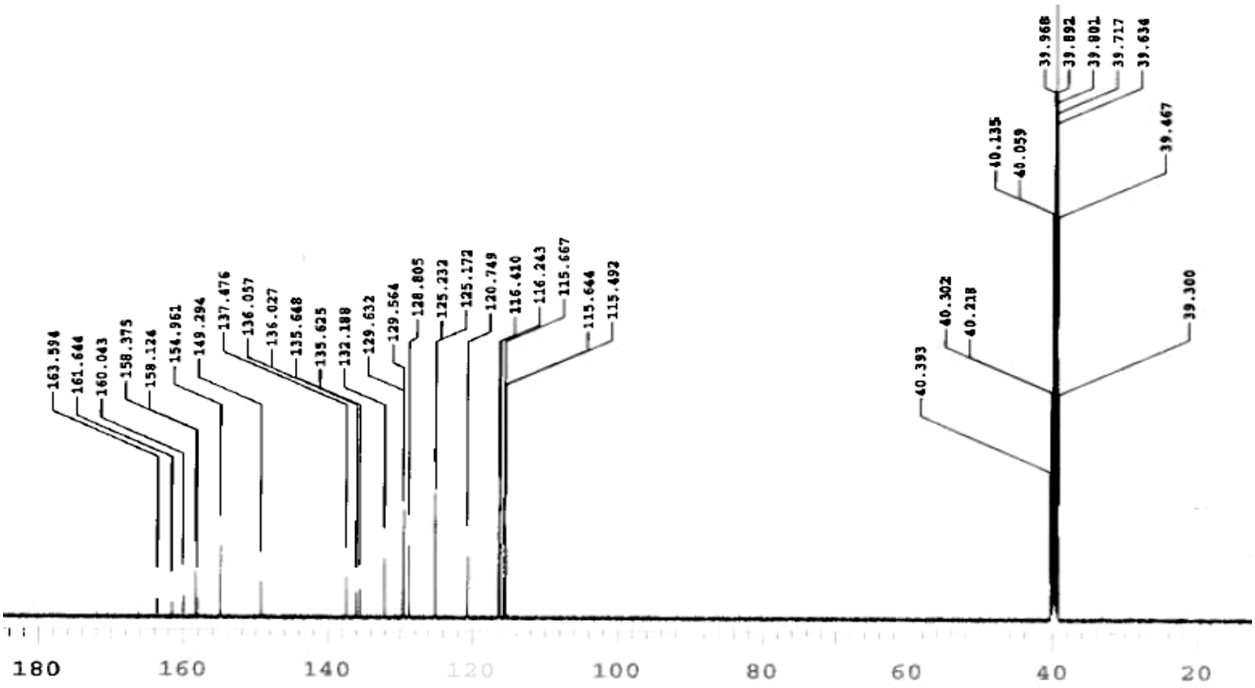

Figure 30. ^{13}C NMR spectrum of 4c in DMSO-d_6 at 125 MHz.

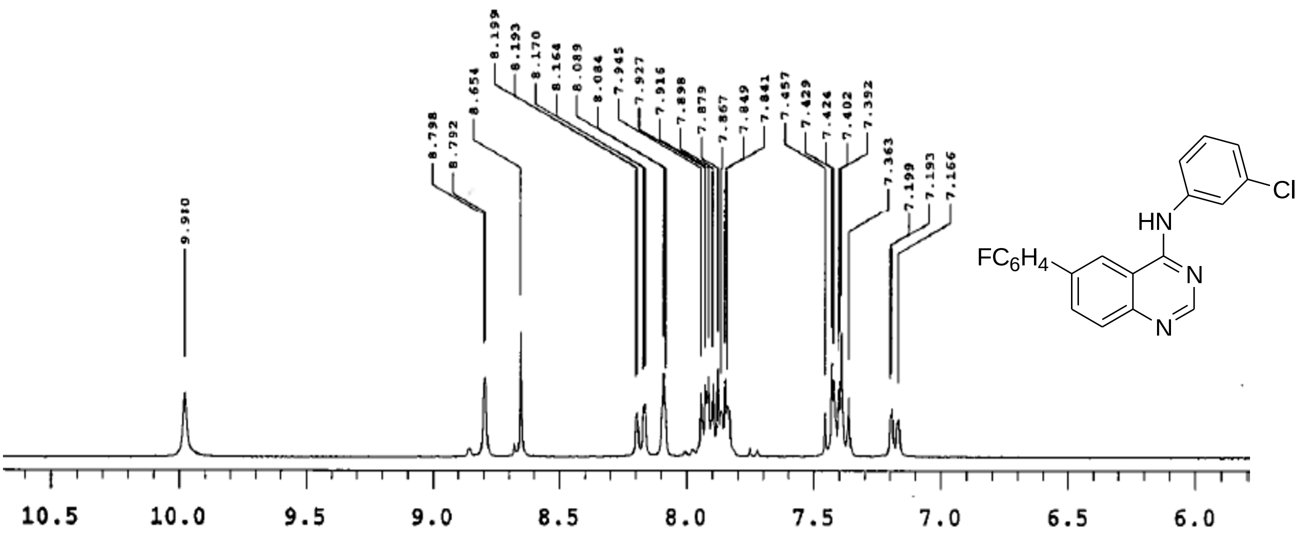

Figure 31. ^{1}H NMR spectrum of 4d in DMSO-d_6 at 500 MHz.

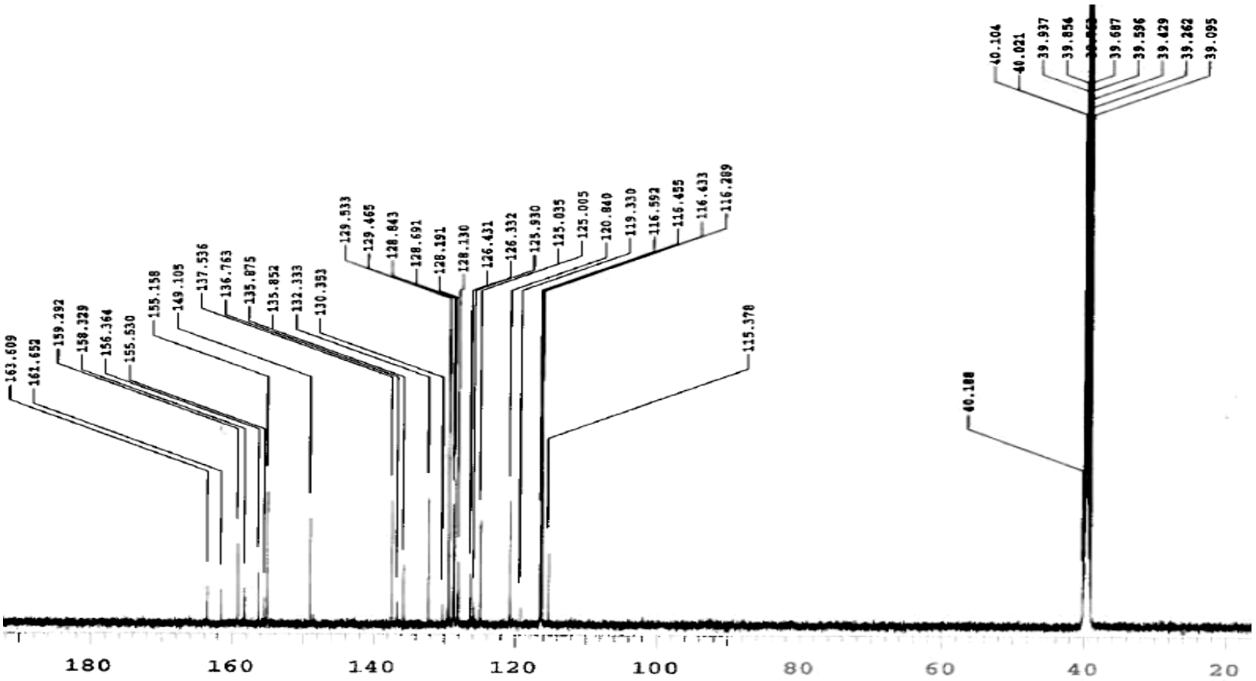

Figure 32. ^{13}C NMR spectrum of 4d in DMSO-d_6 at 125 MHz.

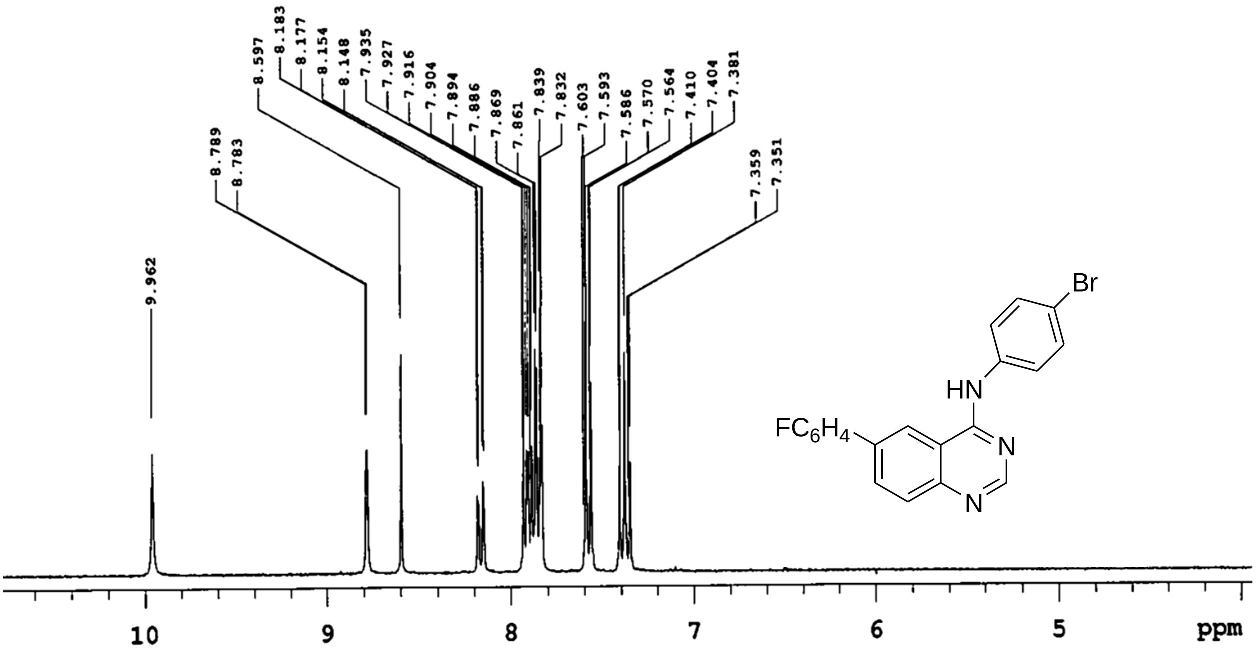

Figure 33. ^{1}H NMR spectrum of 4e in DMSO-d_6 at 500 MHz.

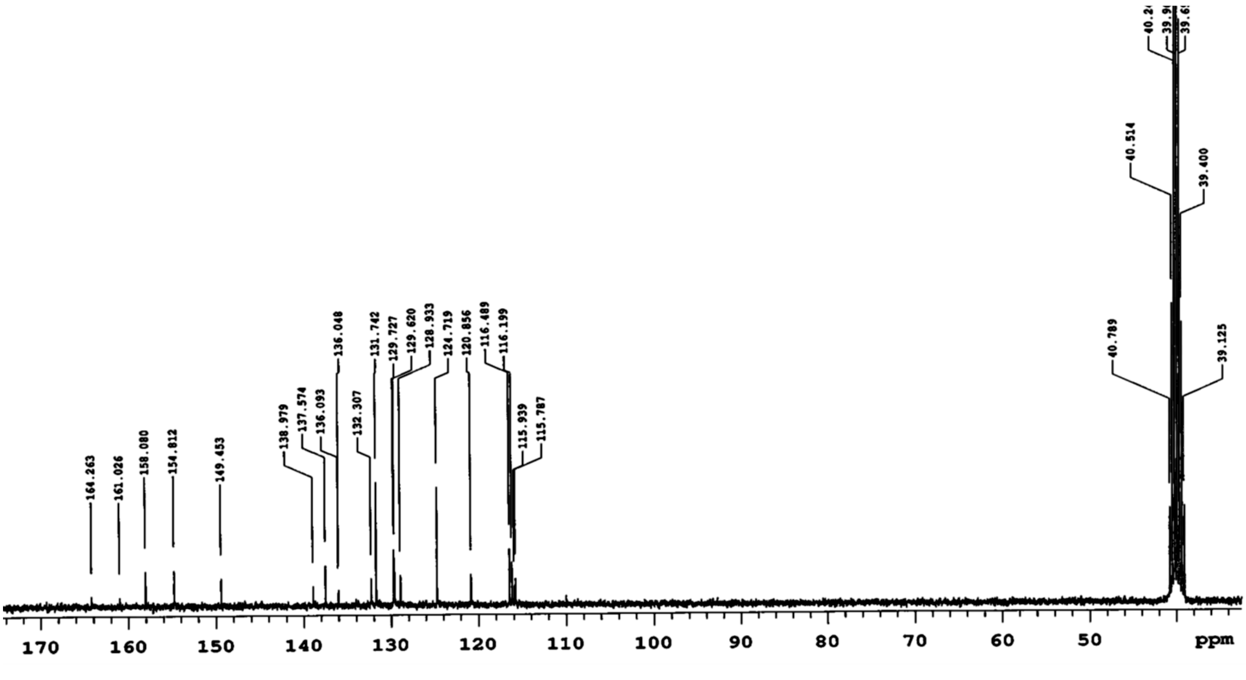

Figure 34. ^{13}C NMR spectrum of 4e in DMSO-d_6 at 125 MHz.

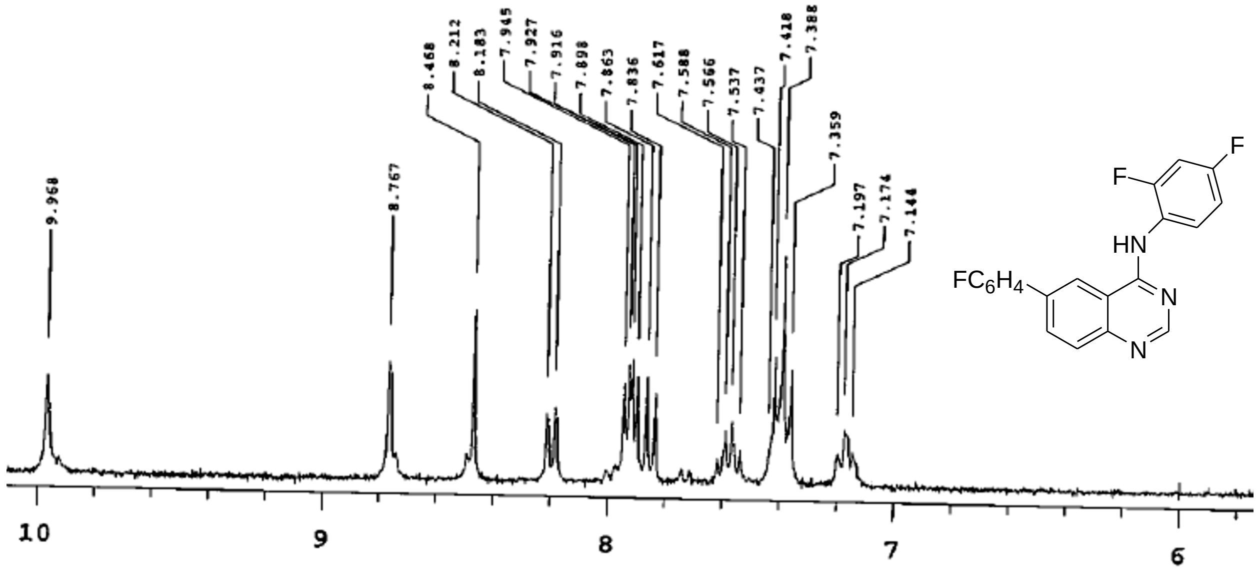

Figure 35. ^{1}H NMR spectrum of 4f in DMSO-d_6 at 500 MHz.

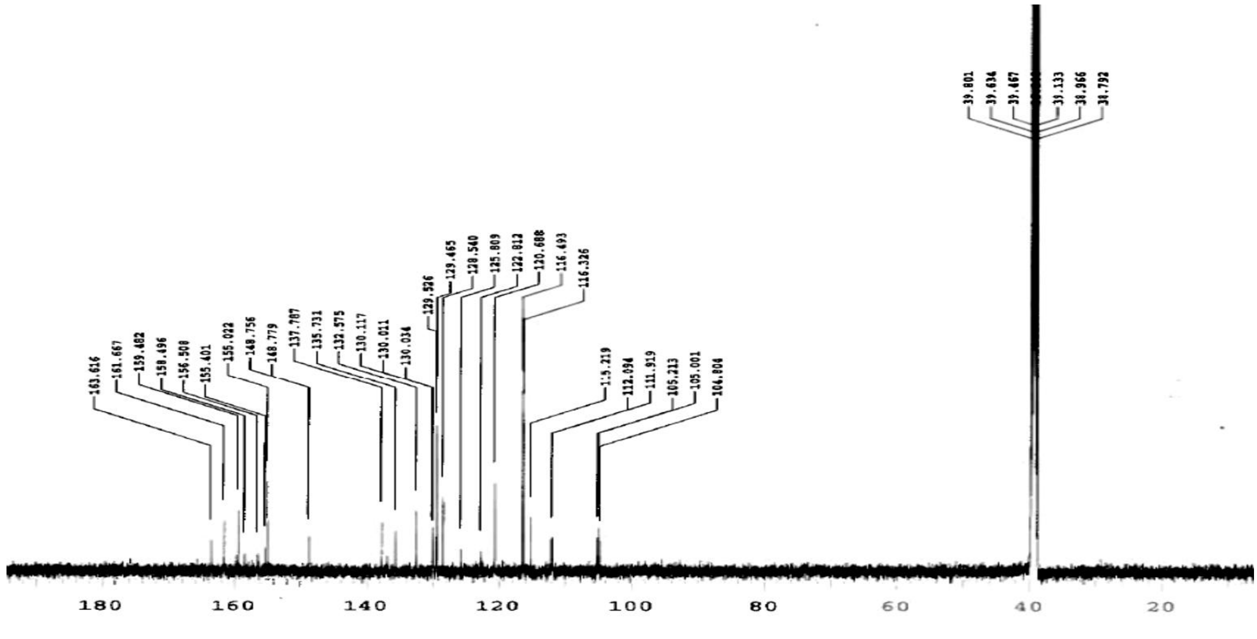

Figure 36. ^{13}C NMR spectrum of 4f in DMSO-d_6 at 125 MHz.

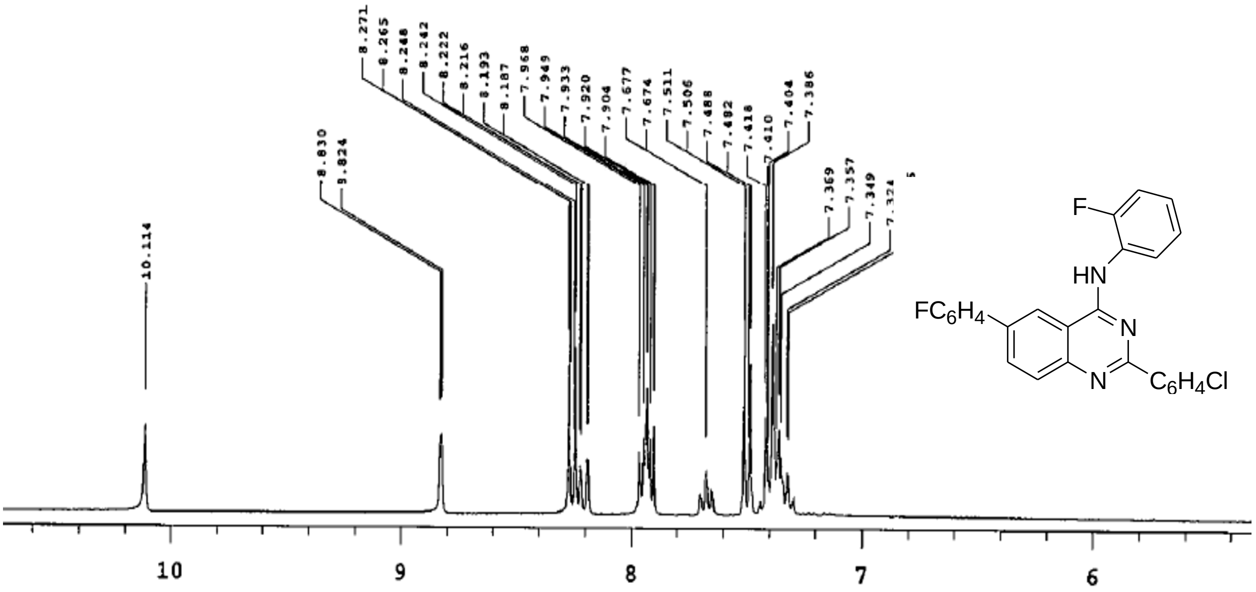

Figure 37. ^{1}H NMR spectrum of 4g in DMSO-d_6 at 500 MHz.

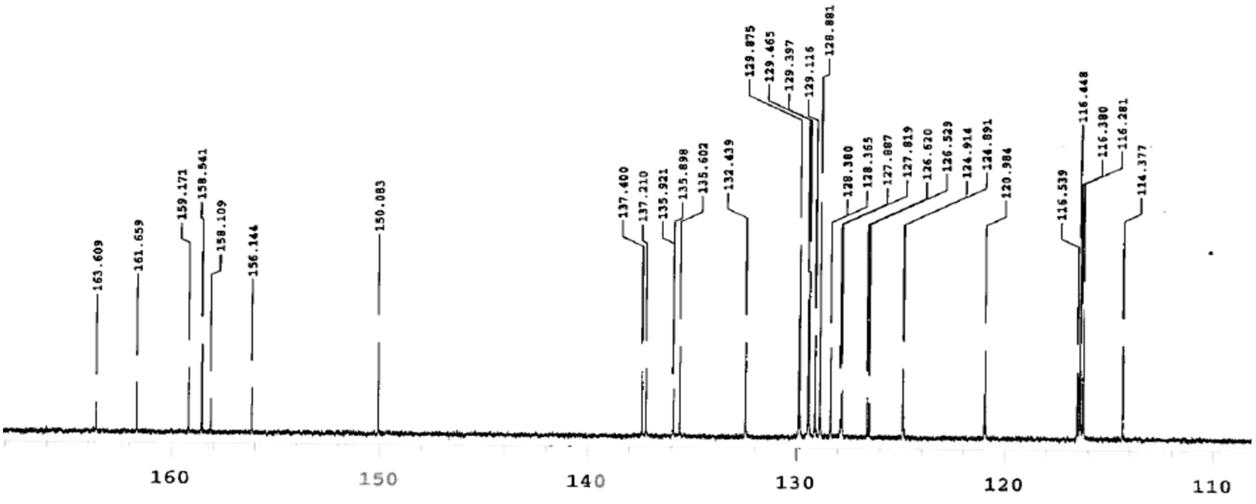

Figure 38. ^{13}C NMR spectrum of 4g in DMSO-d_6 at 125 MHz.

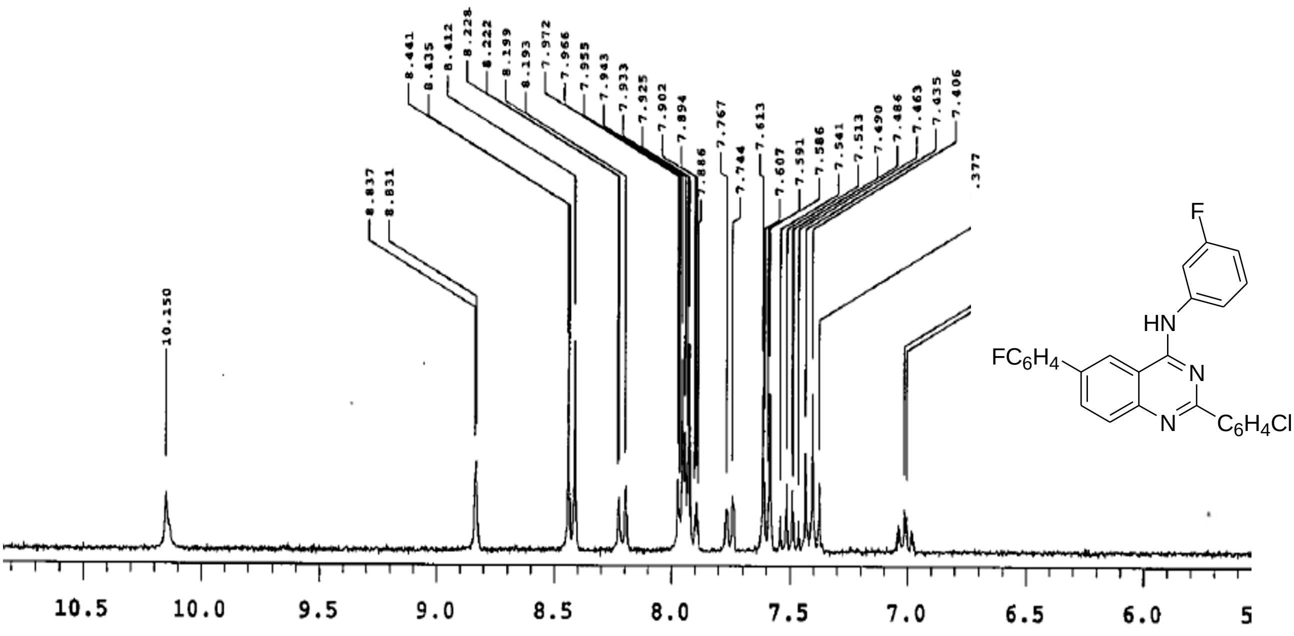

Figure 39. ^{1}H NMR spectrum of 4h in DMSO-d_6 at 500 MHz.

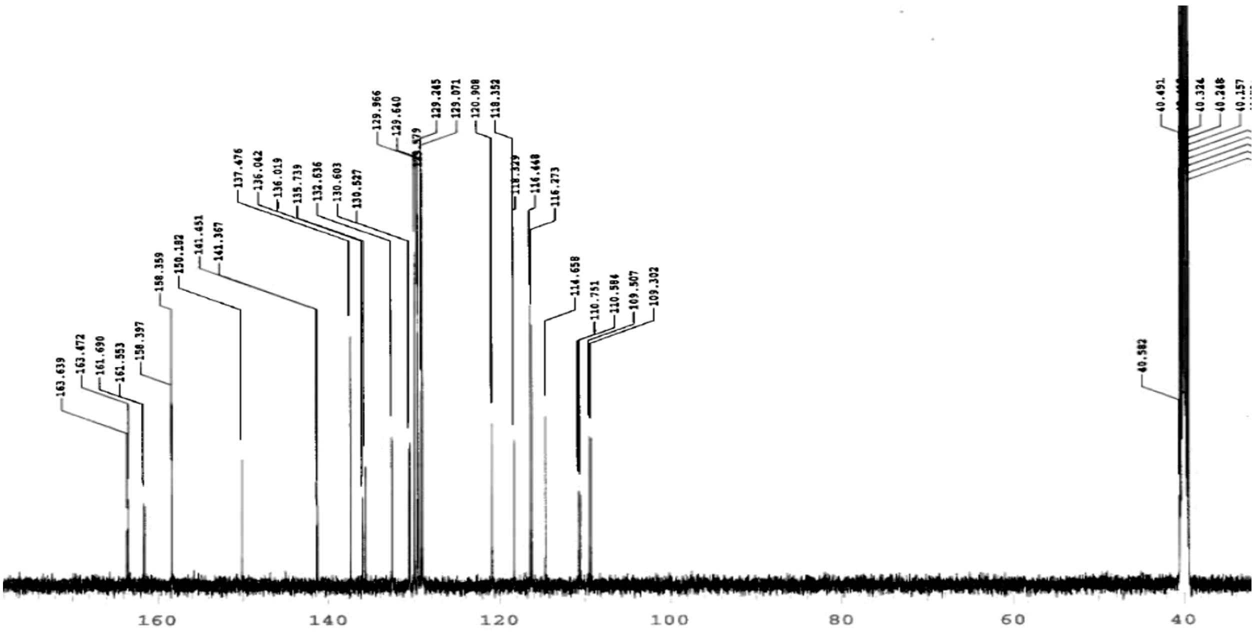

Figure 40. ^{13}C NMR spectrum of 4h in DMSO-d_6 at 125 MHz.

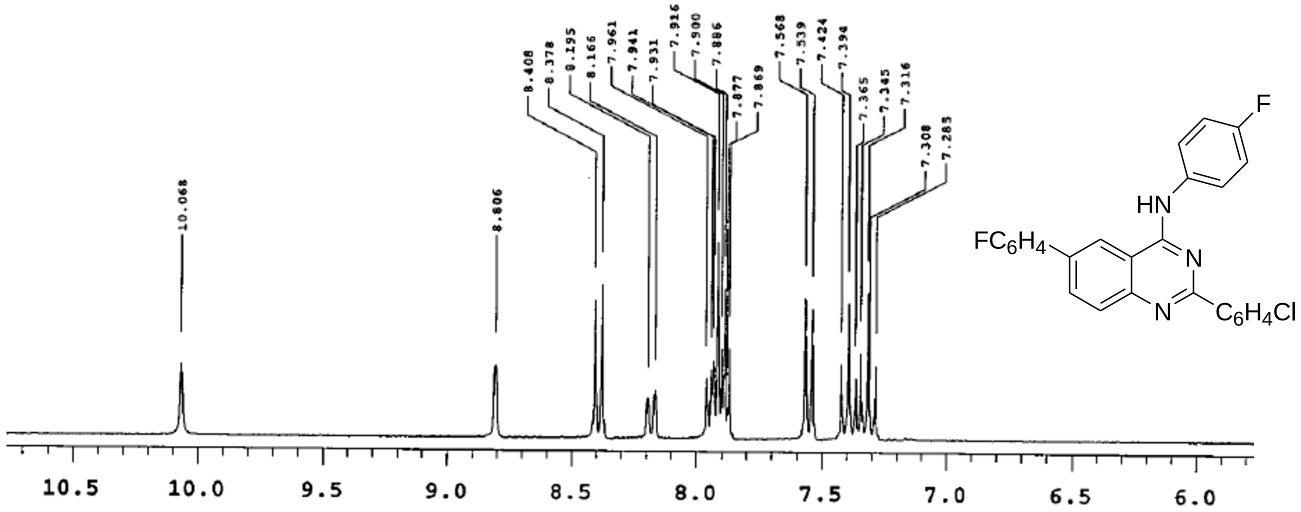

Figure 41. ^{1}H NMR spectrum of 4i in DMSO-d_6 at 500 MHz.

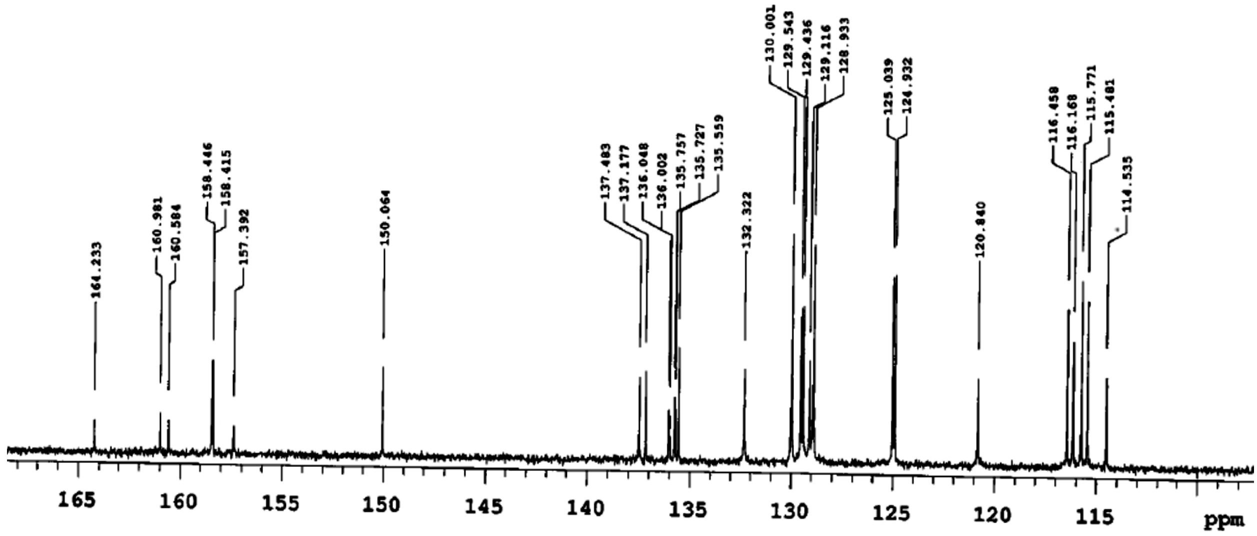

Figure 42. ^{13}C NMR spectrum of 4i in DMSO-d_6 at 125 MHz.

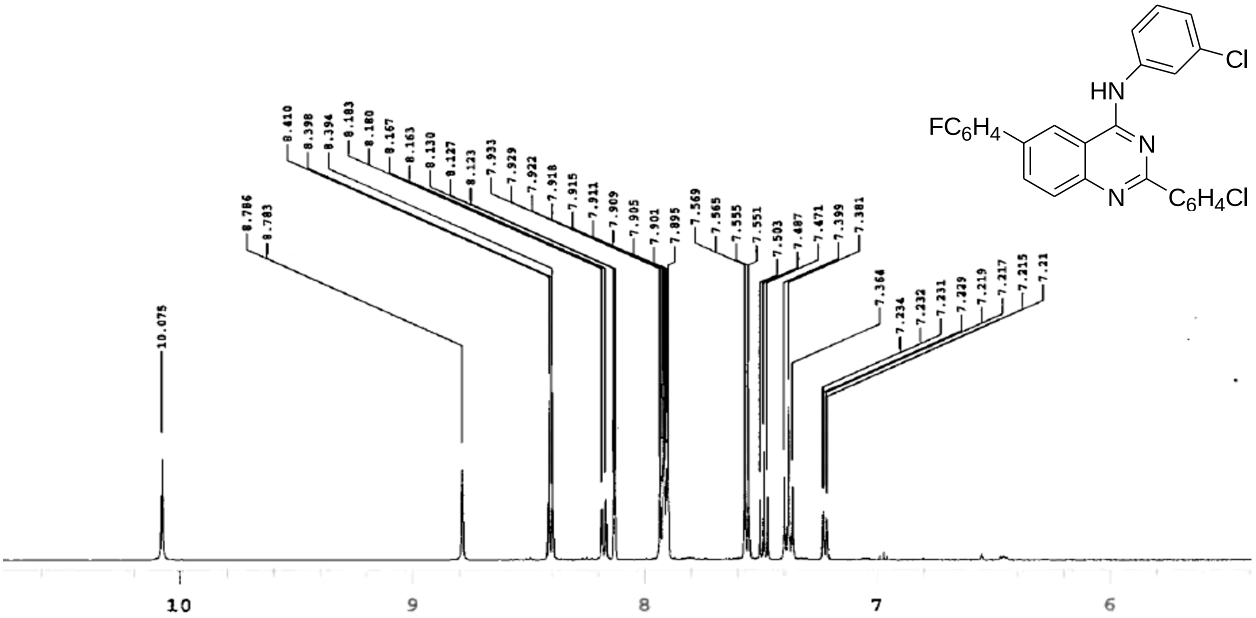

Figure 43. ^{1}H NMR spectrum of 4j in DMSO-d_6 at 500 MHz.

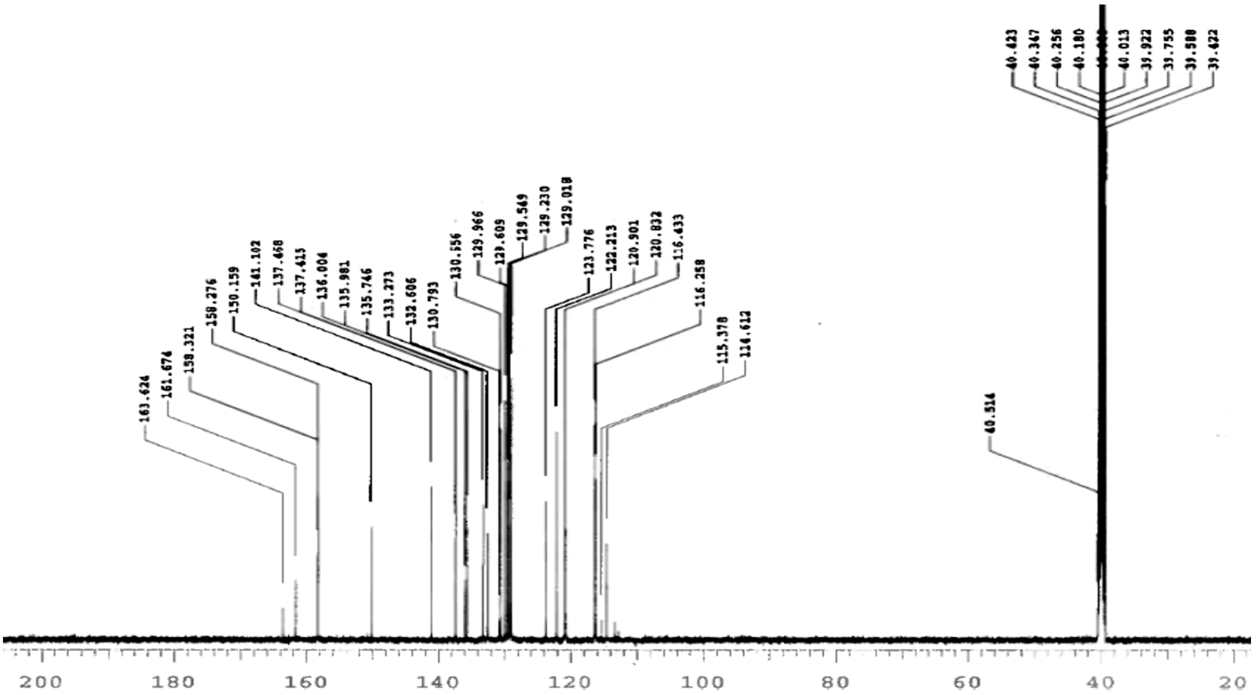

Figure 44. ^{13}C NMR spectrum of 4j in DMSO-d_6 at 125 MHz.

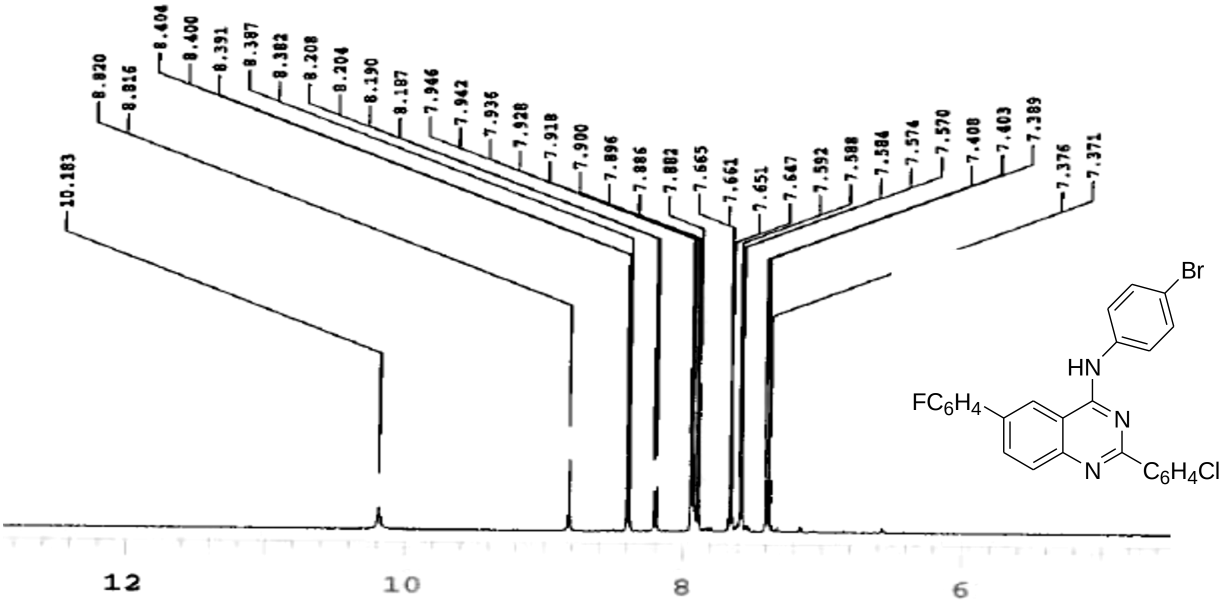

Figure 45. ^{1}H NMR spectrum of 4k in DMSO-d_6 at 500 MHz.

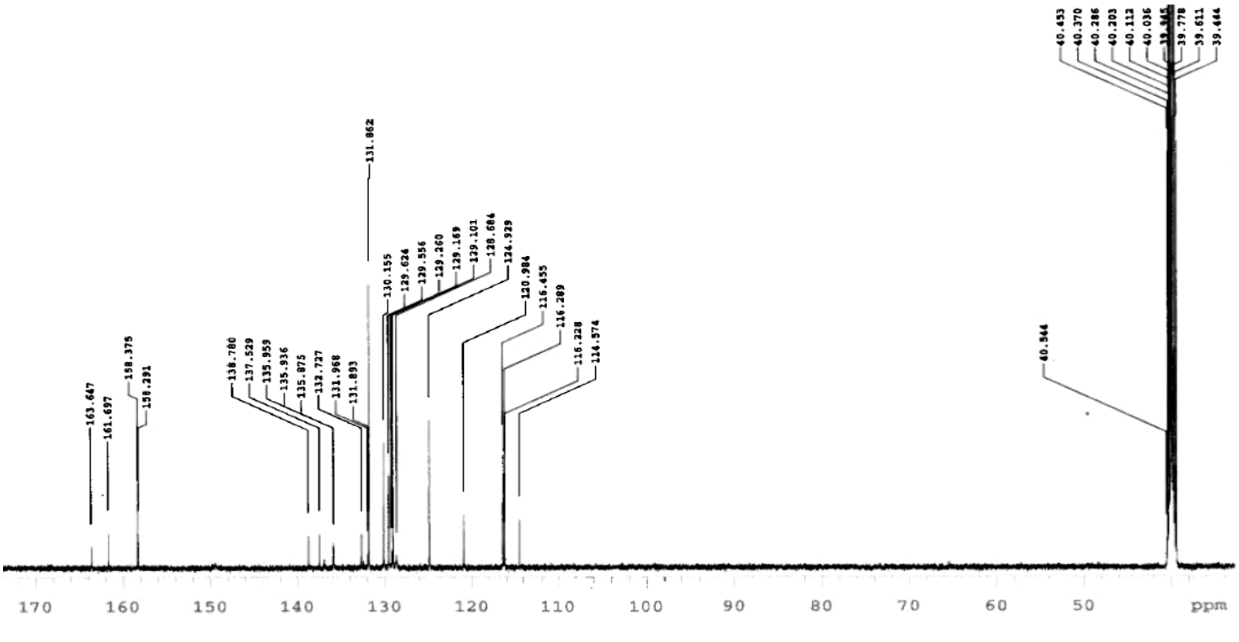

Figure 46. ^{13}C NMR spectrum of 4k in DMSO-d_6 at 125 MHz.

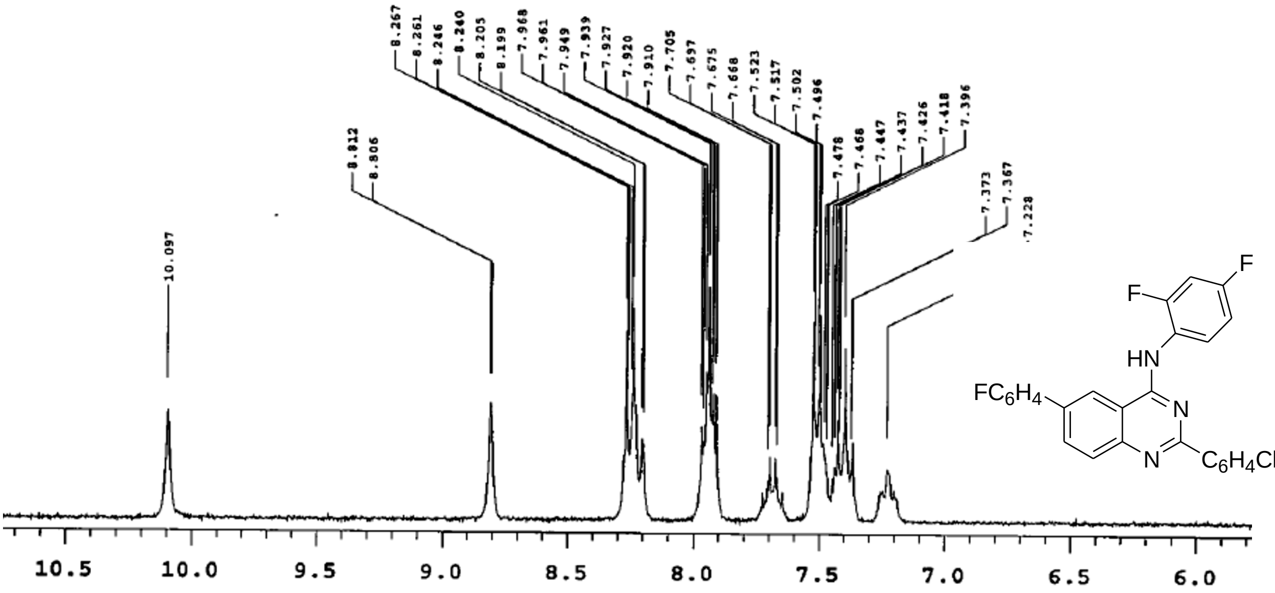

Figure 47. ^{1}H NMR spectrum of 4l in DMSO-d_6 at 500 MHz.

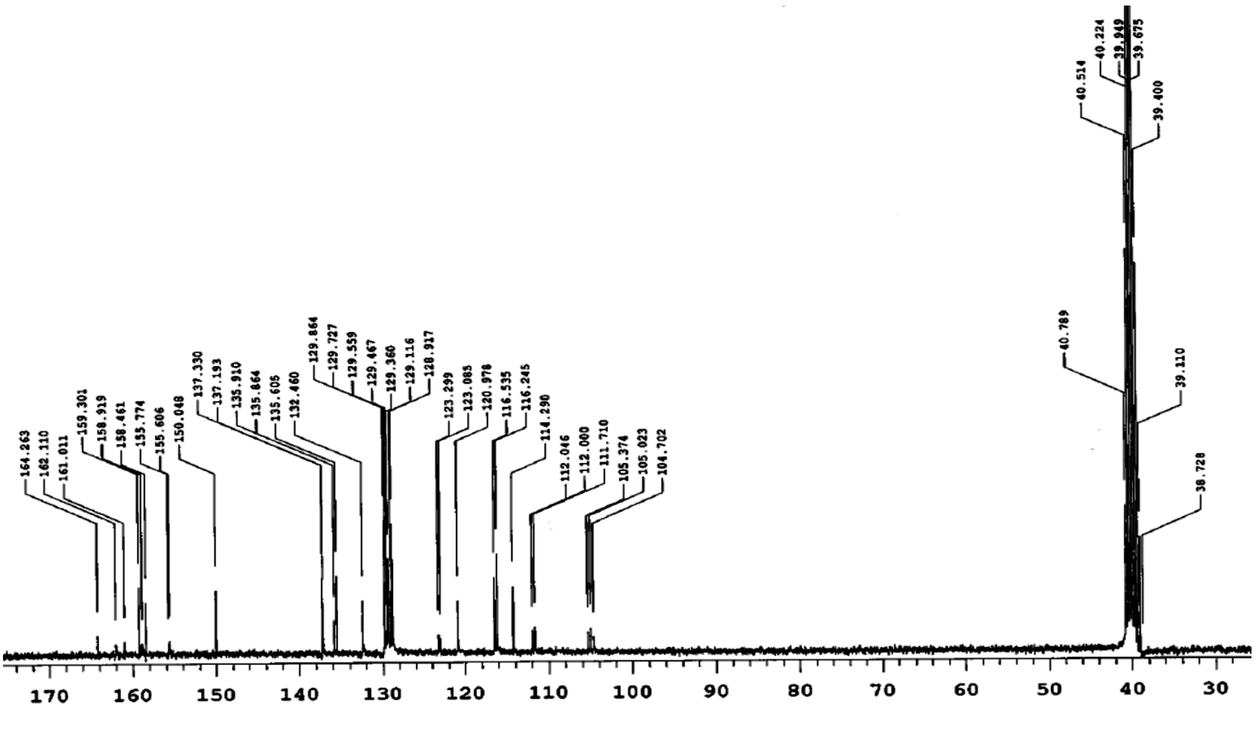

Figure 48. ^{13}C NMR spectrum of 4l in DMSO-d_6 at 125 MHz.

S2. % Cell Viability of HeLa and MCF-7 Cells Exposed to Gefitinib and Compounds 3a-l and 4a-l

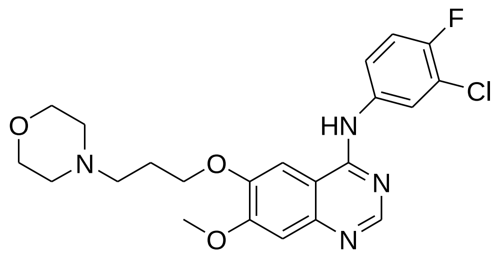

Gefitinib

Table 1. Percentage cell viability of HeLa and MCF-7 cells exposed to different concentrations of Gefitinib.

Conc. (μM)	%Viability HeLa	SD	%Viability MCF-7	SD
100	7.24	0.5	9.84	0.75
10	53.75	1.47	42.26	1.92
1	78.31	2.59	58.35	0.58
0.1	89.57	1.55	62.15	0.88
0.01	92.00	1.71	69.70	1.21
0.001	99.00	0.92	72.03	1.83

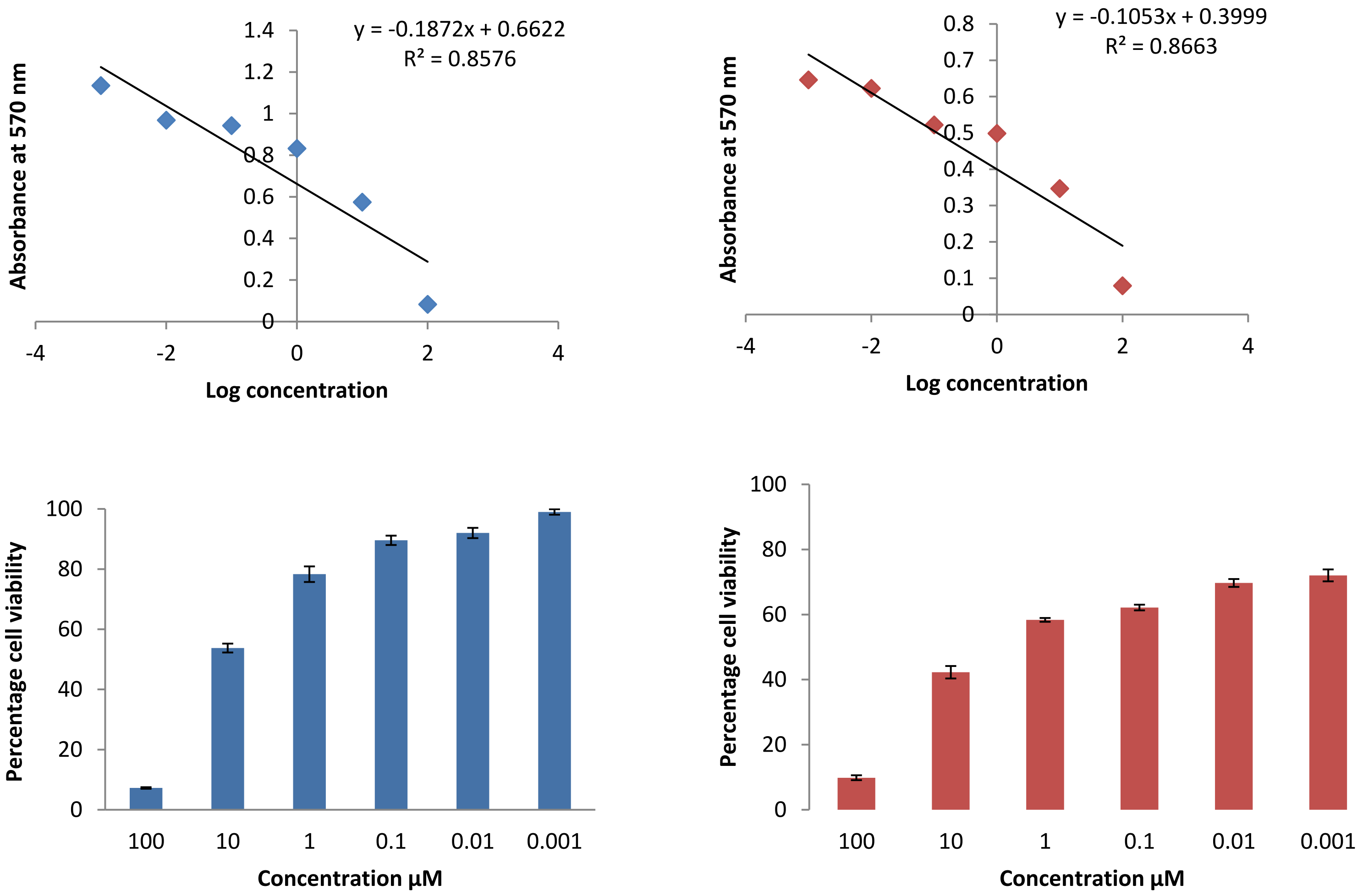

HeLa **MCF-7**

Figure 49. Linear regression plots and percentage cell viability graphs of HeLa and MCF-7 cells exposed to different concentrations of Getifinib.

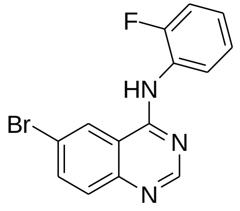

3a

Table 2. Percentage cell viability of HeLa and MCF-7 cells exposed to different concentrations of **3a**.

Conc. (µM)	%Viability HeLa	SD	%Viability MCF-7	SD
100	7.26	0.41	12.82	0.04
10	72.36	0.28	48.54	0.05
1	75.85	0.66	55.84	3.13
0.1	78.48	1.86	63.77	1.75

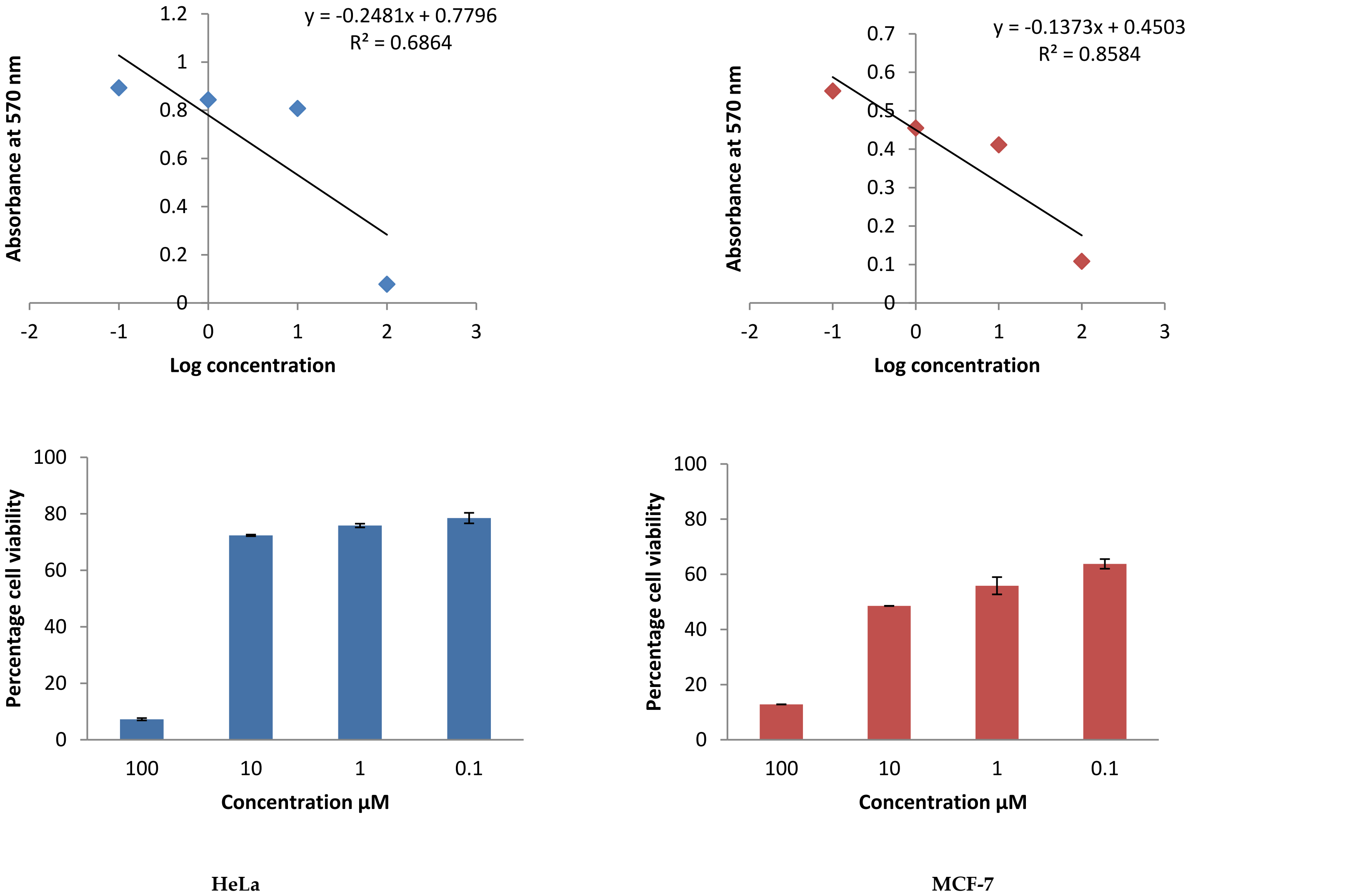

Figure 50. Linear regression plots and percentage cell viability graphs of HeLa and MCF-7 cells exposed to different concentrations of **3a**.

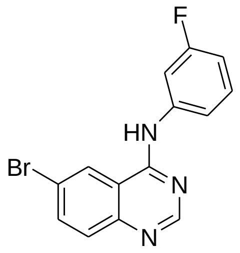

3b

Table 3. Percentage cell viability of HeLa and MCF-7 cells exposed to different concentrations of **3b**.

Conc. (μM)	%Viability HeLa	SD	%Viability MCF-7	SD
100	7.46	0.25	11.08	0.25
10	61.82	1.29	53.49	0.21
1	66.08	0.82	62.36	1.99
0.1	72.16	1.04	67.63	2.54

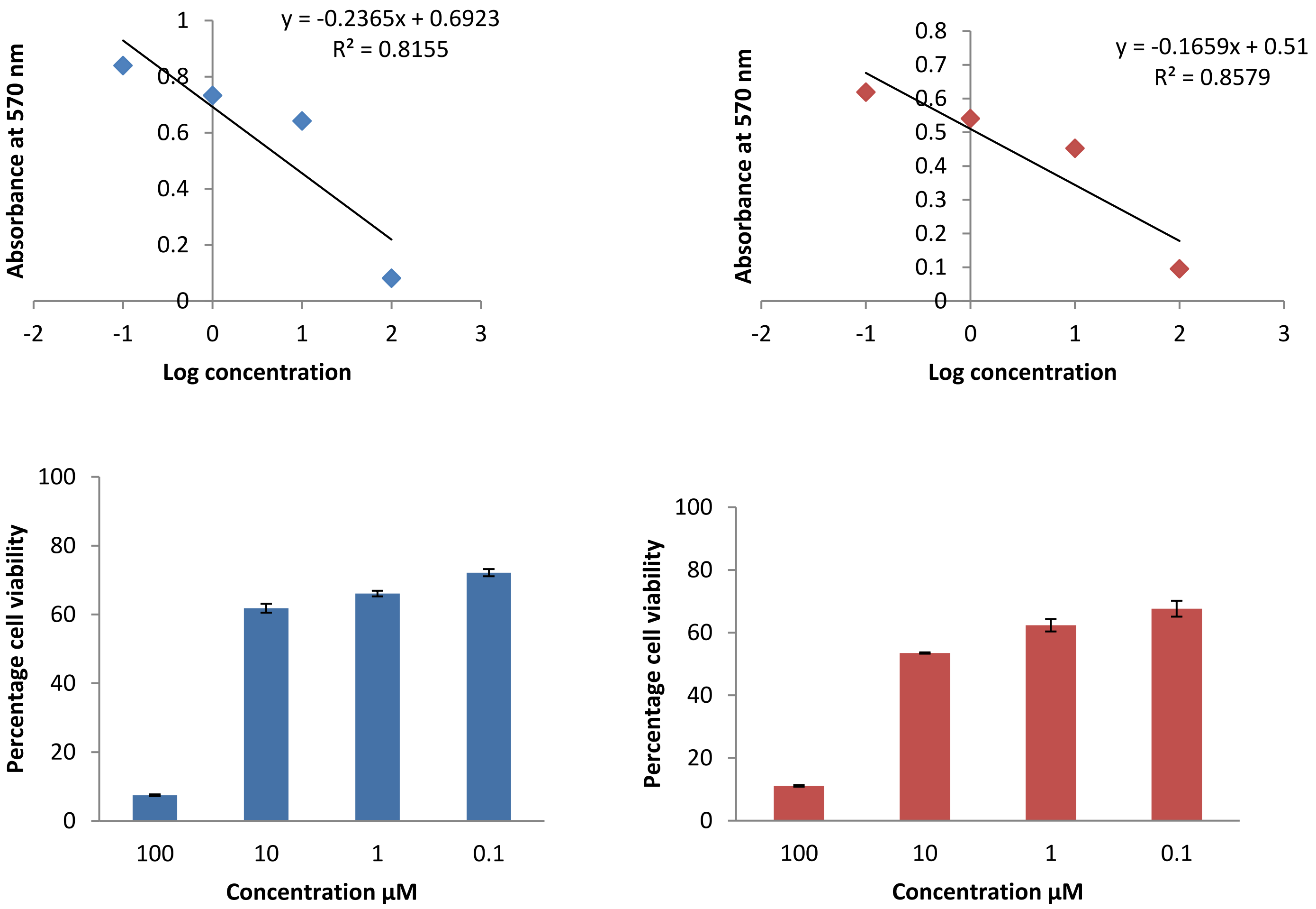

HeLa **MCF-7**

Figure 51. Linear regression plots and percentage cell viability graphs of HeLa and MCF-7 cells exposed to different concentrations of **3b**.

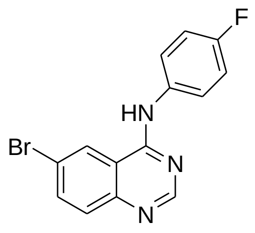

3c

Table 4. Percentage cell viability of HeLa and MCF-7 cells exposed to different concentrations of **3c**.

Conc. (μM)	%Viability HeLa	SD	%Viability MCF-7	SD
100	8.60	0.47	15.82	1.04
10	48.10	2.37	41.23	0.80
1	67.81	0.32	66.57	1.12
0.1	76.50	2.18	80.27	2.74

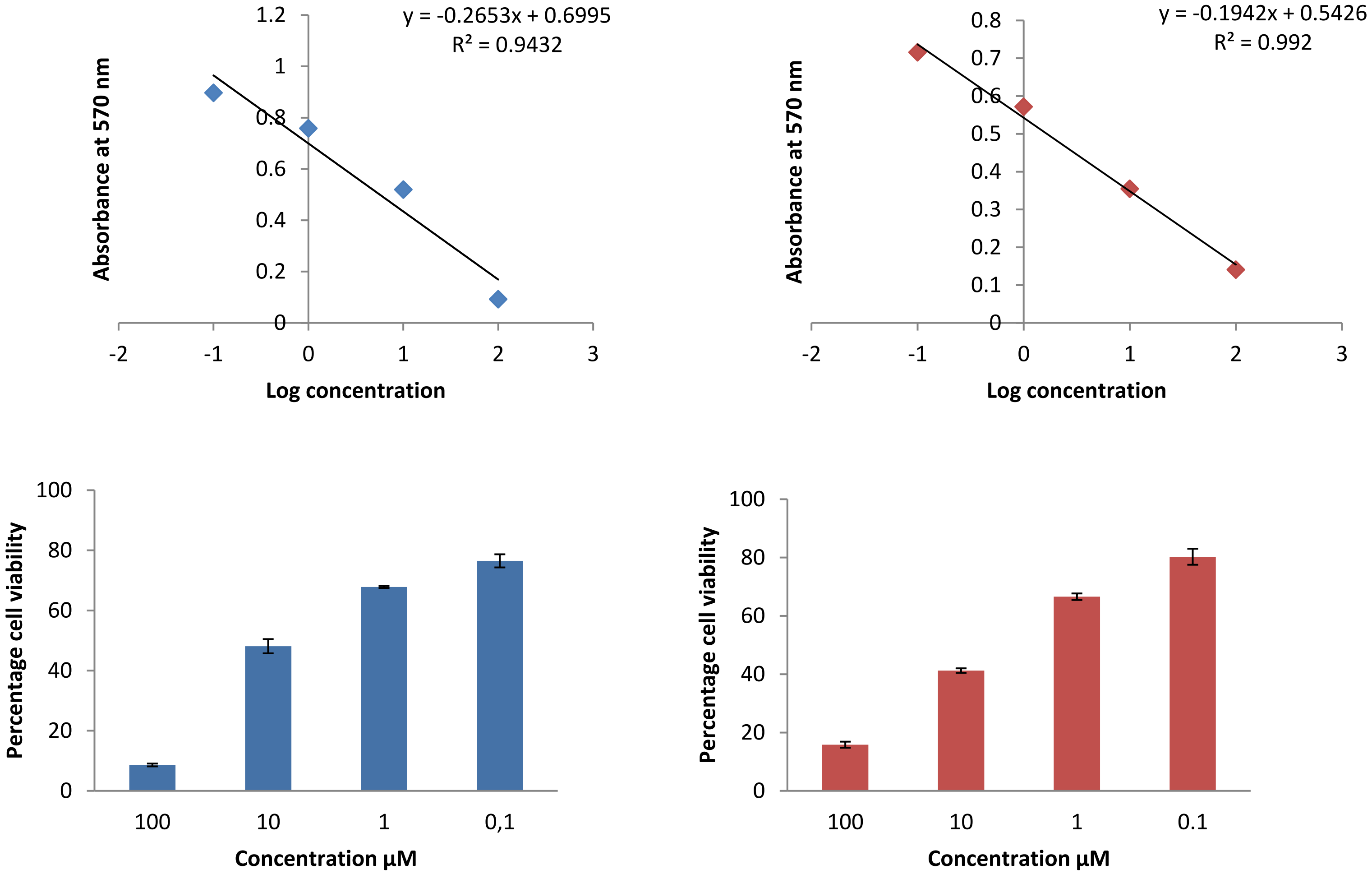

HeLa **MCF-7**

Figure 52. Linear regression plots and percentage cell viability graphs of HeLa and MCF-7 cells exposed to different concentrations of **3c**.

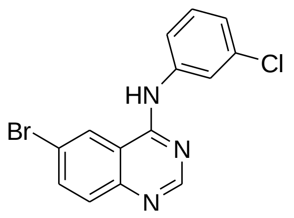

3d

Table 5. Percentage cell viability of HeLa and MCF-7 cells exposed to different concentrations of **3d**.

Conc. (μM)	%Viability HeLa	SD	%Viability MCF-7	SD
100	7.60	0.63	14.03	0.50
10	56.88	1.58	58.26	2.12
1	81.79	1.76	64.74	2.90
0.1	92.05	0.69	72.32	2.91

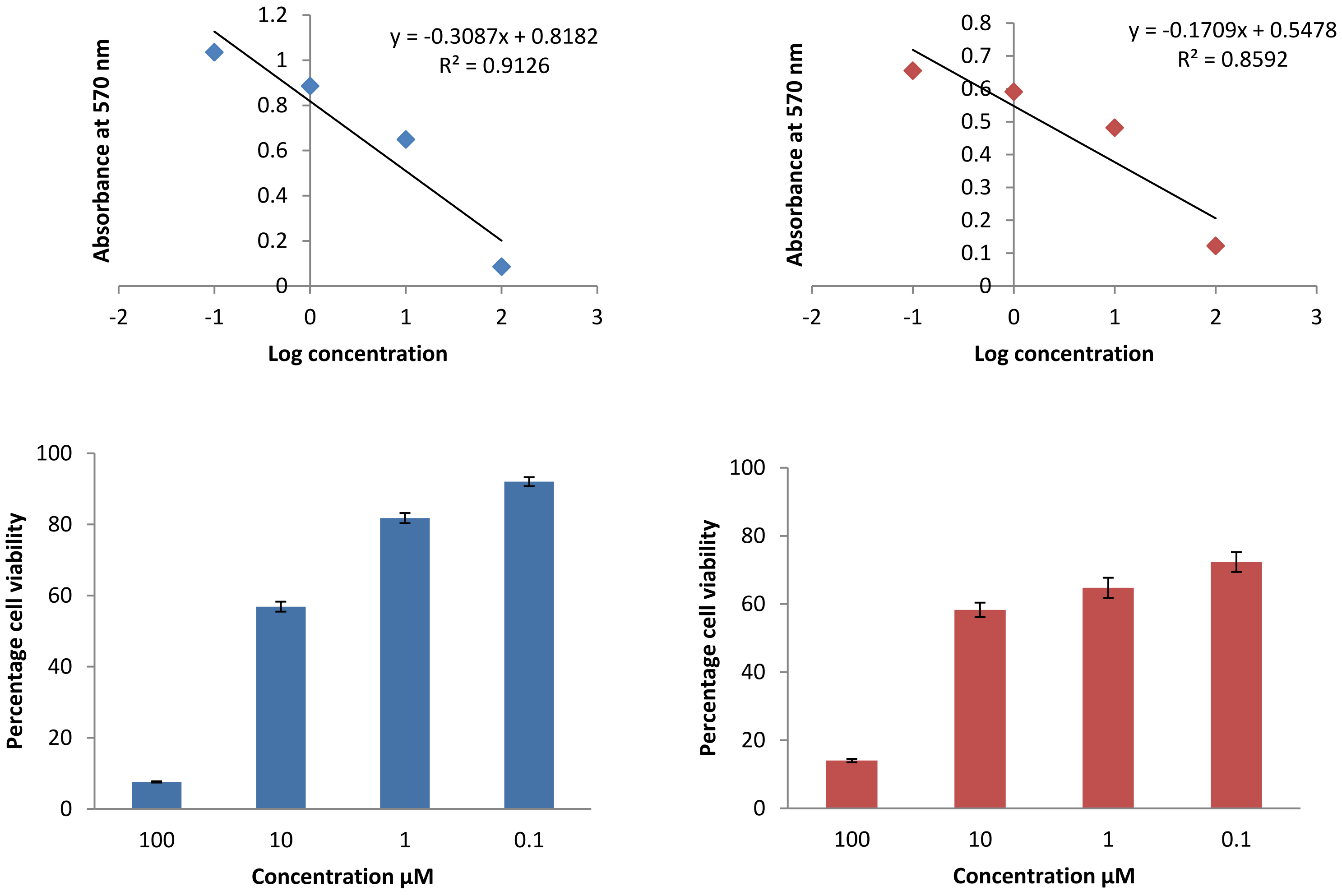

HeLa **MCF-7**

Figure 53. Linear regression plots and percentage cell viability graphs of HeLa and MCF-7 cells exposed to different concentrations of **3d**.

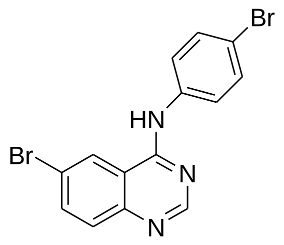

3e

Table 6. Percentage cell viability of HeLa and MCF-7 cells exposed to different concentrations of **3e**.

Conc. (µg/ml)	%Viability HeLa	SD	%Viability MCF-7	SD
100	7.06	0.20	11.20	0.33
10	67.54	1.40	55.17	2.17
1	94.28	1.42	84.78	1.70
0.1	97.41	1.26	99.22	1.11

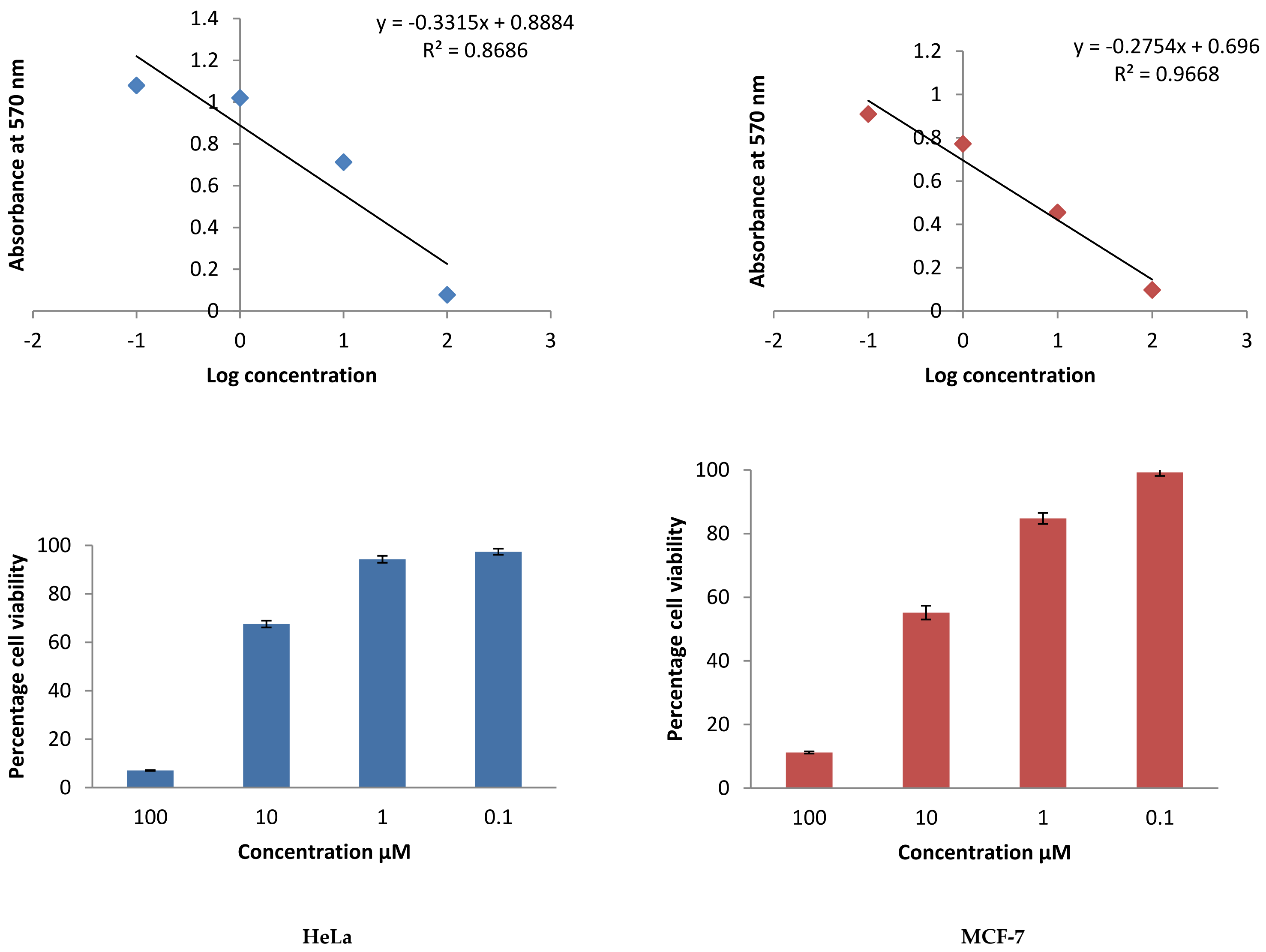

Figure 54. Linear regression plots and percentage cell viability graphs of HeLa and MCF-7 cells exposed to different concentrations of **3e**.

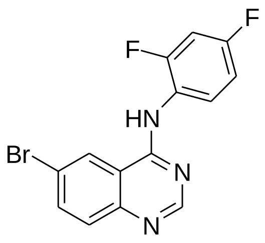

3f

Table 7. Percentage cell viability of HeLa and MCF-7 cells exposed to different concentrations of **3f**.

Conc. (μM)	%Viability HeLa	SD	%Viability MCF-7	SD
100	7.91	0.60	11.35	0.04
10	65.86	0.38	43.62	0.02
1	68.61	1.24	55.96	1.63
0.1	79.91	2.35	66.57	0.21

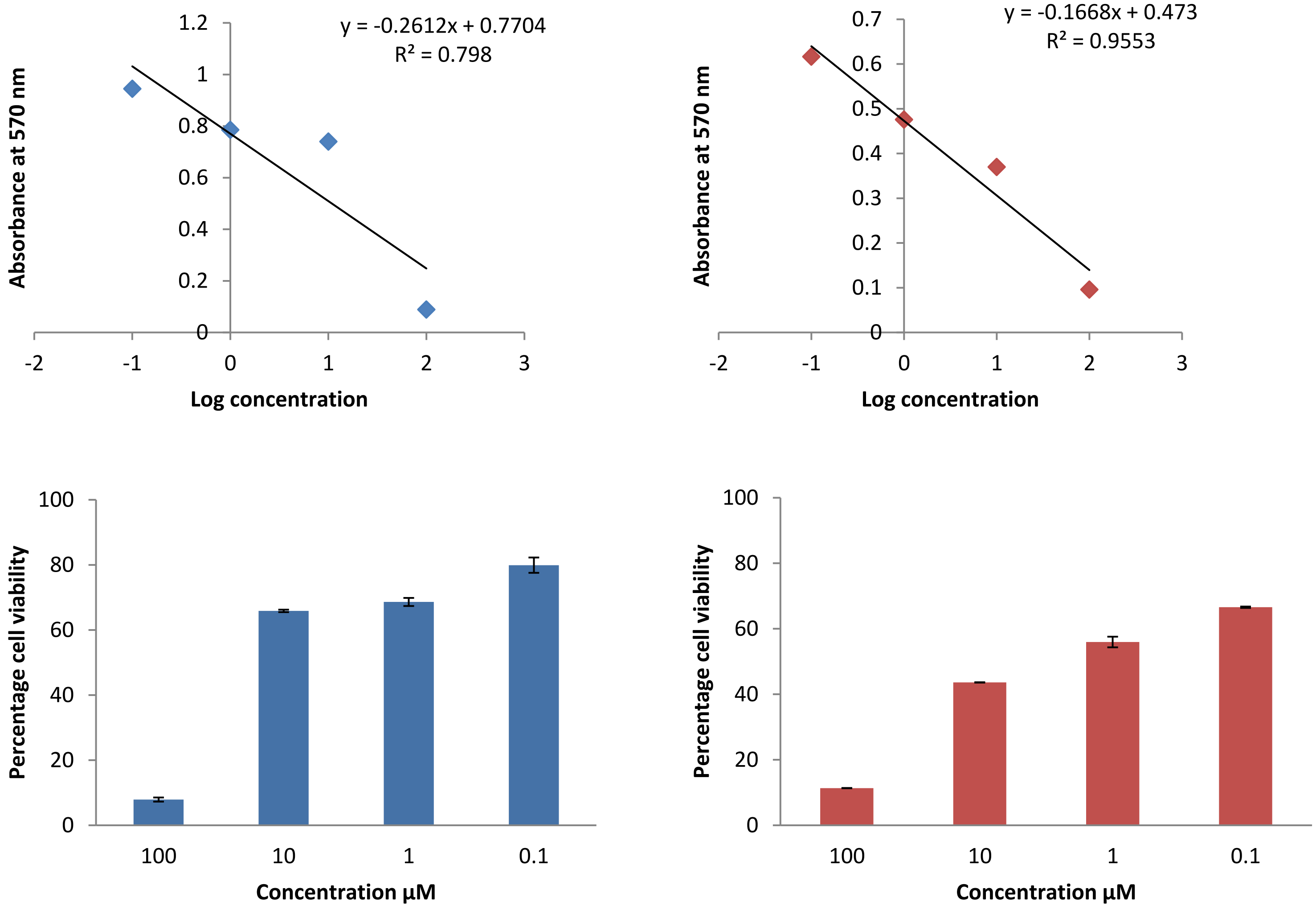

HeLa **MCF-7**

Figure 55. Linear regression plots and percentage cell viability graphs of HeLa and MCF-7 cells exposed to different concentrations of **3f**.

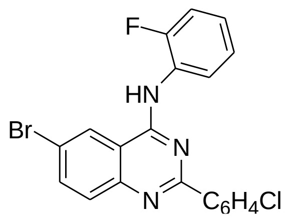

3g

Table 8. Percentage cell viability of HeLa and MCF-7 cells exposed to different concentrations of **3g**.

Conc. (µM)	%Viability HeLa	SD	%Viability MCF-7	SD
100	10.84	1.49	13.57	0.11
10	33.76	1.94	35.28	0.71
1	49.46	1.58	38.66	3.67
0.1	58.50	1.45	50.57	1.08

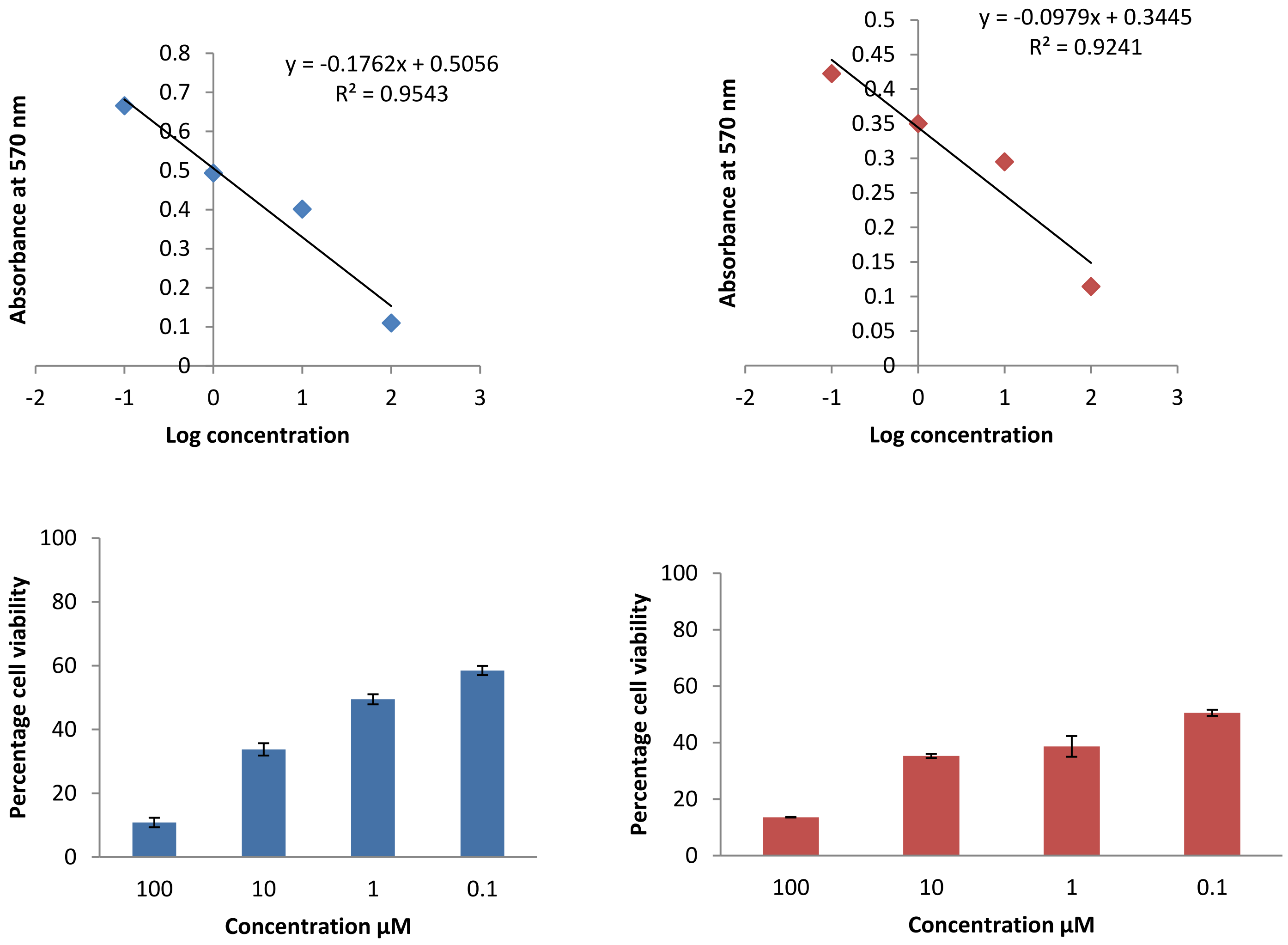

HeLa **MCF-7**

Figure 56. Linear regression plots and percentage cell viability graphs of HeLa and MCF-7 cells exposed to different concentrations of **3g**.

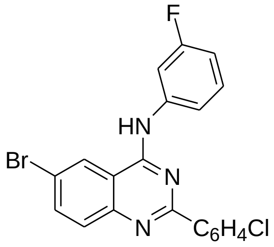

3h

Table 9. Percentage cell viability of HeLa and MCF-7 cells exposed to different concentrations of **3h**.

Conc. (µM)	%Viability HeLa	SD	%Viability MCF-7	SD
100	11.33	0.16	17.74	0.08
10	31.05	1.55	31.92	1.62
1	55.12	1.84	46.24	2.63
0.1	73.86	1.18	63.10	0.63

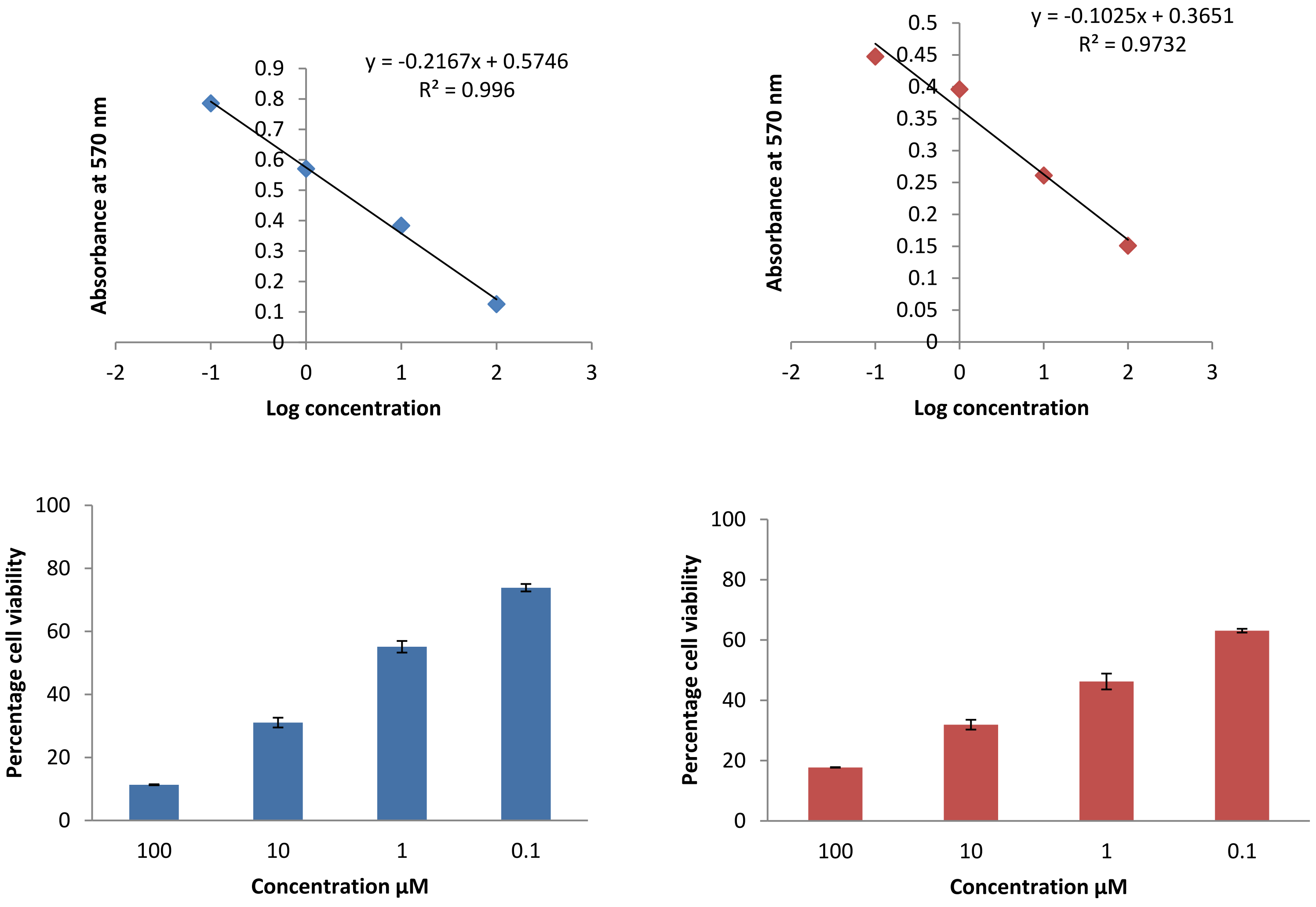

HeLa MCF-7

Figure 57. Linear regression plots and percentage cell viability graphs of HeLa and MCF-7 cells exposed to different concentrations of **3h**.

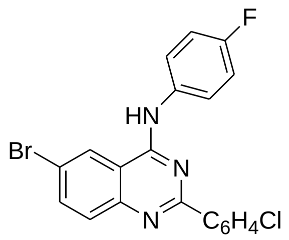

3i

Table 10. Percentage cell viability of HeLa and MCF-7 cells exposed to different concentrations of **3i**.

Conc. (μM)	%Viability HeLa	SD	%Viability MCF-7	SD
100	11.51	0.95	12.55	3.00
10	39.36	1.71	33.63	3.96
1	81.30	1.56	50.31	3.30
0.1	89.61	1.03	68.52	1.63

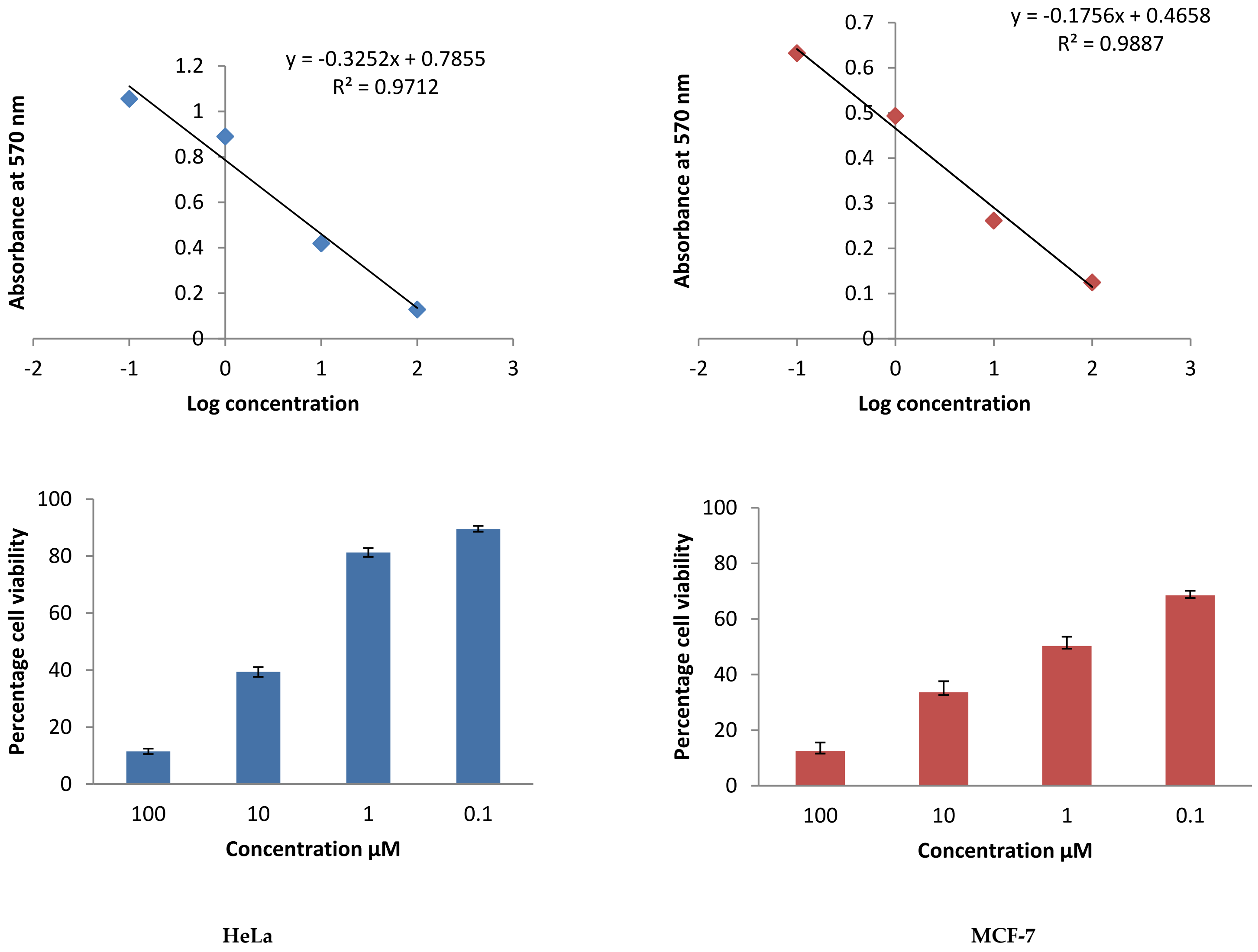

Figure 58. Linear regression plots and percentage cell viability graphs of HeLa and MCF-7 cells exposed to different concentrations of **3i**.

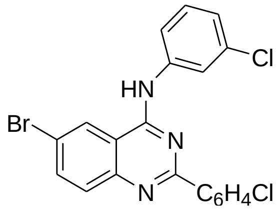

3j

Table 11. Percentage cell viability of HeLa and MCF-7 cells exposed to different concentrations of **3j**.

Conc. (µM)	%Viability HeLa	SD	%Viability MCF-7	SD
100	8.82	0.10	12.52	3.46
10	3302	0.50	33.69	3.13
1	59.67	194	50.34	2.09
0.1	92.18	2.86	69.79	2.25

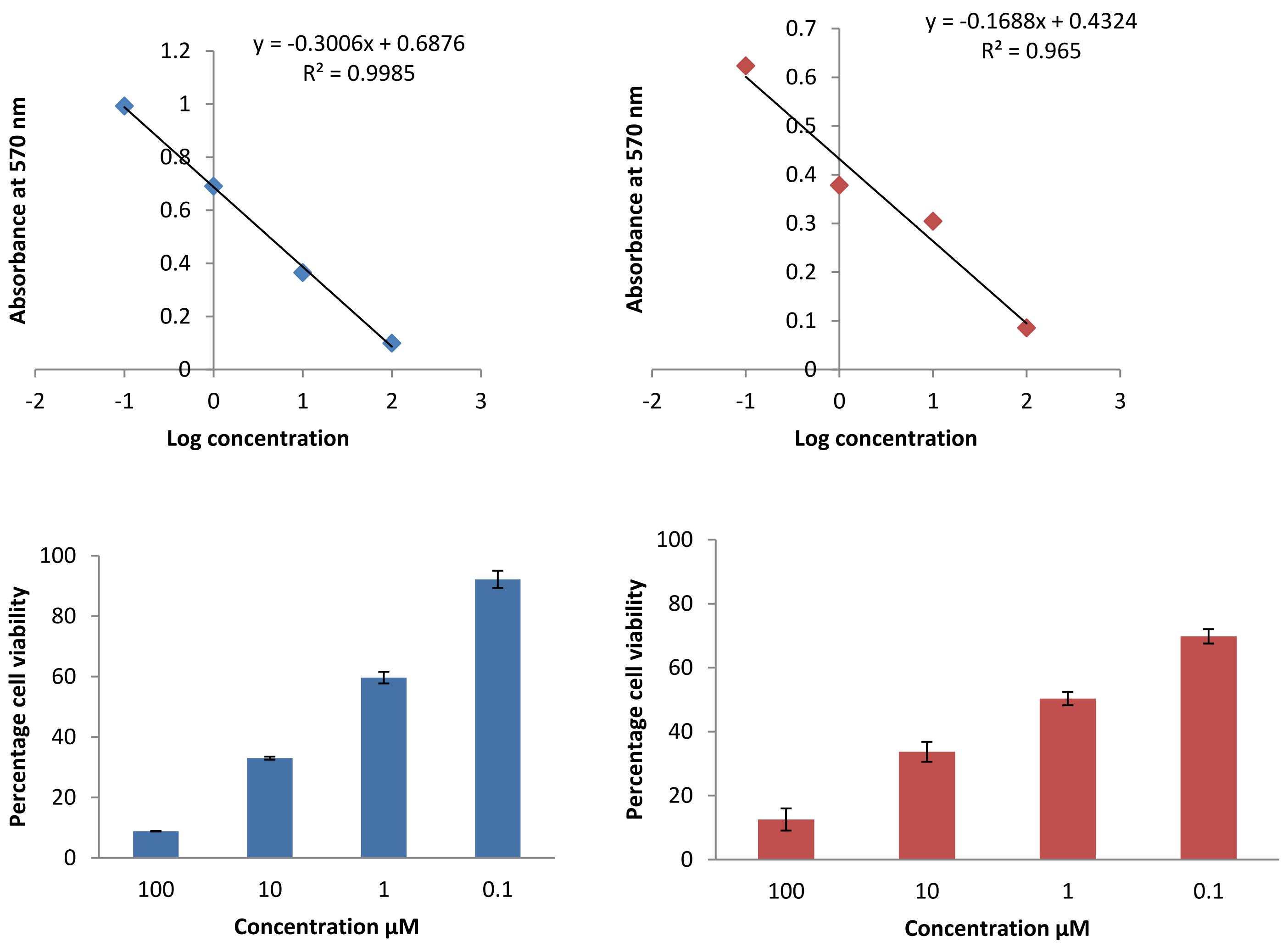

HeLa **MCF-7**

Figure 59. Linear regression plots and percentage cell viability graphs of HeLa and MCF-7 cells exposed to different concentrations of **3j**.

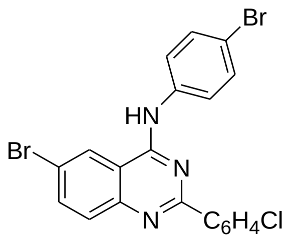

3k

Table 12. Percentage cell viability of HeLa and MCF-7 cells exposed to different concentrations of **3k**.

Conc. (μM)	%Viability HeLa	SD	%Viability MCF-7	SD
100	7.59	0.12	9.17	0.04
10	41.22	1.13	38.70	1.88
1	76.45	1.62	63.54	3.00
0.1	82.28	1.17	72.17	3.54

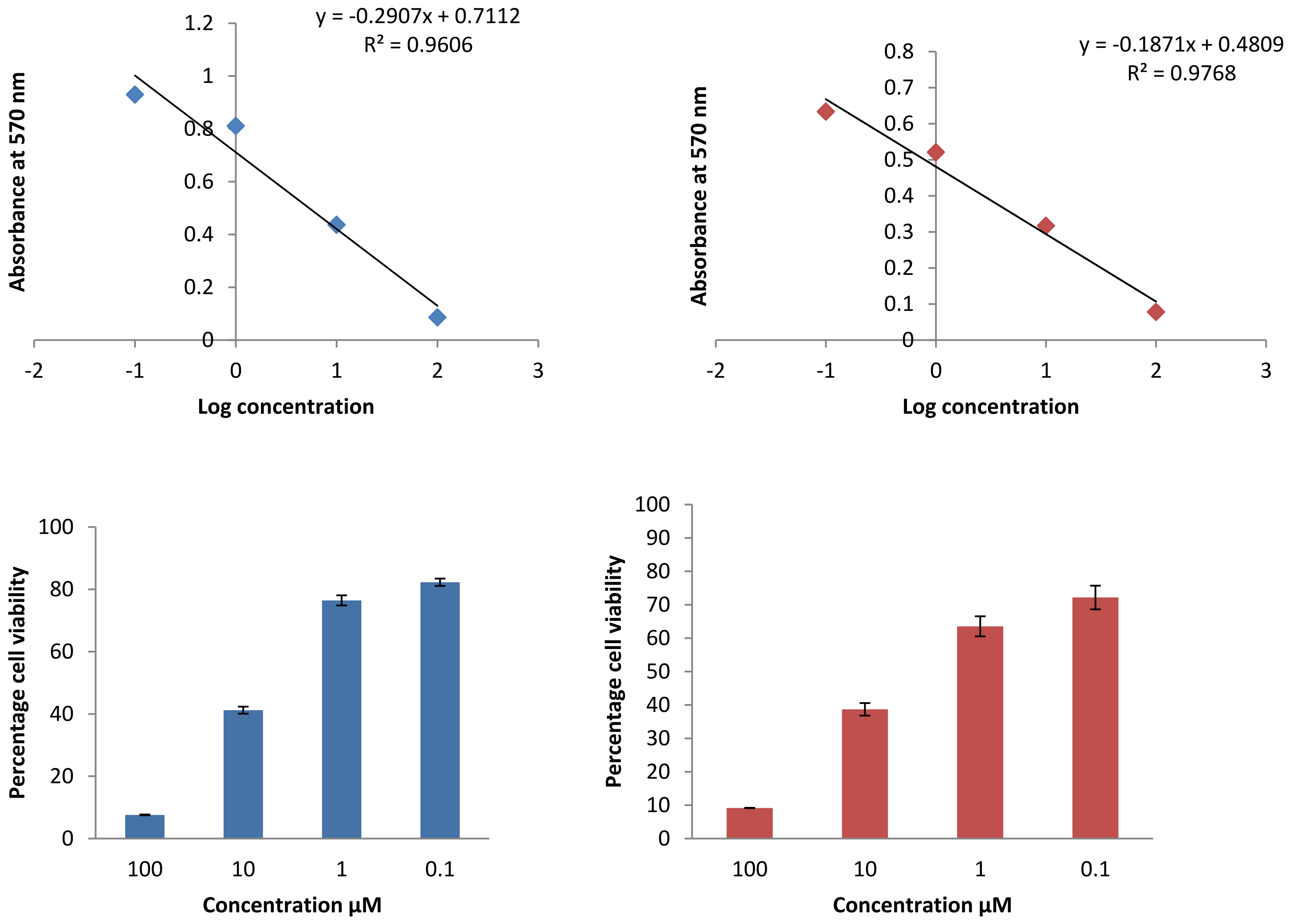

HeLa **MCF-7**

Figure 60. Linear regression plots and percentage cell viability graphs of HeLa and MCF-7 cells exposed to different concentrations of **3k**.

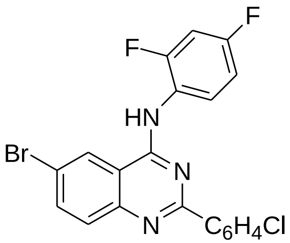

3l

Table 13. Percentage cell viability of HeLa and MCF-7 cells exposed to different concentrations of **3l**.

Conc. (μM)	%Viability HeLa	SD	%Viability MCF-7	SD
100	10.99	0.25	16.71	0.54
10	31.19	0.30	39.93	0.79
1	38.36	0.54	44.20	1.00
0.1	53.28	0.80	47.24	1.38

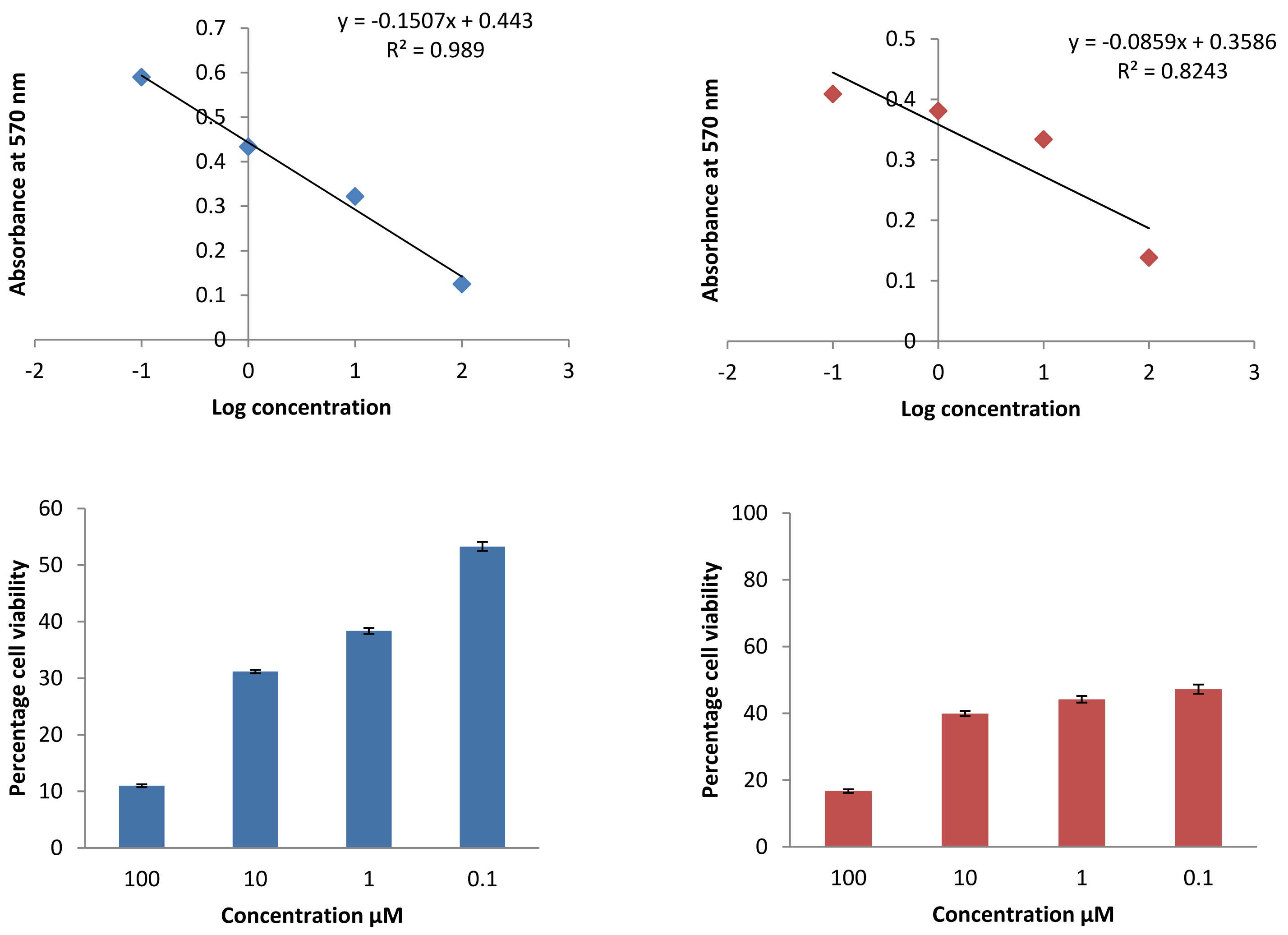

HeLa **MCF-7**

Figure 61. Linear regression plots and percentage cell viability graphs of HeLa and MCF-7 cells exposed to different concentrations of **3l**.

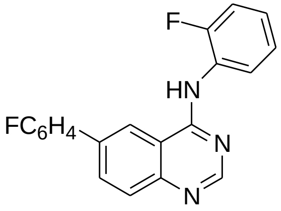

4a

Table 14. Percentage cell viability of HeLa and MCF-7 cells exposed to different concentrations of **4a**.

Conc. (µM)	%Viability HeLa	SD	%Viability MCF-7	SD
100	9.34	0.50	15.59	0.13
10	52.17	0.54	54.34	2..34
1	60.79	1.64	55.02	2.04
0.1	64.14	0.60	57.53	2.92

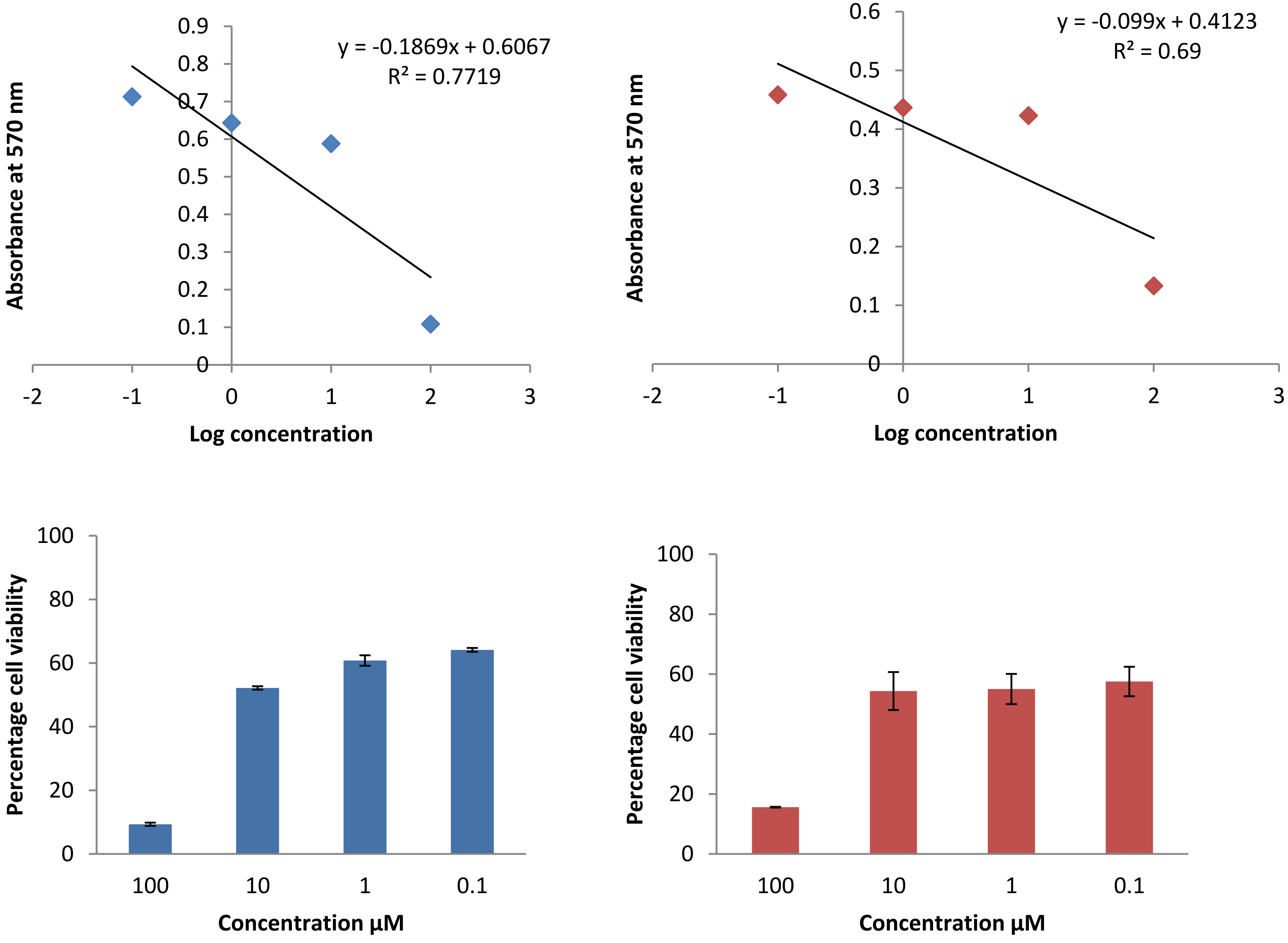

HeLa **MCF-7**

Figure 62. Linear regression plots and percentage cell viability graphs of HeLa and MCF-7 cells exposed to different concentrations of **4a**.

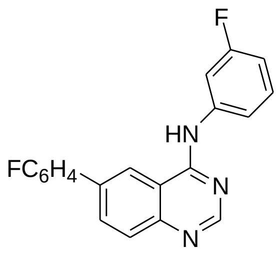

4b

Table 15. Percentage cell viability of HeLa and MCF-7 cells exposed to different concentrations of **4b**.

Conc. (µM)	%Viability HeLa	SD	%Viability MCF-7	SD
100	10.30	0.66	16.92	0.08
10	42.16	1.23	43.47	2.29
1	55.14	1.55	51.98	2.71
0.1	63.85	2.59	56.67	2.76

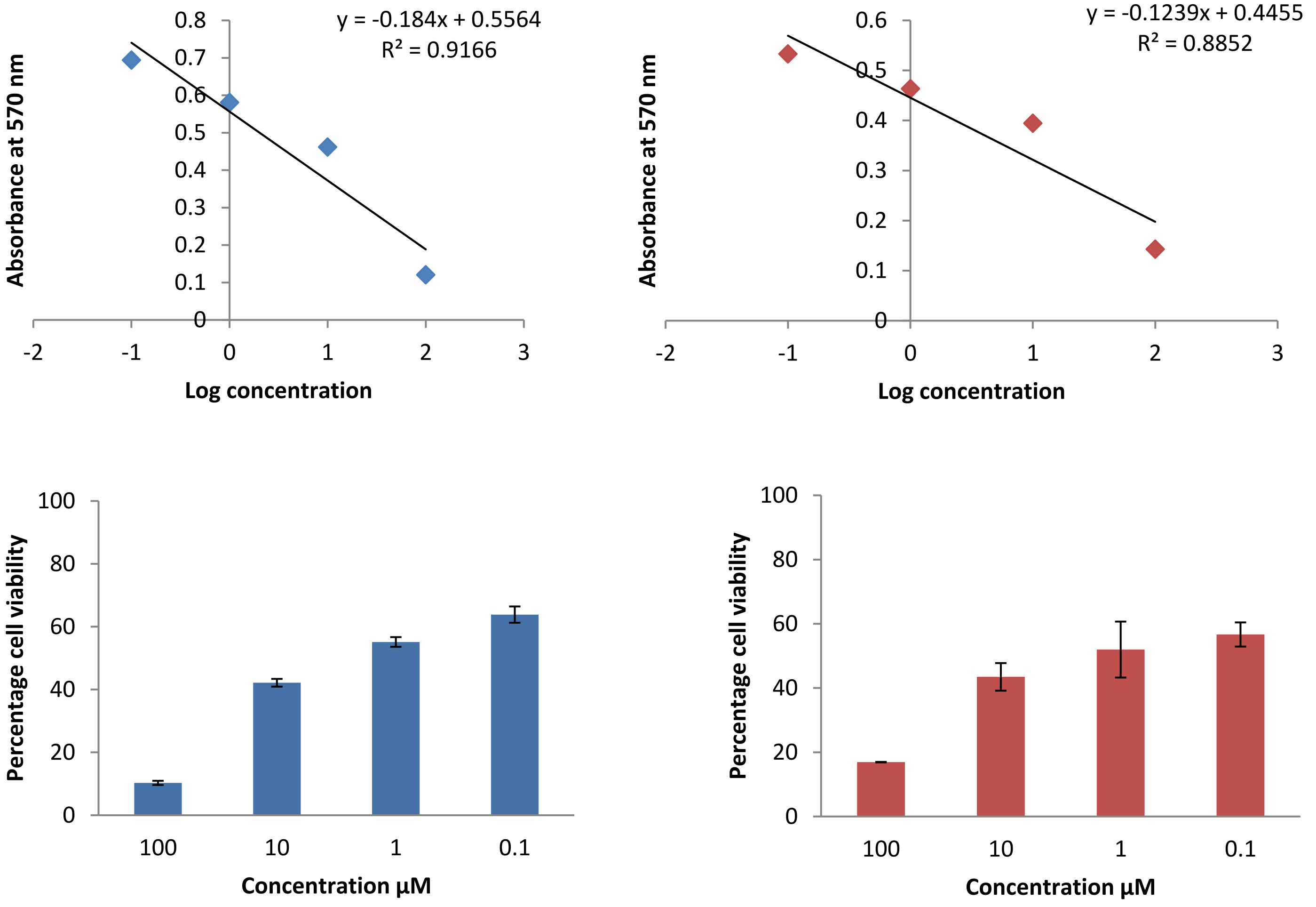

HeLa **MCF-7**

Figure 63. Linear regression plots and percentage cell viability graphs of HeLa and MCF-7 cells exposed to different concentrations of **4b**.

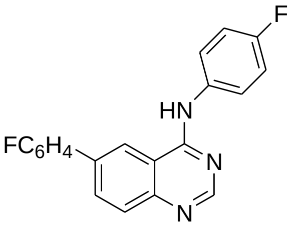

4c

Table 16. Percentage cell viability of HeLa and MCF-7 cells exposed to different concentrations of **4c**.

Conc. (µM)	%Viability HeLa	SD	%Viability MCF-7	SD
100	9.81	0.47	15.86	1.25
10	31.19	1.20	45.50	3.25
1	57.44	1.10	65.27	2.13
0.1	57.28	1.61	66.69	2.79

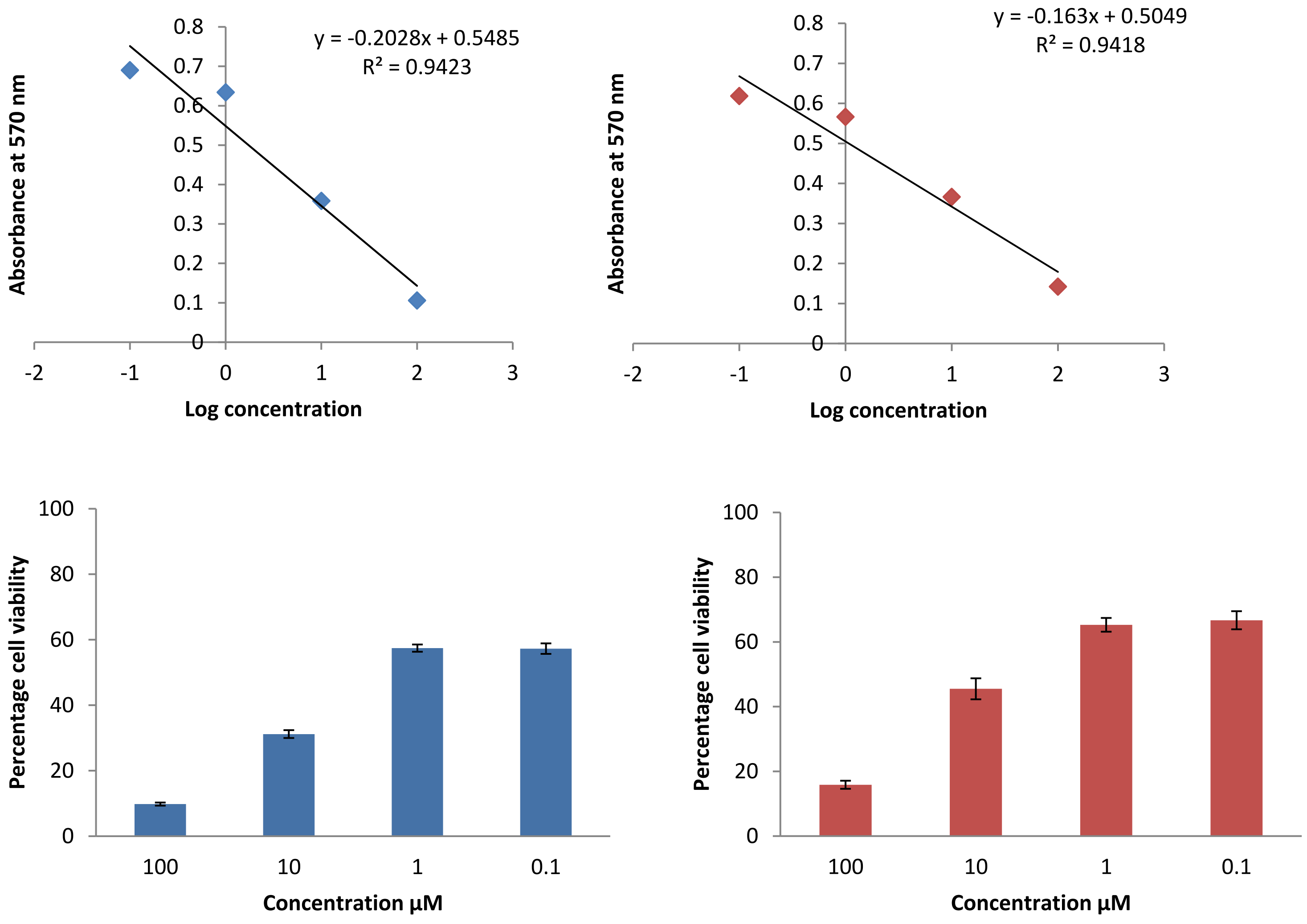

HeLa **MCF-7**

Figure 64. Linear regression plots and percentage cell viability graphs of HeLa and MCF-7 cells exposed to different concentrations of **4c**.

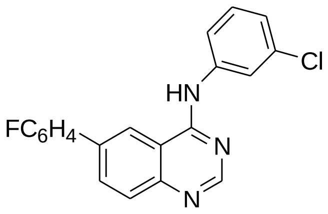

4d

Table 17. Percentage cell viability of HeLa and MCF-7 cells exposed to different concentrations of **4d**.

Conc. (μM)	%Viability HeLa	SD	%Viability MCF-7	SD
100	9.54	0.41	17.03	1.17
10	54.89	0.92	63.39	2.04
1	59.54	0.85	64.63	1.46
0.1	64.68	1.90	86.47	0.50

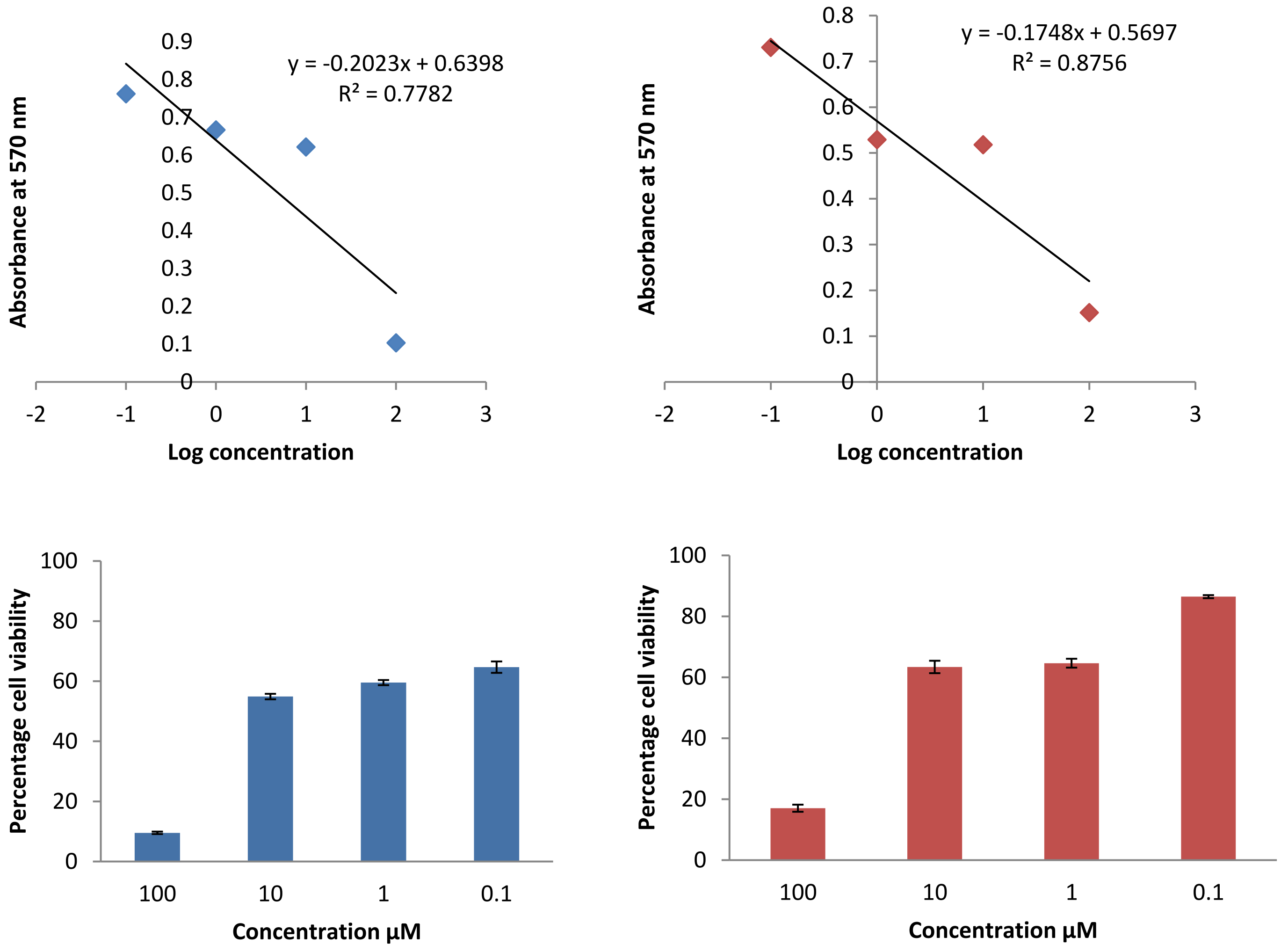

HeLa **MCF-7**

Figure 65. Linear regression plots and percentage cell viability graphs of HeLa and MCF-7 cells exposed to different concentrations of **4d**.

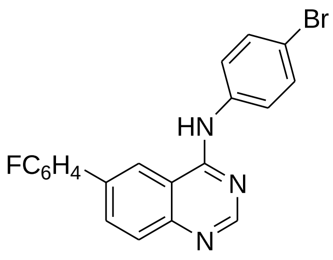

4e

Table 18. Percentage cell viability of HeLa and MCF-7 cells exposed to different concentrations of **4e**.

Conc. (μM)	%Viability HeLa	SD	%Viability MCF-7	SD
100	9.16	0.50	14.62	0.42
10	52.26	1.26	60.83	1.09
1	68.12	0.22	66.19	1.50
0.1	70.35	2.56	76.27	1.17

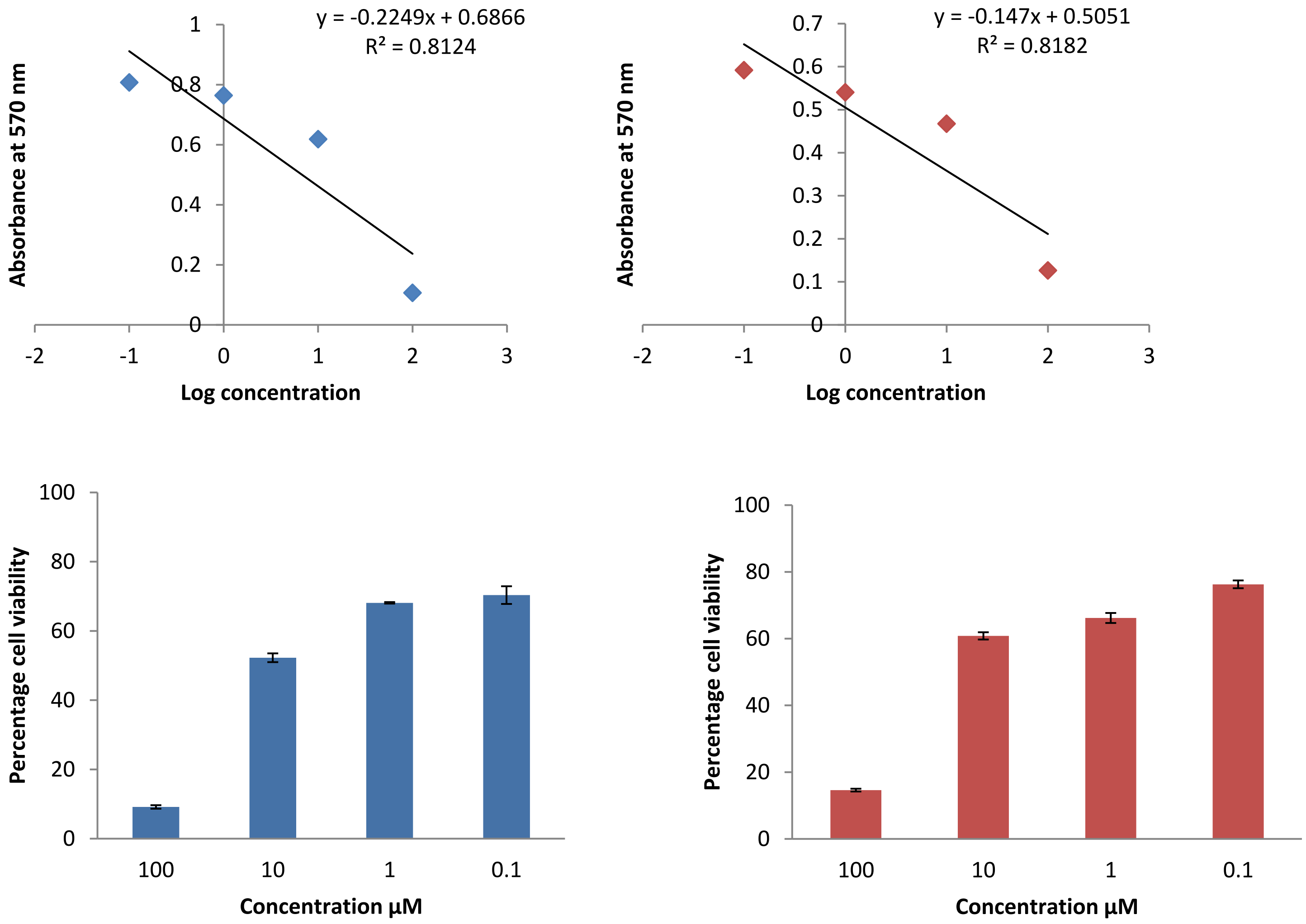

HeLa **MCF-7**

Figure 66. Linear regression plots and percentage cell viability graphs of HeLa and MCF-7 cells exposed to different concentrations of **4e**.

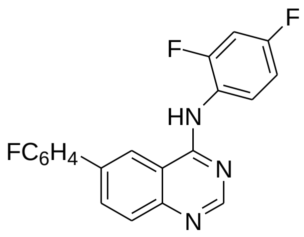

4f

Table 19. Percentage cell viability of HeLa and MCF-7 cells exposed to different concentrations of **4f**.

Conc. (μM)	%Viability HeLa	SD	%Viability MCF-7	SD
100	9.27	0.16	14.79	0.33
10	52.75	1.40	48.92	1.17
1	55.16	1.03	53.75	0.33
0.1	68.43	1.30	69.32	1.50

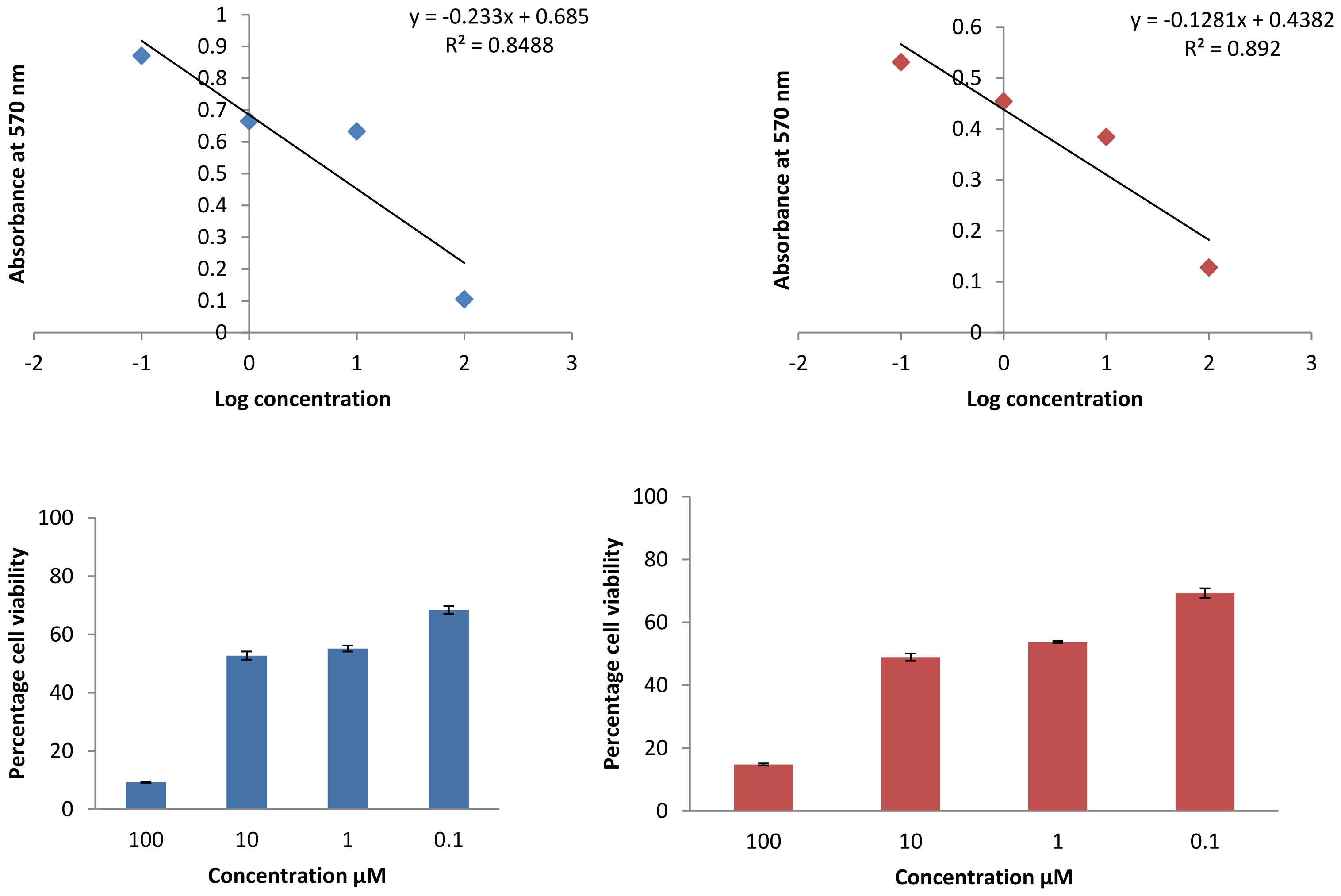

HeLa MCF-7

Figure 67. Linear regression plots and percentage cell viability graphs of HeLa and MCF-7 cells exposed to different concentrations of **4f**.

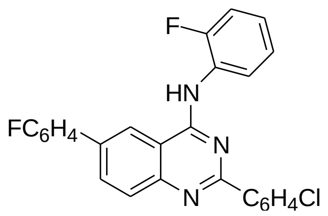

4g

Table 20. Percentage cell viability of HeLa and MCF-7 cells exposed to different concentrations of **4g**.

Conc. (μM)	%Viability HeLa	SD	%Viability MCF-7	SD
100	10.14	0.13	15.97	0.08
10	29.09	1.77	45.33	1.25
1	49.49	1.28	52.61	1.29
0.1	64.03	1.98	62.60	1.34

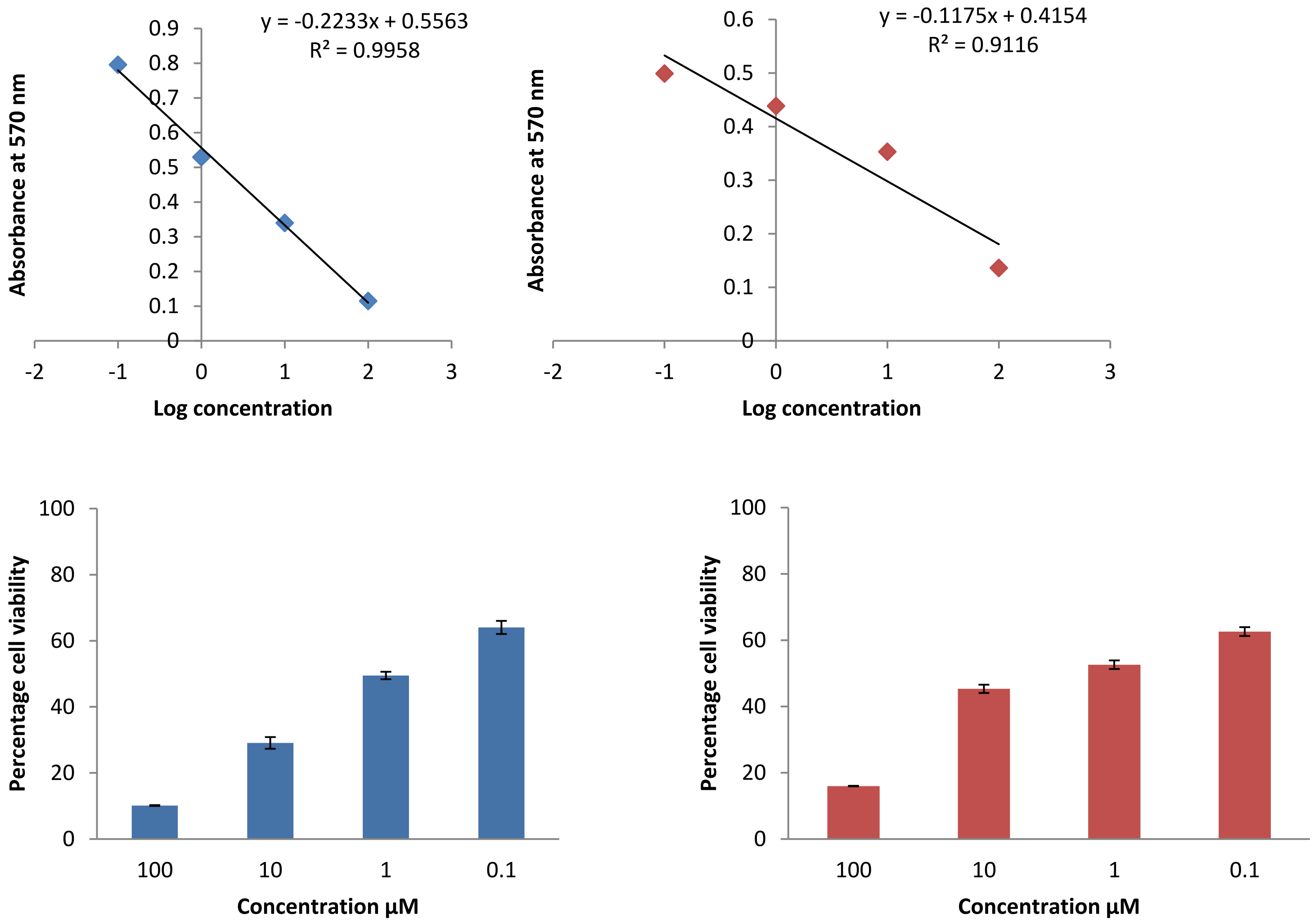

HeLa **MCF-7**

Figure 68. Linear regression plots and percentage cell viability graphs of HeLa and MCF-7 cells exposed to different concentrations of **4g**.

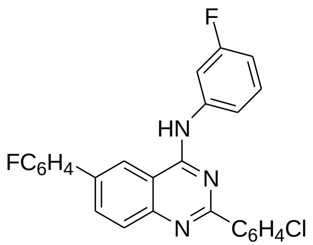

4h

Table 21. Percentage cell viability of HeLa and MCF-7 cells exposed to different concentrations of **4h**.

Conc. (µM)	%Viability HeLa	SD	%Viability MCF-7	SD
100	10.08	0.16	12.79	0.67
10	49.87	0.88	54.05	1.75
1	52.99	0.31	59.00	1.08
0.1	55.74	1.00	64.66	1.92

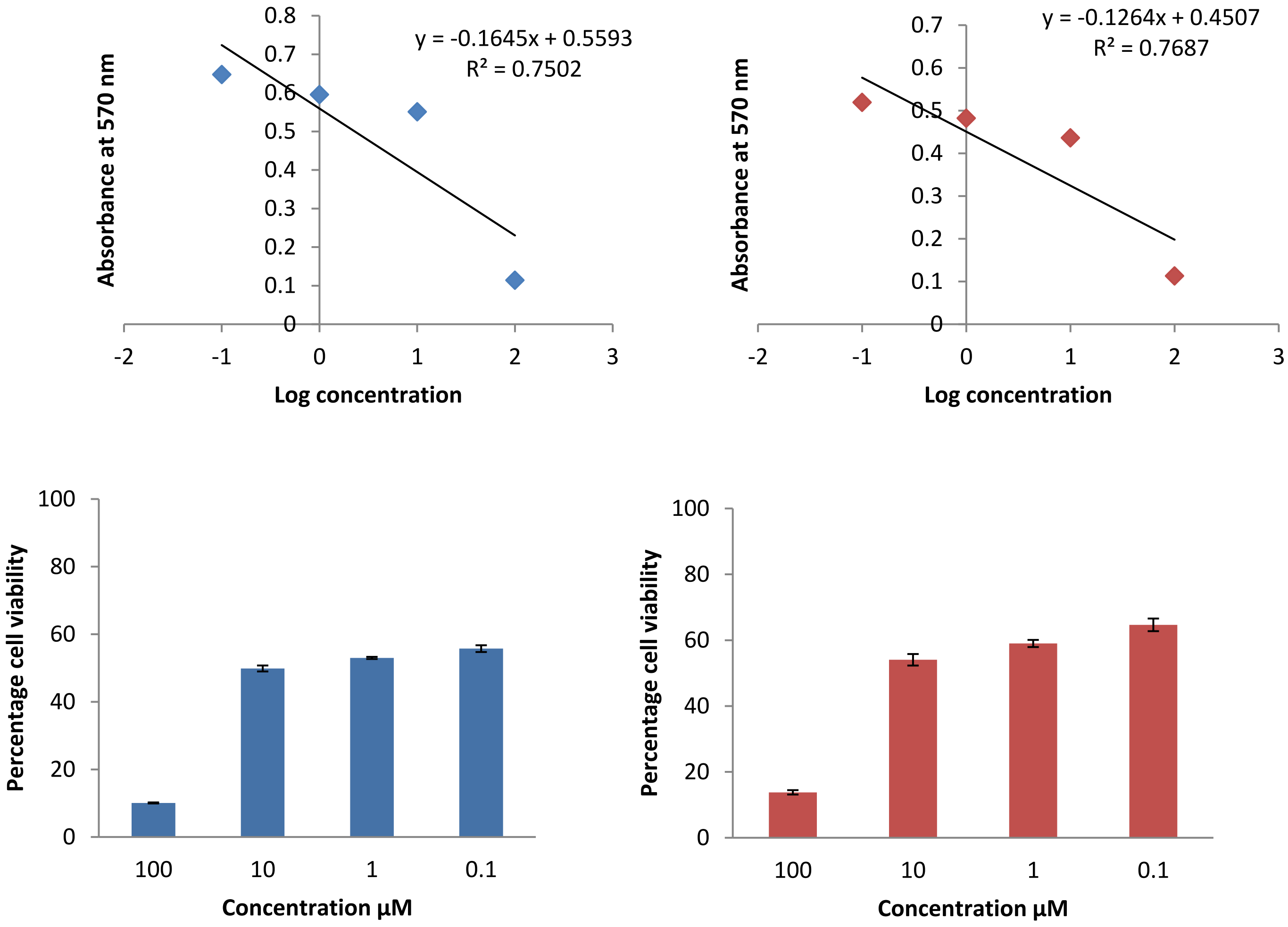

HeLa **MCF-7**

Figure 69. Linear regression plots and percentage cell viability graphs of HeLa and MCF-7 cells exposed to different concentrations of **4h**.

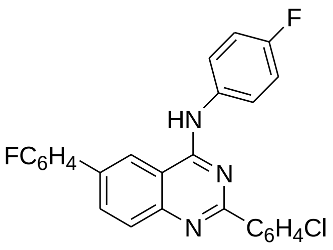

4i

Table 22. Percentage cell viability of HeLa and MCF-7 cells exposed to different concentrations of **4i**.

**Conc. (µM)**	**%Viability HeLa**	**SD**	**%Viability MCF-7**	**SD**
100	8.18	0.63	10.43	0.25
10	50.98	1.58	55.40	1.00
1	54.33	1.43	60.33	1.58
0.1	62.51	0.69	66.01	1.00

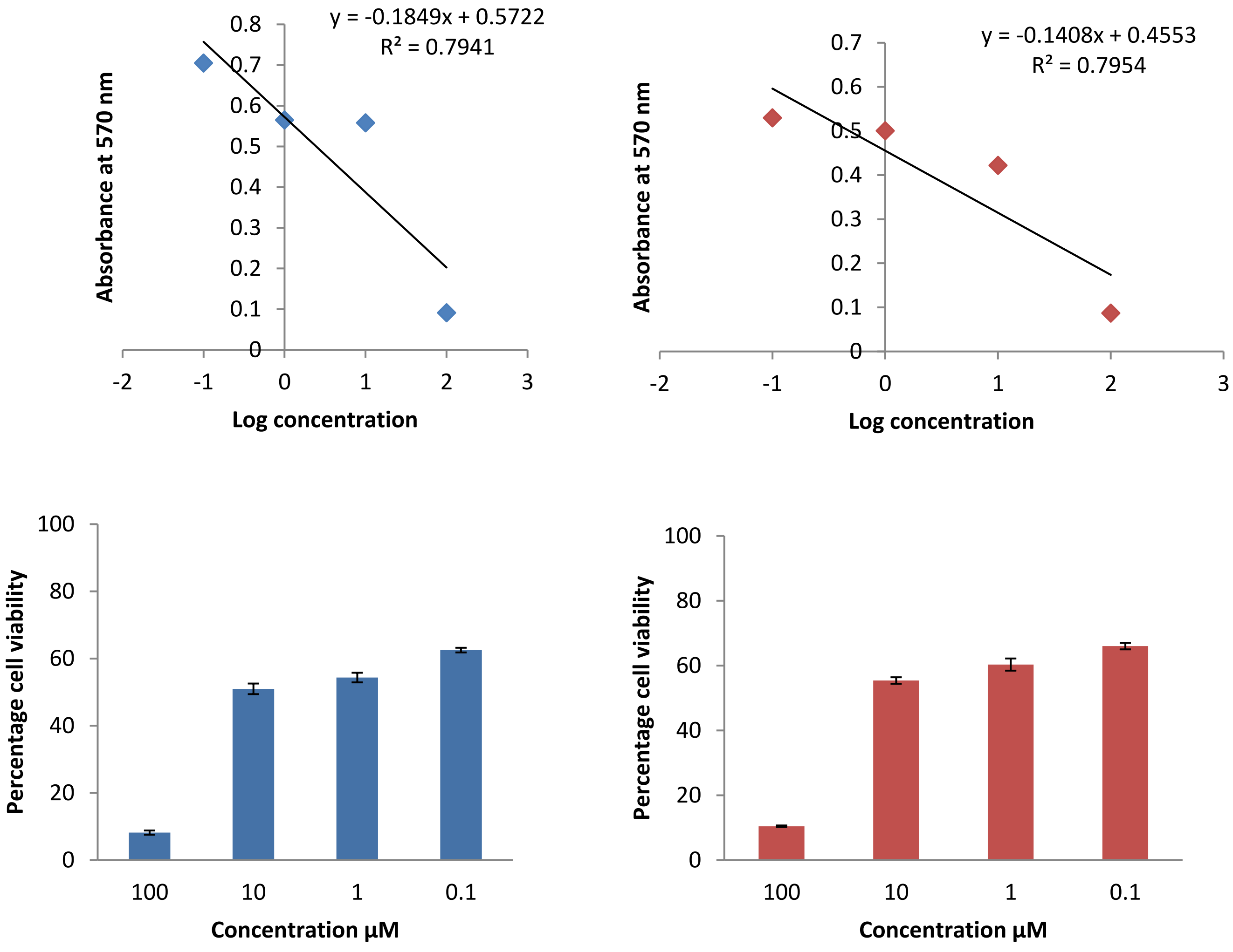

HeLa **MCF-7**

Figure 70. Linear regression plots and percentage cell viability graphs of HeLa and MCF-7 cells exposed to different concentrations of **4i**.

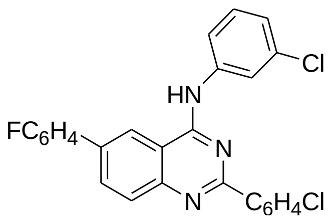

4j

Table 23. Percentage cell viability of HeLa and MCF-7 cells exposed to different concentrations of **4j**.

Conc. (μM)	%Viability HeLa	SD	%Viability MCF-7	SD
100	7.24	1.00	10.02	1.17
10	54.71	1.75	46.27	1.92
1	74.66	2.46	66.49	0.80
0.1	85.21	1.80	75.95	1.29

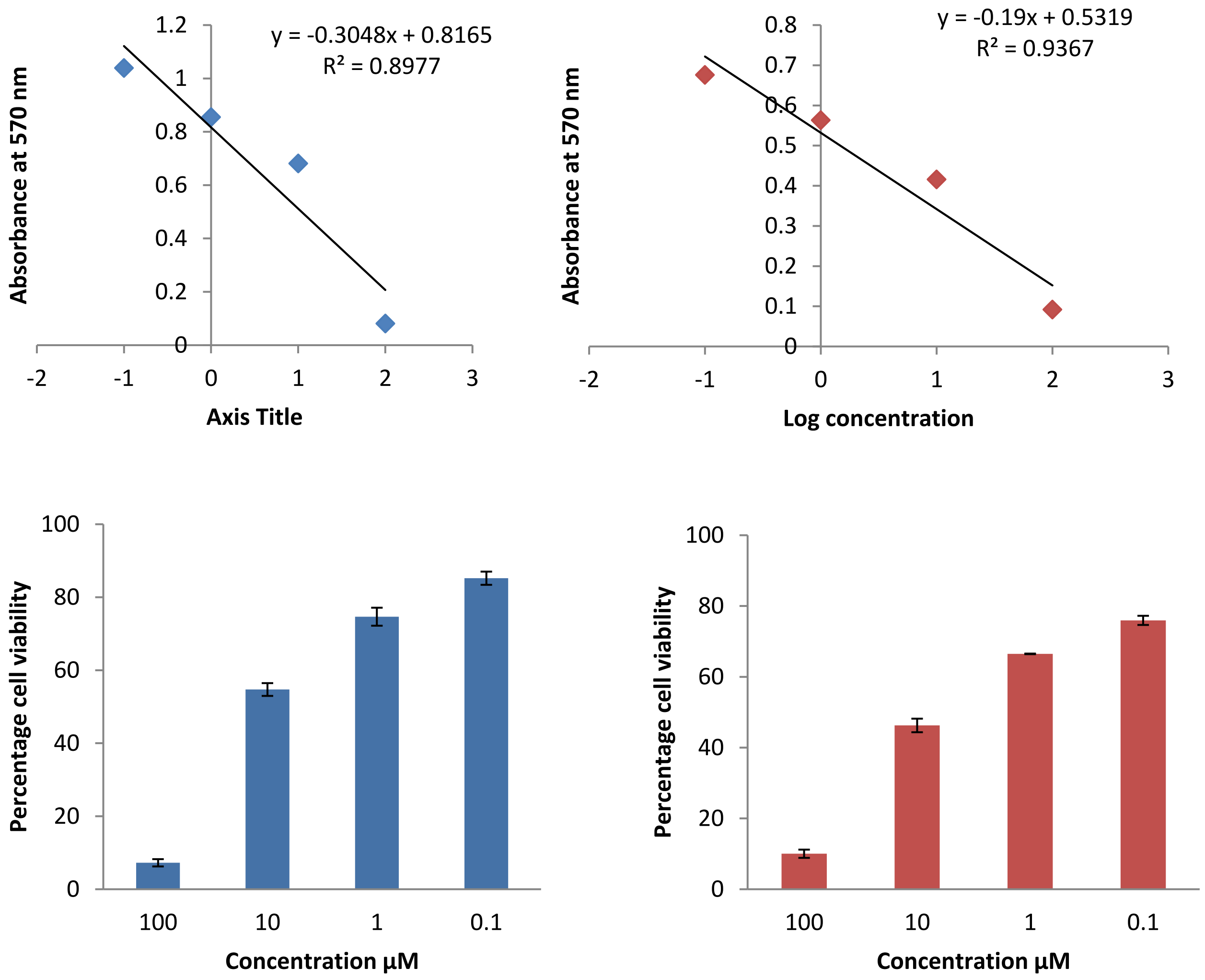

HeLa **MCF-7**

Figure 71, Linear regression plots and percentage cell viability graphs of HeLa and MCF-7 cells exposed to different concentrations of **4j**.

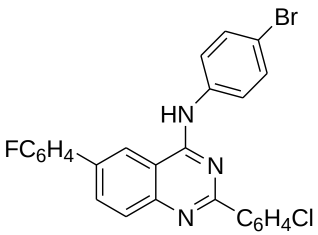

4k

Table 24. Percentage cell viability of HeLa and MCF-7 cells exposed to different concentrations of **4k**.

Conc. (µM)	%Viability HeLa	SD	%Viability MCF-7	SD
100	11.79	0.50	12.85	0.75
10	38.20	2.15	51.93	1.58
1	47.63	1.26	56.11	0.08
0.1	54.44	1.54	69.32	1.68

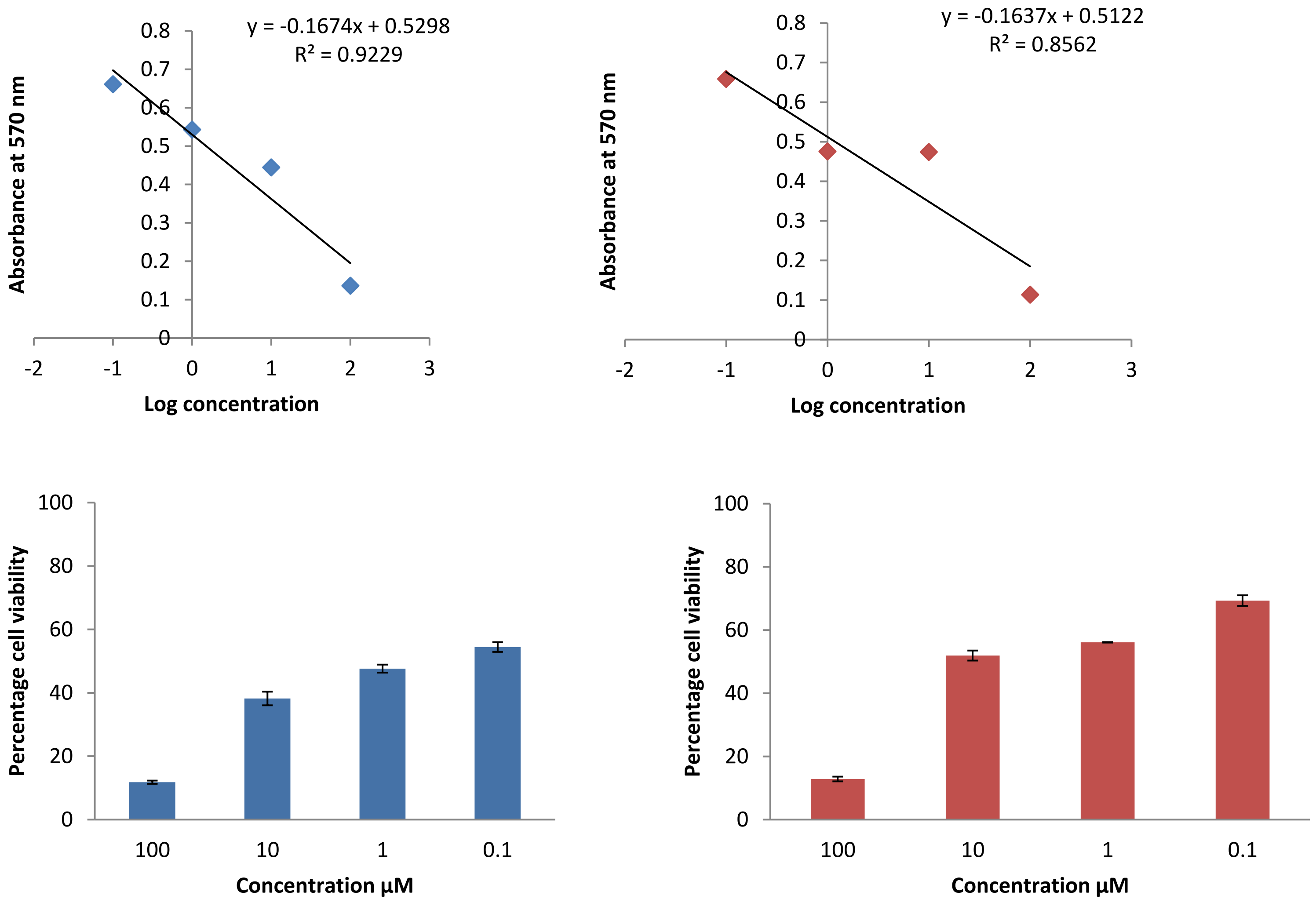

HeLa **MCF-7**

Figure 72. Linear regression plots and percentage cell viability graphs of HeLa and MCF-7 cells exposed to different concentrations of **4k**.

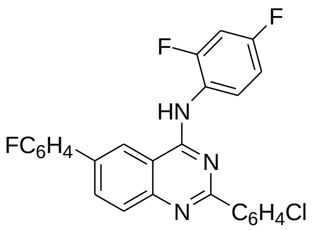

4l

Table 25. Percentage cell viability of HeLa and MCF-7 cells exposed to different concentrations of **4l**.

Conc. (μM)	%Viability HeLa	SD	%Viability MCF-7	SD
100	8.71	0.13	10.52	0.54
10	26.16	1.42	24.67	1.46
1	47.83	0.47	50.75	0.67
0.1	59.02	1.25	60.68	1.04

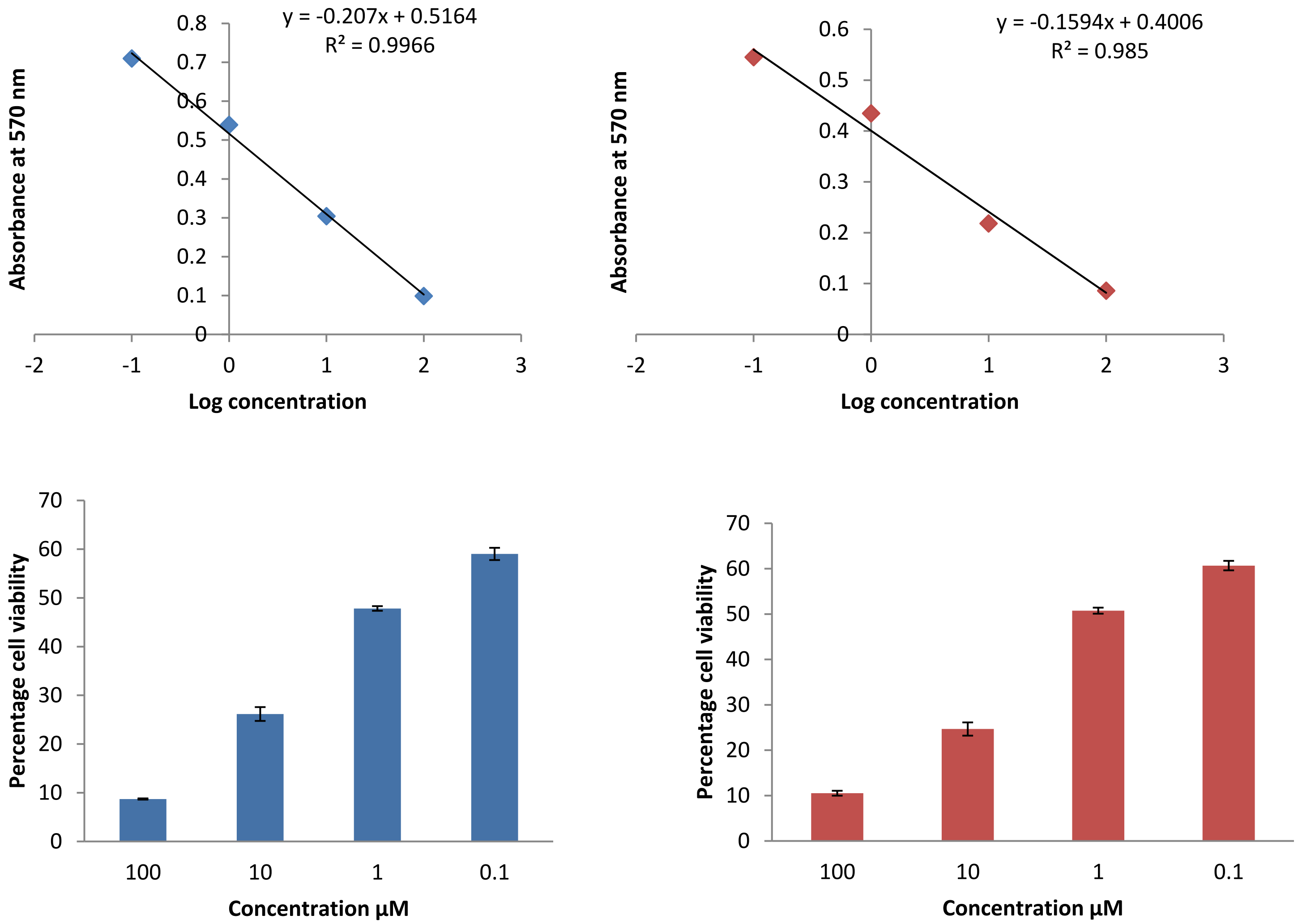

HeLa **MCF-7**

Figure 73. Linear regression plots and percentage cell viability graphs of HeLa and MCF-7 cells exposed to different concentrations of **4l**.

S3. Percentage Inhibition Curves of 3g, 3l, 4l, and Gefitinib (S3)
